# Gymnemantoside A Ameliorates Steroid‐Induced Skeletal Muscle Atrophy via Bridging Glucocorticoid and Insulin Receptor Signalling

**DOI:** 10.1002/jcsm.70118

**Published:** 2025-11-25

**Authors:** Eun‐Jin Park, Hyun‐Jun Kim, Sang‐Hoon Lee, Seri Choi, Thi‐Phuong Doan, Kyoung‐Hwan Joo, Jin‐Pyo An, Da‐Woon Jung, Darren Reece Williams, Won Keun Oh

**Affiliations:** ^1^ Research Institute of Pharmaceutical Sciences, College of Pharmacy Seoul National University Seoul Republic of Korea; ^2^ Department of Life Sciences, College of Life Sciences and Medical Engineering, New Drug Targets Laboratory Gwangju Institute of Science and Technology Gwangju Republic of Korea

**Keywords:** Akt/mTOR, autophagy, *Gymnema inodorum*, gymnemantoside A, natural products, sarcopenia, skeletal muscle atrophy

## Abstract

**Background:**

Skeletal muscle atrophy is a common condition caused by numerous factors, such as aging (termed sarcopenia), disease (e.g., cancer cachexia) or specific medications (such as glucocorticoids). There is no FDA‐approved drug for treating skeletal muscle atrophy. Previously, clinical studies had highlighted the anti‐diabetic properties of *Gymnema inodorum* extract, an indigenous medicinal plant and functional food in Thailand. However, these studies did not identify the active compound(s) responsible for its therapeutic effects. This research aimed to identify safe natural product‐derived compounds from clinically relevant plants such as *Gymnema inodorum* that bridge the connection between metabolic diseases and skeletal muscle atrophy.

**Methods:**

Utilizing the dexamethasone (Dex) induced skeletal muscle atrophy model, the major active compounds were isolated using bioactivity‐guided screening. The chemical structure of active compound(s) was elucidated using various spectroscopic methods, including HRESIMS, 1D and 2D NMR. Active compound(s) were tested in cell‐based and murine models of skeletal muscle atrophy. Insulin signaling and autophagy activity were assessed by western blotting and the mRFP‐GFP‐LC3 (ptf‐LC3) probe. Docking studies determined the binding affinities of active compound(s) for the insulin and glucocorticoid receptors.

**Results:**

We determined a new, previously undescribed methyl anthranilate‐conjugated oleanane bioactive compound, termed gymnemantoside A. Treatment with gymnemantoside A recovered myotube diameter and skeletal muscle fibre CSA in atrophy models and downregulated expression of the atrogenes, atrogin‐1 and MuRF‐1 (myotube diameter: +33.82%, *p* < 0.05, CSA: +128%, *p* < 0.01; atrogin‐1: −58.70%, MuRF‐1: −57.32%, *p* < 0.05). Gymnemantoside A also reduced autophagy levels both in vitro and in vivo (−65.56%, *p* < 0.01), increased expression of insulin growth factor‐1 (+78.05%, *p* < 0.05) and the insulin receptor (+56.42%, *p* < 0.05), and recovered activity of the downstream Akt/mTOR‐mediated insulin signalling pathway. Molecular docking analysis with gymnemantoside A and standard receptor ligands revealed that gymnemantoside A could interact with the insulin receptor tyrosine kinase and prevent Dex binding with the glucocorticoid receptor. In the murine atrophy model, gymnemantoside A treatment enhanced exercise endurance in the rotarod test (+260%, *p* < 0.05) and the mass of the predominantly fast fibre type TA muscle (+116%, *p* < 0.05).

**Conclusions:**

A newly identified bioactive compound gymnemantoside A, isolated from *Gymnema inodorum*, was structurally characterized and showed efficacy in Dex‐induced muscle atrophy models. Gymnemantoside A produces anti‐muscle atrophy effects by a hitherto unreported mechanism: modulation of the insulin receptor kinase domain and activation of downstream signalling, alongside competitive inhibition of the glucocorticoid receptor, which has been implicated in multiple forms of skeletal muscle wasting.

## 1 Introduction

The rapid increase in the global elderly population is leading to significant social challenges that need to be addressed by health care systems and societies as a whole [1]. Aging negatively impacts both muscle mass and bone density, potentially leading to conditions such as osteoporosis, fractures and sarcopenia (aging‐related skeletal muscle atrophy) [2]. Muscle atrophy in sarcopenia results from a combination of different mechanisms. These include hormonal level fluctuations, increased glucocorticoid levels, increased rates of muscle protein degradation via activation of ubiquitin‐proteolytic pathways, and inhibition of the Akt‐mammalian target of rapamycin (mTOR). Despite the increasing socio‐economic burden of sarcopenia, the US Food and Drug Administration (FDA) has yet to approve any pharmaceutical drug treatments.

*Gymnema inodorum* (Lour.) Decne, belonging to the Apocynaceae family, is a creeping vine native to Southeast Asia. It is particularly renowned in Thailand for its medicinal properties, especially hypoglycaemic effects, and has been patented for use in herbal tea preparations, although the active compound(s) were unknown [3]. Extensive research has been conducted on the chemical components of *G. inodorum*, revealing a variety of triterpenoid compounds that either contain a sugar moiety or *N*‐methyl anthranilate [4, 5]. However, despite these findings, the active compound in *G. inodorum* that is responsible for its hypoglycemic activity, and the exact mechanism by which it lowers plasma glucose levels, remain a subject of investigation.

In our previous study, we reported oleanane triterpenoids with either a sugar or methyl anthranilate moiety isolated from *G. inodorum* leaves and evaluated their biological effects. One compound with a methyl anthranilate moiety produced significant glucose uptake activity in 3 T3‐L1 adipocyte cells and promoted cell proliferation [4]. This compound was termed (3β,16β,22α)‐22‐(*N*‐methylanthraniloxy)‐16,23,28‐trihydroxyolean‐12‐en‐3‐yl‐*O*‐β‐*d*‐glucopyranosiduronic acid (Gi8). This finding, along with other supporting research, led us to hypothesize that the glucose uptake and cell proliferation activities of the Gi8 compound might also indicate potential anti‐aging effects and could be beneficial in the treatment of degenerative diseases such as sarcopenia.

In this study, Dex‐treated murine C2C12 myotubes and mice were used as models for sarcopenia due to their known similarities with disease progression [6]. A purified novel compound isolated from *G. inodorum* with anti‐muscle atrophy activity was identified and subjected to rigorous evaluation in these models. This approach allowed for a detailed investigation into the potential benefits and mechanisms of action of the *G. inodorum* extract and its components.

## 2 Materials and Methods

### 2.1 Quantitative Analysis of Gymnemantoside A

Quantitative HPLC analysis was performed using an Agilent 1100 Series system equipped with a C18 column and a diode‐array detector. Gymnemantoside A (1 mg) was dissolved in 1 mL of methanol to prepare a stock solution, which was serially diluted to generate six calibration levels (0.03125–1.00 mg/mL). The column temperature was maintained at 30°C. The mobile phase consisted of acetonitrile and water containing 0.1% formic acid (v/v), delivered as a linear gradient from 10:90 to 90:10 over 30 min at a flow rate of 1.0 mL/min. Absorbance was monitored at 254 nm for quantitation of gymnemantoside A.

Identification of Compound 1 (gymnemantoside A): the leaves of *G. inodorum* (5 kg) were extracted with 70% ethanol, the crude extract was partitioned with n‐hexane, EtOAc, n‐BuOH and water, the n‐BuOH layer was further separated by RP‐C18 MPLC followed by Sephadex LH‐20 to yield subfractions B1–B4, and the major constituent of B3 was identified as compound 1 (gymnemantoside A) by HRESIMS together with 1D and 2D NMR as described in the main text; quantitative HPLC showed that B3 contained the highest level of this compound, whereas the other fractions contained smaller amounts, and because B3 also delivered the most pronounced biological efficacy, we selected it for isolation and mechanism work while the remaining fractions, which contained gymnemantoside A only at lower levels, were not pursued further.

### 2.2 Cell Culture and Differentiation

C2C12 murine myoblast cells were cultured in Dulbecco's Modified Eagle's Medium (DMEM, Hyclone, UT, USA) supplemented with 10% fetal bovine serum (FBS) (Hyclone) and 100 U/mL penicillin and 100 mg/mL streptomycin (PenStrep; Hyclone) at 37°C in a 5% CO_2_ atmosphere. Myoblasts were induced to differentiate by replacing the medium with DMEM supplemented with 2% horse serum (Gibco, NY, USA) and PenStrep.

HEK293 cells stably expressing GFP‐LC3 were generously provided by Professor Junsoo Park (Yonsei University, Republic of Korea). All cells were maintained in DMEM supplemented with 10% FBS and PenStrep. Cultures were passaged every 2 days and used at 70%–80% confluence.

### 2.3 Animal Studies

Animal studies were carried out under the auspices of the Institute for Laboratory Animal Research Guide for the Care and Use of Laboratory Animals, and approved by the Gwangju Institute of Science and Technology Animal Care and Use Committee (study approval number GIST‐2022‐057). The studies have been approved by the appropriate ethics committee and have therefore been performed in accordance with the ethical standards laid down in the 1964 Declaration of Helsinki and its later amendments.

### 2.4 Dexamethasone‐Induced Murine Model of Skeletal Muscle Atrophy

12‐weeks‐old male C57BL/6J mice were treated with drugs as follows: (1) injection of vehicle (5% DMSO, 5% Tween80, PBS) alone, (2) 15 mg/kg Dex dissolved in vehicle, (3) injection of 15 mg/kg Dex and 2.5 mg/kg GmA, (4) injection of 15 mg/kg Dex and 5 mg/kg GmA (*n* = 5 total per group). Mice were treated by intraperitoneal (IP) injection every 24 h for 14 days and then assessed for skeletal muscle function and condition.

### 2.5 Immobilization Model

Immobilization was carried out as previously described [7]. In brief, 14‐week‐old male C57BL/6J mice were anaesthetised using isoflurane, and both hind limbs were secured with plastic EP‐tubes (Axygen, MCT‐175‐C). To prevent slippage of the tubes, the junction between the tube and hind limbs was wrapped with insulating tape. GmA treatment was carried out as follows: (1) vehicle alone and (2) 5 mg/kg GmA via IP delivery every 24 h (*n* = 5 per group). The mice were checked daily and euthanized at 14 days.

### 2.6 In Silico ADME

The GmA structure was drawn in ChemDraw and exported as a.mol file. This file was uploaded to the web‐based ADME prediction platform SwissADME [8] to obtain the calculated parameters. The in silico ADME results are summarized in Table S7.

### 2.7 ADME Analysis of Blood, Liver and Skeletal Muscle Tissues

Serum, liver and muscle extraction method: For serum samples, 30 μL of serum was mixed with 1 mL of methanol and vortexed. The mixture was centrifuged at 15,000 rpm for 3 min at 4°C. The resulting supernatant was filtered through a 0.2 μm nylon filter prior to LC analysis. Liver samples were homogenized at a frequency of 30 Hz for 2 min, while other tissue samples were homogenized at 30 Hz for 4 min. All homogenized samples were then sonicated for 20 min. Following sonication, the samples were centrifuged at 15,000 rpm for 3 min at 4°C, and the supernatants were filtered through a 0.2 μm nylon filter prior to LC analysis.

LC condition: The sample was analysed using a Xevo G3 QTOF mass spectrometer (Waters) equipped with an ACQUITY BEH C18 column (2.1 × 150 mm, 1.7 μm particle size). The gradient system was as follows: 0–1 min 10% B, 1–9 min 10%–98% B, 9–12 min 98% B, and 12.1–15 min 10% B. A flow rate of 0.4 mL/min was used, with an injection volume set at 2 μL. The ESI condition was set as follows: capillary voltage 2.5 kV for positive ion mode, cone voltage 40 V, source temperature 120°C, desolvation gas temperature 350°C, cone gas flow 50 L/h, and desolvation gas flow 800 L/h. During data acquisition, centroiding was performed using an independent reference lock‐mass ion through the LockSpray interface to maintain high mass accuracy and precision.

### 2.8 Statistical Analysis

All data were calculated using the mean ± SD from the three independent experiments. Significant differences between groups were determined using either one‐way analysis of variance (ANOVA) or the Student's *t*‐test. Statistical significance was accepted at *p < 0.05, **p < 0.01, and ***p < 0.001. All presented data were repeated at least three times to ensure reliability. The error bars in the graphs represent the standard deviation.

Further details of the materials and methods are provided in the Supporting Information.

## 3 Results

### 3.1 Muscle Atrophy‐Guided Isolation of Bioactive Components

**FIGURE 1:**
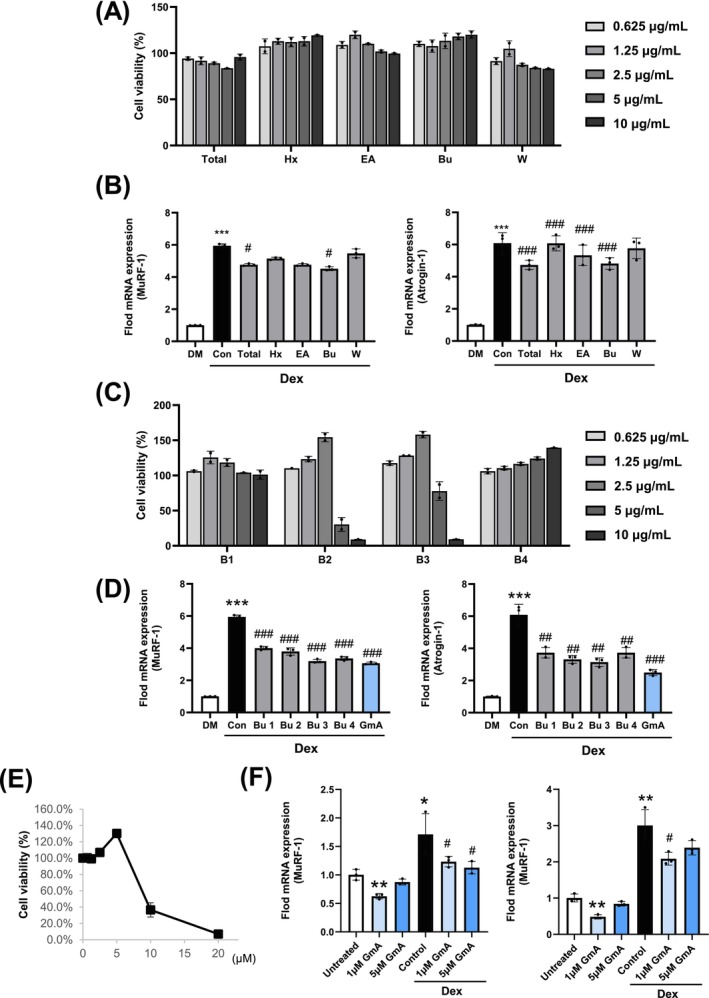
Bioactivity‐guided isolation of GmA. (A) Cell viability analysis of *G. inodorum* extracts and fractions in C2C12 murine myotubes. (B) Expression levels of muscle atrophy markers (MuRF‐1 and atrogin‐1) in C2C12 myotubes following treatment with the extracts and fractions. (C) Evaluation of cell viability in C2C12 myotubes after subfraction treatment. (D) Expression levels of MuRF‐1 and atrogin‐1 genes in C2C12 myotubes after treatment with subfractions or gymnemantoside A (GmA). (E) Cell viability of GmA in C2C12 myotubes. (F, G) Expression levels of MuRF‐1 and atrogin‐1 genes in the myotubes following treatment with GmA. Statistical significance is indicated as follows: *p < 0.05, **p < 0.01, ***p < 0.001 compared with the control (untreated) group. #*p* < 0.05, ##*p* < 0.01, ###*p* < 0.001 compared with the Dex treated myotubes (*n* = 3 per experimental group).

Bioactive components of *G. inodorum* were isolated using bio‐guided fractionation. Cell viability in C2C12 myoblasts was assessed using the MTT assay. None of the tested samples exhibited cell cytotoxicity, and the hexane and butanol fractions increased C2C12 myoblast viability dose‐dependently (Figure 1A). Treatment with the total extract or butanol fraction resulted in a significant downregulation of the atrogenes muscle RING‐finger protein‐1 (MuRF‐1) and atrogin‐1 (Figure 1B). Subfractions B2 and B3 of the butanol fraction significantly enhanced cell viability (Figure 1C). In Dex‐treated myotubes, subfraction B3 induced the greatest down‐regulation of MuRF‐1 and atrogin‐1 expression (Figure 1D). In addition, subfraction B3 showed no cytotoxicity at 2.5 μg/mL and, in the MTT assay, increased C2C12 myoblast viability to 158% of the untreated control, greater than was observed with subfraction B4. Although insulin‐receptor signalling was not the initial focus, it was reasoned that a fraction capable of eliciting an insulin‐like stimulus could also promote myogenesis and thus mitigate muscle atrophy. This idea is consistent with reports that insulin elevates C2C12 viability to ~150% of baseline [9, 10]. B4 also enhanced C2C12 viability, but the increase at 2.5 μg/mL was smaller (116%). Achieving a B3 level response with B4 required ≥ 10 μg/mL treatment in pilot tests, a range at which cytotoxicity and potential off‐target effects began to emerge. Therefore, subfraction B3 provided the best efficacy–safety balance and was prioritized for isolation and mechanistic studies.

The compound gymnemantoside A (GmA), isolated from subfraction B3, downregulated MuRF‐1 and atrogin‐1 expression (Figure 1D) and increased cell viability (Figure 1E); 1 μM GmA treatment inhibited MuRF‐1 and atrogin‐1 expression in Dex‐treated myotubes (Figure 1F). Quantification across subfractions showed that B3 contained the highest GmA content among B1–B4 (Figure S2).

**FIGURE 2:**
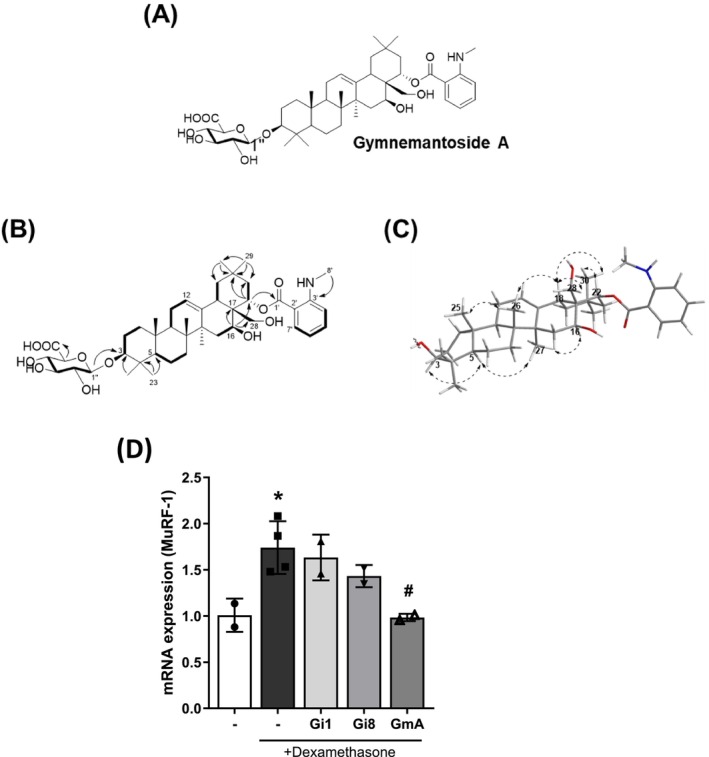
Chemical structure elucidation of gymnemantoside A (GmA): (A) structure of GmA. (B) Key HMBC and COSY correlations. (C) Key NOESY correlation. (D) qPCR analysis of MuRF‐1 expression in C2C12 myotubes treated as follows: (1) untreated control, (2) 10 μM dexamethasone (Dex), (3) Dex plus 5 μM Gi1 for 24 h, (4) Dex plus 5 μM Gi8 for 24 h, (5) Dex plus 5 μM gymnemantoside A (GmA) for 24 h. Statistical significance is indicated as follows: *p < 0.05 compared with the control (untreated) group. #*p* < 0.05 compared with the Dex treated myotubes (*n* = 2‐4 per experimental group).

GmA (**1**) (Figure 2A–C and Figures S1 and S2): White amorphous powder; ${\left[\alpha \right]}_{D}^{25}$ + 12.7 (*c* 0.1, MeOH); UV (MeOH) *λ*_max_ (log *ε*) 223 (1.72), 257 (1.24), 357 (1.10); IR (KBr) *ν*_max_ 3365, 2941, 2873, 356, 2312, 1751, 1672, 1608, 1514, 1427, 1362, 1240, 1160, 1077, 1027, 954, 752 cm^−1^; ^13^C and ^1^H NMR data, see Tables S2–S4; HRESIMS *m/z* 784.4612 [M + H]^+^ (calcd for C_44_H_66_NO_11_, 784.4636).

The potential of GmA as a therapeutic compound for skeletal muscle atrophy was compared with compound Gi8, which was previously derived from *G. inodorum* and shown to possess insulin mimetic activity [4], and compound Gi1, which is a diglycoside form of GmA (Figure S3). It was observed that compounds Gi1 and Gi8 had no significant effect on MuRF‐1 upregulation, whereas GmA treatment significantly reduced MuRF‐1 expression (Figure 2D).

### 3.2 GmA Treatment Prevents Myotube Atrophy and the Upregulation of Muscle Atrophy Markers

**FIGURE 3:**
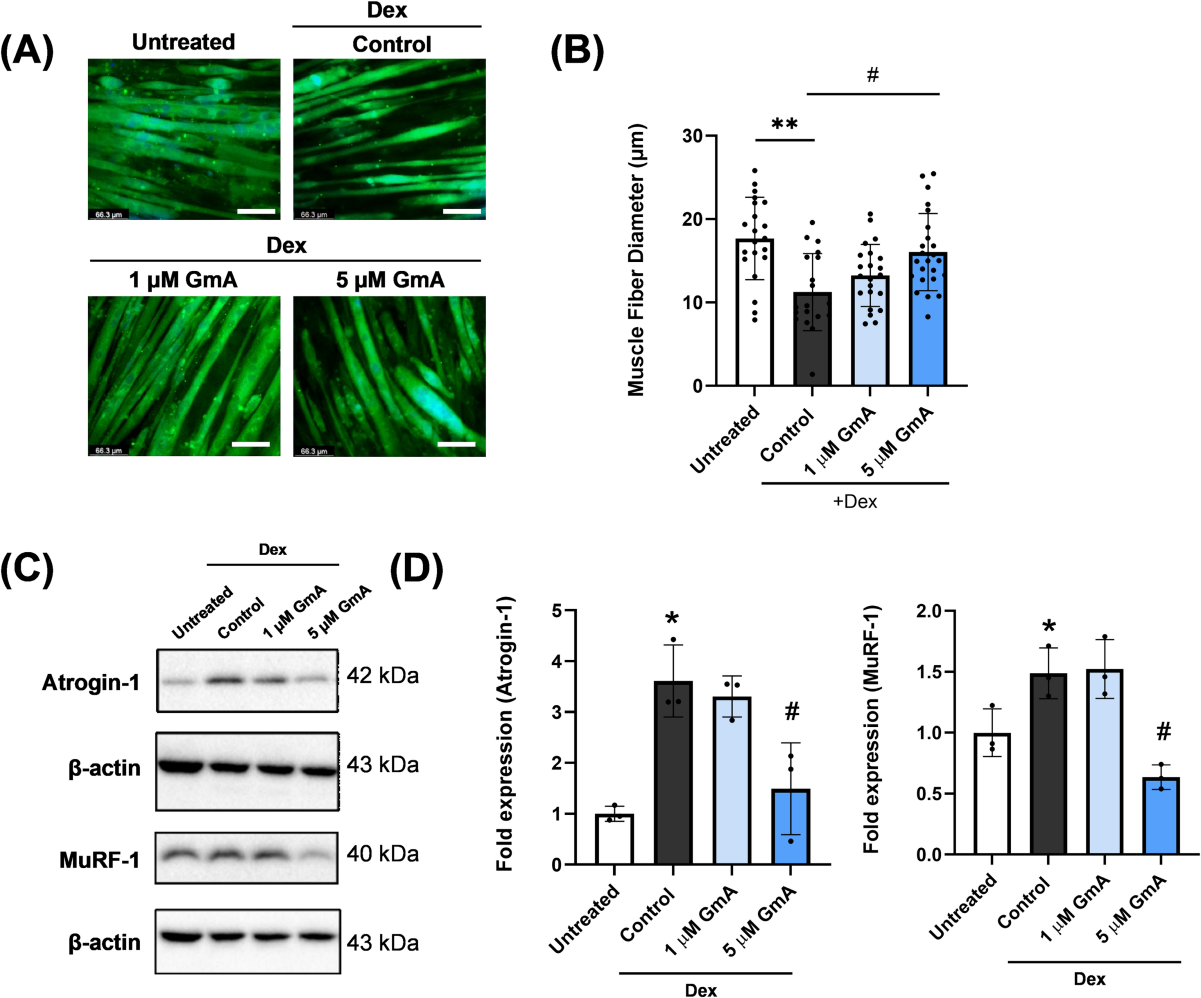
GmA inhibits Dex‐induced myotube atrophy. (A) Immunofluorescence staining for myosin heavy chain 2 in C2C12 myotubes treated with 10 μM dexamethasone (Dex) or Dex plus gymnemantoside A (GmA) at concentrations of 1 or 5 μM for 24 h. (B) Myotube mean diameter measurement (more than 30 myotubes were measured in each group). (C, D) Western blot analysis of MuRF‐1 and atrogin‐1 in C2C12 myotubes following 24 h of treatment with 10 μM Dex or 10 μM plus GmA (1 or 5 μM). Statistical significance is indicated as follows: *p < 0.05, **p < 0.01 compared with the untreated group. #*p* < 0.05 compared with the Dex‐treated myotubes (*n* = 3 per experimental group).

Differentiated C2C12 myotubes were treated with GmA in the presence of Dex (Figure 3A). Dex reduced the diameter to 12.17 μm (Figure 3B). GmA ameliorated this reduction in fibre diameter. The group treated with Dex and GmA also showed suppressed MuRF‐1 and atrogin‐1 protein levels (Figure 3C,D).

### 3.3 GmA Normalizes Autophagy in Dex‐Treated Myotubes

**FIGURE 4:**
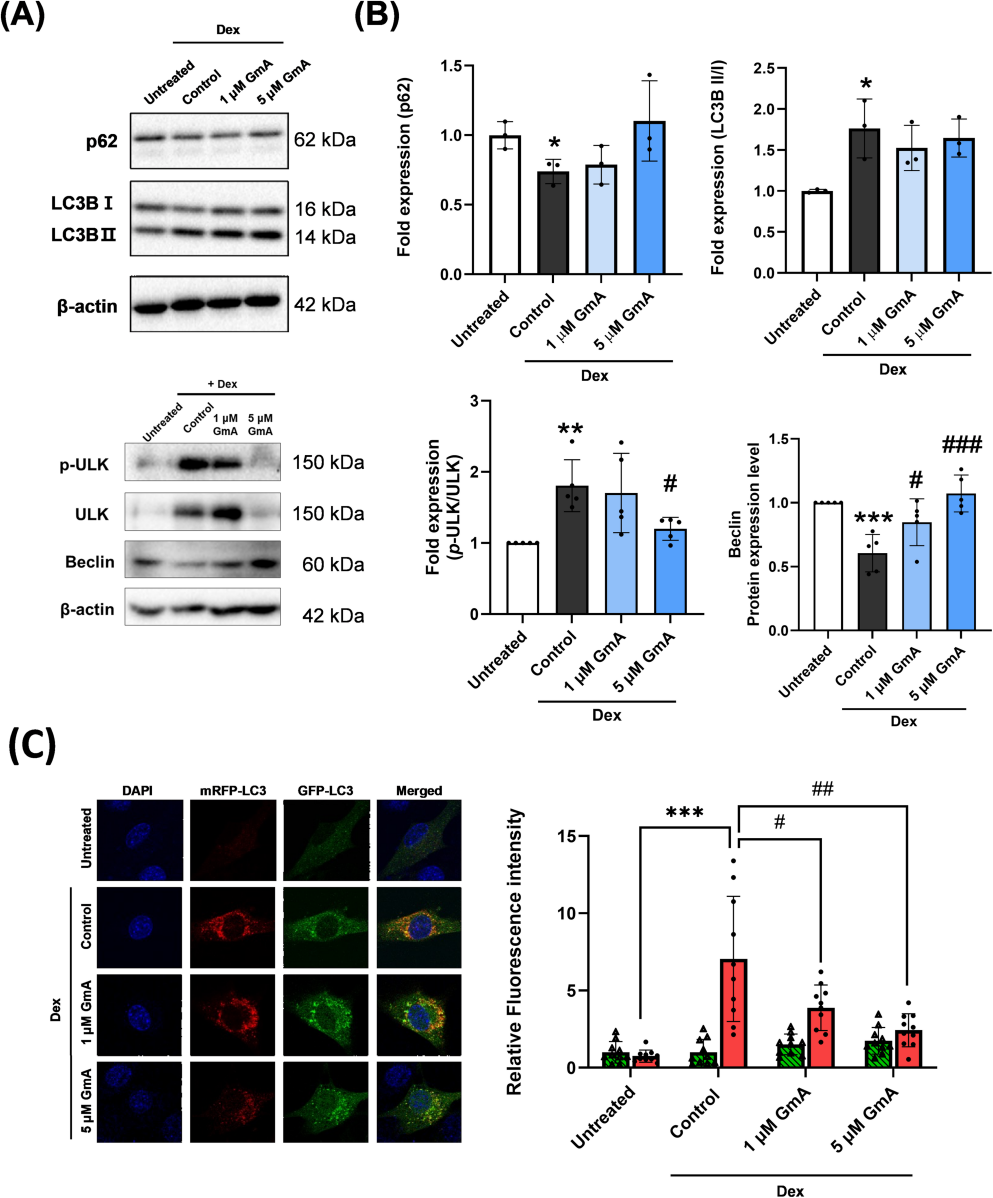
GmA normalizes autophagy in Dex‐treated myotubes. (A) Western blot analysis of the autophagy‐related proteins LC3‐II/LC3‐I, p62, phosphorylated ULK, total ULK and Beclin in C2C12 myotubes following 24 h of treatment with 10 μM dexamethasone (Dex) or 10 μM Dex plus gymnemantoside (GmA) at concentrations of 1 μM or 5 μM (n=3 per experimental group). (B) Densitometry analysis of LC3‐II/LC3‐I, p62, phosphorylated ULK, total ULK and Beclin. (C) Confocal microscopy images and fluorescence quantification of C2C12 muscle cells transfected with the mRFP‐GFP‐LC3 (ptf‐LC3) plasmid (n=10 per experimental group). Statistical significance is indicated as follows: *p < 0.05, *** *p* < 0.001 compared with the untreated. # *p* < 0.05, ## *p* < 0.01, compared with the Dex‐treated myotubes.

In the myotubes, Dex treatment increased the ratio of lipid modified microtubule‐associated protein 1A/1B light chain 3B (LC3B II) relative to the newly synthesized form (LC3B I), which is a marker of increased autophagy (Figure 4A). Dex treatment also decreased expression of the classical autophagy receptor p62, beclin 1 and increased the phosphorylation of Unc‐51 like autophagy activating kinase (ULK), which are markers of autophagy upregulation (Figure 4A,B) [11, 12]. GmA treatment reduced the mean values of the LC3B II/LC3B I ratio and increased p62 expression. However, these values did not reach statistical significance. In contrast, GmA treatment significantly decreased ULK phosphorylation and increased beclin‐1 expression (Figure 4A,B).

Myoblasts were transfected with the mRFP‐GFP‐LC3 plasmid, which can determine autophagy flux [13]. In the Dex treatment group, the fluorescence labeled LC3 punctate was observed as red fluorescence, which did not co‐localize with the GFP signal (Figure 4C). In contrast, the Dex plus GmA treatment group showed an increase of yellow co‐localized punctuates compared with Dex treatment alone, indicating that GmA treatment normalized autophagy. GmA treatment in GFP‐LC3 expressing human kidney cells produced only a modest reduction in autophagic puncta, indicating that GmA neither blocked autophagosome–lysosome fusion nor caused vesicle accumulation (Figure S4).

To gain further insight into the effects of GmA on skeletal muscle cells, murine myotubes were treated with GmA and analyzed with RNA‐Seq (Figure S5). Gene ontology (GO) analysis of biological processes showed that GmA suppressed autophagy‐related cell death and reduced the inhibition of cell division (Figure S5A). Cellular component analysis indicated that GmA treatment activated the muscle myosin complex (Figure S5B). Of note, the expression of BCL2 and adenovirus E1B 19‐kDa‐interacting protein 3 (Bnip3), a key inducer of autophagy, was reduced by GmA treatment (Figure S5C). Chromosomal changes related to cell division (such as segregation and condensation) were among the most enriched GO terms (Figure S5D). The upregulated genes included non‐SMC condensin I complex subunit D2 (Ncapd2), a known activator of mTOR and inhibitor of autophagy (Figure S5E) [14].

### 3.4 GmA Treatment Enhances the Akt/mTOR Signalling Pathway and Activates Protein Synthesis

**FIGURE 5:**
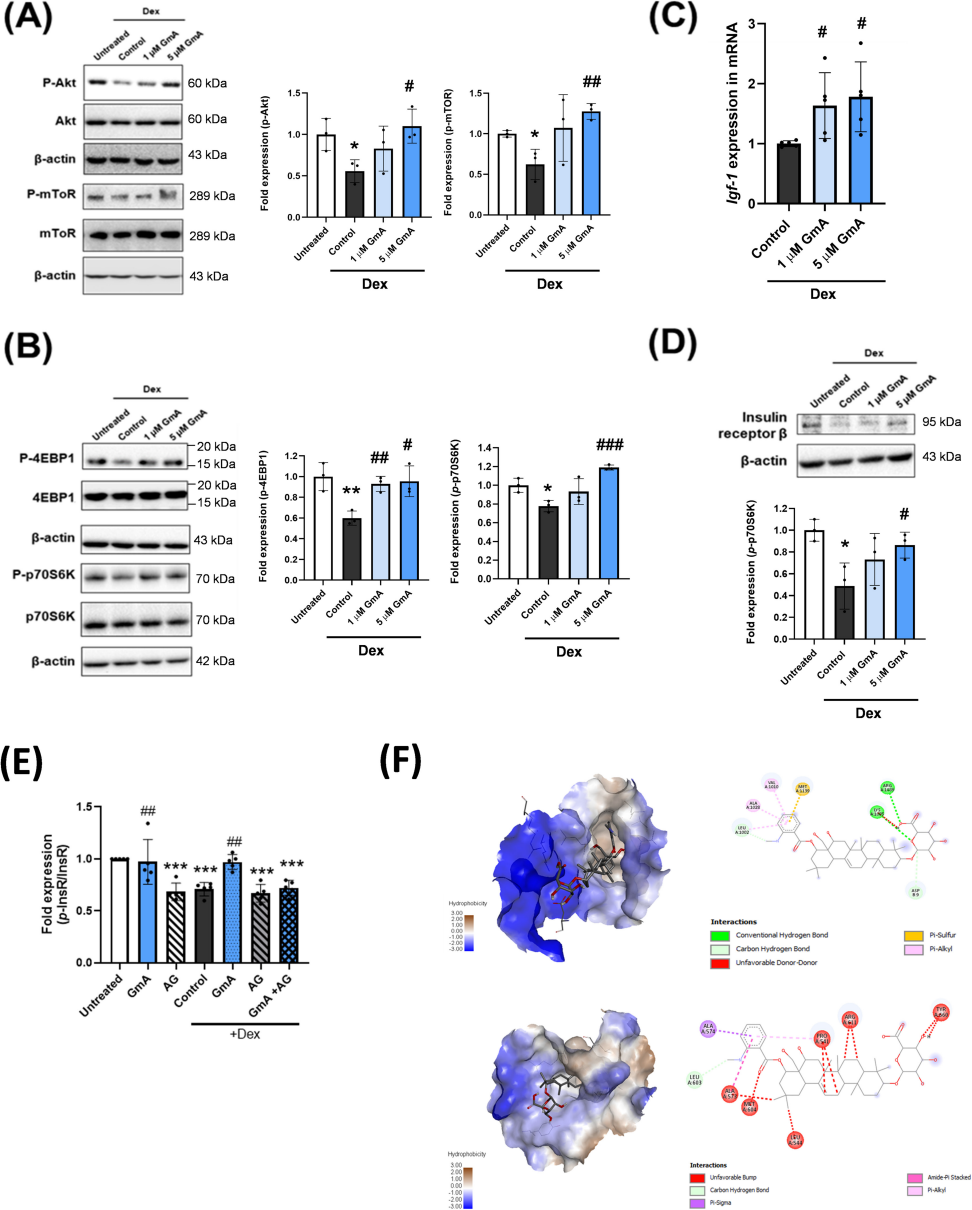
GmA activates insulin receptor signalling. (A) Western blot analysis of phosphorylated Akt, total Akt, phosphorylated mTOR, and total mTOR in C2C12 myotubes treated with 10 μM dexamethasone (Dex) for 24 h, followed by gymnemantoside A (GmA) at concentrations of 1 μM or 5 μM in combination with 10 μM Dex for an additional 24 h. β‐actin was used for normalization. (B) Western blot of phosphorylated 4EBP1, total 4EBP1, phosphorylated p70S6K, and total p70S6K in C2C12 myotubes treated with Dex and GmA as described in (A). (C) qPCR analysis of IGF‐1 expression in the treated myotubes. (D) Western blot of insulin receptor β in the C2C12 myotubes. (E) ELISA analysis of phosphorylated insulin receptor β with or without inhibitor AG1024 in C2C12 myotubes (*n* = 5). (F) Molecular docking binding analysis of the insulin receptor and GmA. (F) Molecular docking binding analysis of the glucocorticoid receptor and GmA. Statistical significance is denoted as follows: *p < 0.05, **p < 0.01, ***p < 0.001 compared with untreated. #*p* < 0.05, ##*p* < 0.01, ### *p* < 0.001 compared with the Dex‐treated myotubes (*n* = 3 per experimental group).

Dex treatment decreased Akt/mTOR pathway activity, as shown by reduced levels of phosphorylated Akt and mTOR. GmA treatment recovered the levels of phosphorylated Akt and mTOR in a dose‐dependent manner (Figure 5A). Furthermore, the phosphorylation levels of 4E‐binding protein 1 (4EBP1) and p70S6K kinase (p70S6K), two downstream activators of protein synthesis regulated by the Akt/mTOR pathway, were decreased by Dex treatment and rescued by GmA (Figure 5B).

### 3.5 GmA Regulates Insulin Receptor Signalling

The expression of insulin‐like growth factor 1 (IGF‐1), a ligand for the insulin receptor, was found to increase dose‐dependently after GmA treatment (Figure 5C). Additionally, GmA dose‐dependently recovered protein levels of the insulin receptor β chain (Figure 5D).

The InsR kinase inhibitor AG1024 eliminated the GmA‐mediated restoration of InsR and downstream PI3K phosphorylation, indicating that GmA requires InsR activity (Figure 5E and Figure S6A). By contrast, the PI3K inhibitor LY294002 did not lower the phospho‐PI3K signal (Figure S6A). However, it was found that LY294002 blocks conversion of PIP_2_ to PIP_3,_ but does not directly prevent phosphorylation of the PI3K regulatory subunit detected in our assay, meaning that PI3K status could not be reliably measured using this inhibitor (Figure S6A,B).

To further elucidate the interaction between GmA and the insulin receptor, molecular docking studies were conducted. It was observed that GmA exhibits a greater binding affinity for the insulin receptor compared with its standard ligand, phosphoaminophosphonic acid‐adenylate ester (ANP) (Figure 5F). The potential binding interaction between GmA and the Dex GR was also analyzed. The predicted binding affinity of GmA to the GR was 24.5 kcal/mol, in contrast to −12.5 kcal/mol for Dex (Figure 5F and Table S5).

### 3.6 Binding Kinetics of GmA to the Insulin‐Receptor TK Domain

Multi‐cycle SPR experiments confirmed that GmA binds the tyrosine‐kinase (TK) domain of the insulin receptor with markedly higher affinity than the reference ligand ANP (Figure S7 and Table S6).

Duplicate injections of GmA on the same HC1000 surface produced sensorgrams that fitted well to a 1:1 Langmuir model (χ^2^ = 4.74 ± 0.67 RU^2^), yielding *kₐ* = (4.15 ± 0.78) × 10^2^ ± 0.78 M^−1^ s^−1^ and *k*_*d*_ = (6.17 ± 1.63) × 10^−3^ s^−1^. These rate constants correspond to an equilibrium dissociation constant *K*_*D*_ = 14.8 ± 1.2 μM, which is about 25‐fold lower than that of the reference ligand ANP. SPR analysis of the GR ligand‐binding domain showed higher apparent affinity for GmA than for DEX, which is opposite to the docking rank order.

### 3.7 Gymnemantoside A Treatment Inhibits Skeletal Muscle Atrophy in the Dex Treatment Model

**FIGURE 6:**
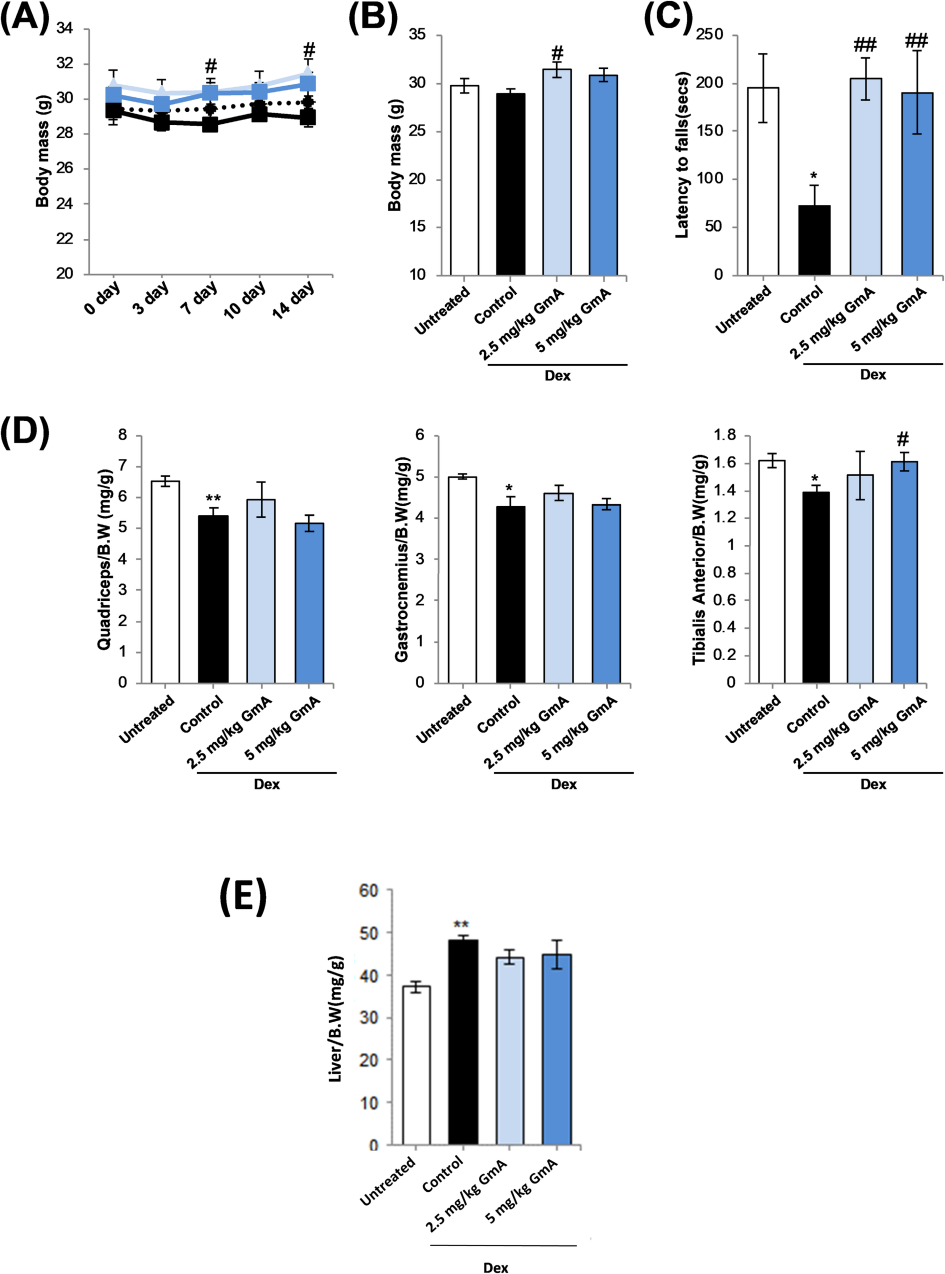
GmA prevents skeletal muscle atrophy in the Dex‐treatment model. (A, B) Body weight change in mice treated for 14 days as follows: (1) vehicle alone, (2) dexamethasone (Dex), (3) 2.5 mg/kg of gymnemantoside A (GmA), (4) Dex plus 5 mg/kg of GmA (*n* = 5). (C) Latency to fall in the rotarod test (*n* = 5). (D) Mass of the quadriceps, gastrocnemius, and TA muscles (*n* = 5). (E) Liver mass in the treated mice (*n* = 5). Statistical significance is expressed as follows: *p < 0.05, **p < 0.01 compared with the vehicle‐treated mice. #*p* < 0.05, ##*p* < 0.01 compared with the Dex‐treated mice.

The effects of GmA treatment were compared with Gi8. Dex treatment for 14 days reduced the mean body weight value, although it did not reach statistical significance (Figure 6A,B). GmA increased the mean body weight and reached statistical significance for the 2.5 mg/kg GmA treatment group. In contrast, Gi8 did not increase body weight (Figure S8A). Skeletal muscle performance was assessed using the rotor rod test. Latency to fall was significantly improved by treatment with GmA (Figure 6C). Gi8 treatment did not significantly increase muscle performance (Figure S8B). Liver mass was measured to gain insight into potential toxicity. Hepatomegaly was observed after the Dex treatment alone group. GmA treatment reduced the mean value of the liver mass, which lowered the statistical significance compared with untreated mice (Figure 6E).

It was observed that 2.5 mg/kg GmA treatment increased the mean mass values of the quadriceps, gastrocnemius and TA muscles, although this did not reach statistical significance; 3 mg/kg Gi8 treatment did not increase the quadriceps, gastrocnemius or TA mean mass value (Figure S8C). The 5 mg/kg GmA treatment dose significantly increased the mass of the TA muscle (Figure 6D).

### 3.8 GmA Treatment Protects Muscle Fibre Size in the Dex Treatment Model

**FIGURE 7:**
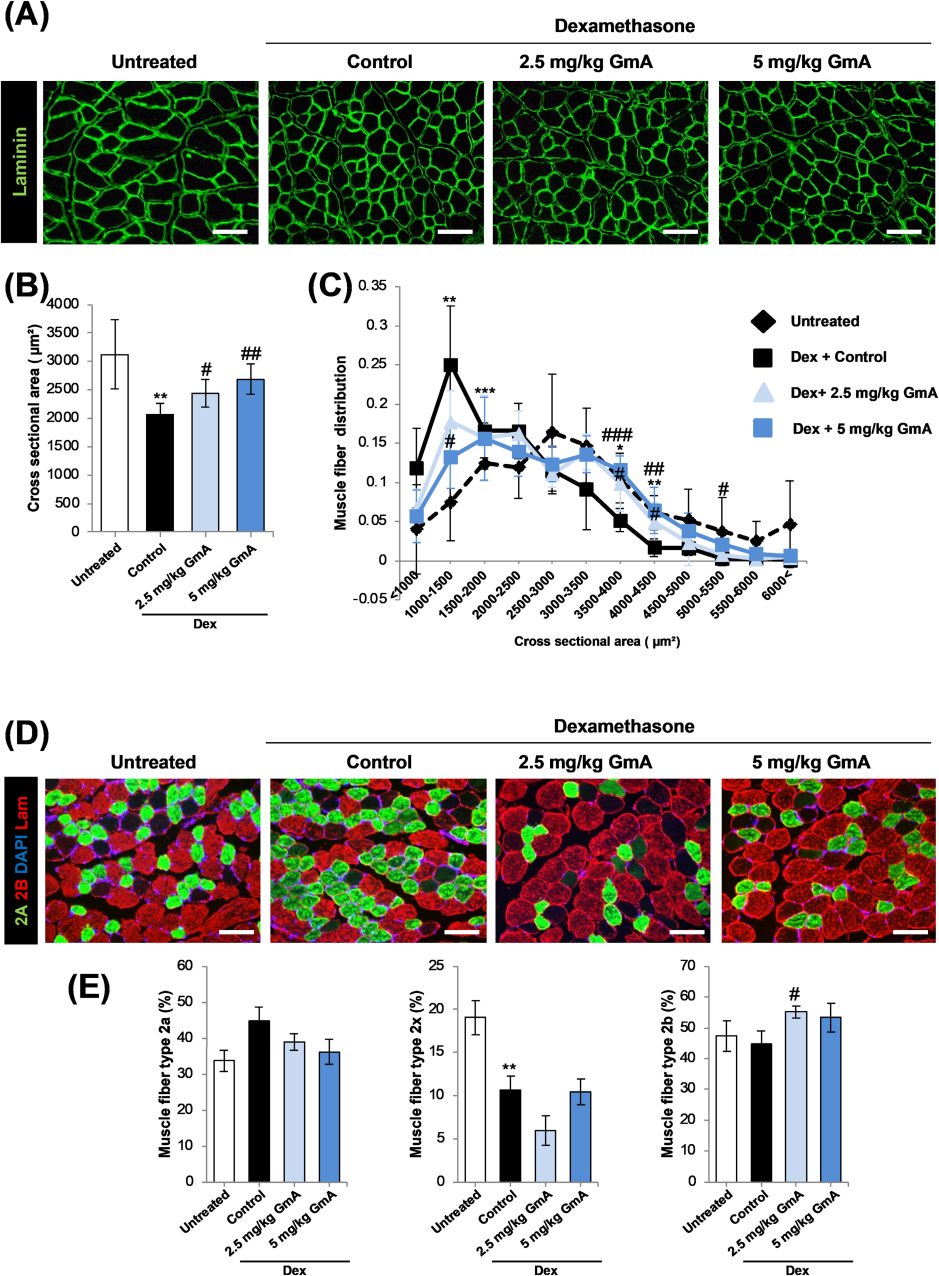
GmA increases myofibre cross sectional area and the proportion of fast type 2b myofibre**s.** (A) Representative anti‐laminin immunofluorescence staining of the TA muscle in mice treated for 14 d as follows: (1) vehicle alone, (2) dexamethasone (Dex), (3) Dex plus 2.5 mg/kg of gymnemantoside A (GmA), (4) Dex plus 5 mg/kg of GmA (scale bar = 150 μm) (B) Measurement of average myofibre cross‐sectional area (*n* = 200 myofibres measured). (C) Distribution of muscle fibre area. (D) Immunohistochemical analysis of TA muscle myofibre types 2a, 2b and 2x. (E) Quantification of muscle fibre types (*n* = 200 myofibres counted). Statistical significance is indicated as follows: *p < 0.05, **p < 0.01 compared with the vehicle‐treated mice. #*p* < 0.05, ##*p* < 0.01, ###*p* < 0.001 compared with the Dex‐treated mice.

Dex treatment for 14 days significantly reduced the mean muscle fibre cross‐sectional area (CSA). Co‐treatment with GmA at both tested doses inhibited this reduction in CSA (Figure 7A,B). Analysis of muscle fibre diameter distribution revealed that GmA treatment reduced the proportion of myofibres with a relatively small CSA (Figure 7C).

The distribution of three types of fast‐twitch muscle fibre types IIA (highly oxidative), IIB (highly oxidative/glycolytic) and IIX (highly glycolytic) was assessed in the gastrocnemius muscle (Figure 7D). Dex treatment alone produced an increase in the mean value of the proportion of fast myofibre type IIA (though the difference was not statistically significant), along with a significant reduction in the proportion of fast myofibre type IIX; 2.5 mg/kg GmA treatment significantly increased the proportion of fast myofibre type IIB (Figure 7E). The effect of GmA treatment on type I slow myofibres was assessed using qPCR (Figure S9A). GmA treatment increased the mean value of slow type myosin in the soleus muscle, but this did not reach statistical significance.

### 3.9 GmA Downregulates Atrogenes and Normalizes Autophagy‐Related Gene Expression Through IGF‐1 Upregulation in Dex‐Treated Skeletal Muscle

**FIGURE 8:**
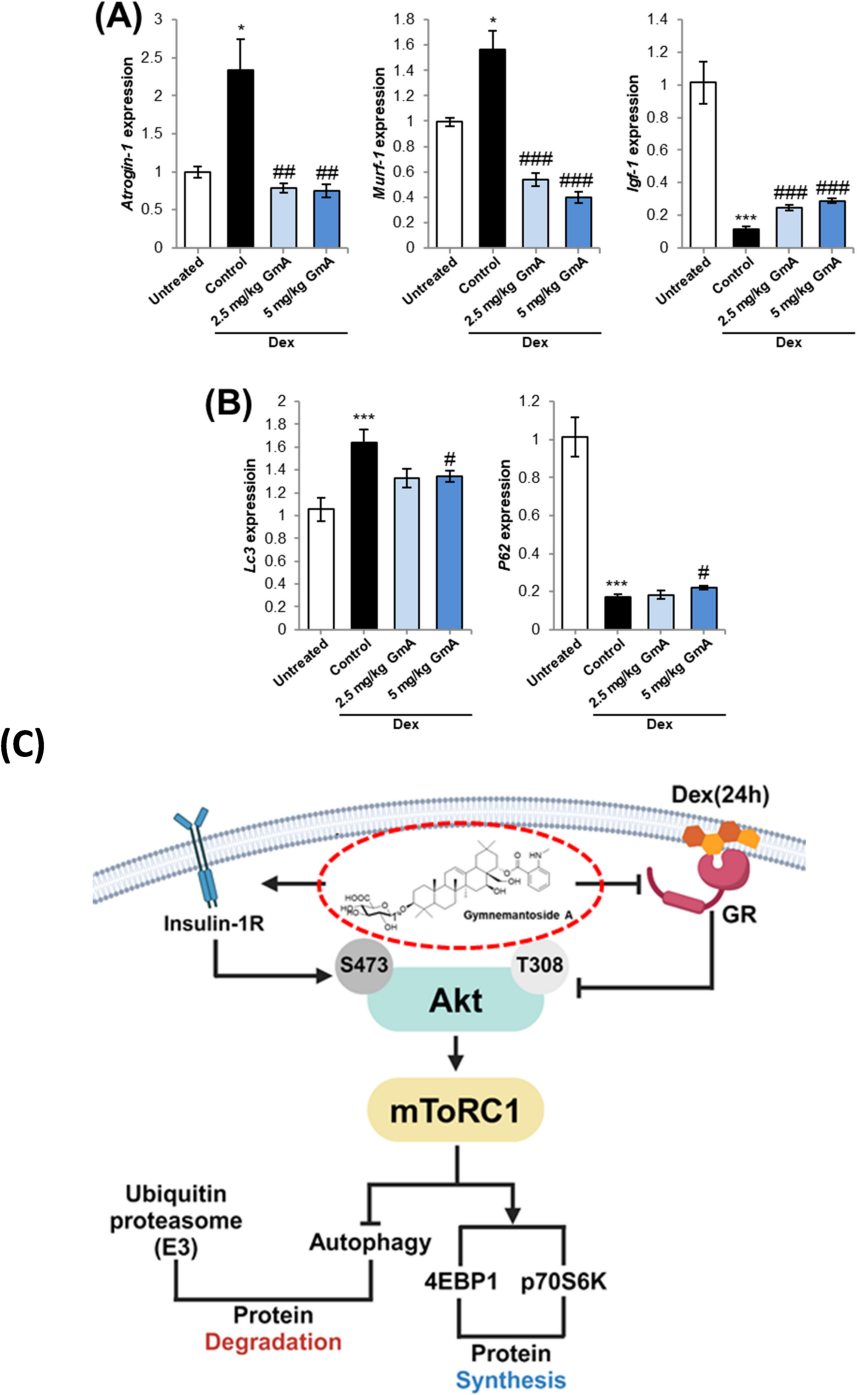
GmA reduces atrogene expression in the Dex‐treatment model. (A) Quantitative PCR (qPCR) analysis of the expression levels of atrogin‐1, Murf‐1 and IGF‐1, in the gastrocnemius muscle of mice treated with either vehicle alone, dexamethasone (Dex), Dex plus 2.5 mg/kg of gymnemantoside A (GmA) or 5 mg/kg of GmA A for 14 d (*n* = 5). (B) qPCR analysis of the expression levels of LC3 and P62 (*n* = 5). Statistical significance is indicated as follows: *p < 0.05, ***p < 0.001 compared with the vehicle‐treated mice. #*p* < 0.05, ##*p* < 0.01, ### *p* < 0.001 compared with the Dex‐treated mice. (C) Diagram illustrating the mechanism of action of GmA to prevent Dex‐induced skeletal muscle atrophy.

GmA treatment inhibited the upregulation of atrogin‐1 and MuRF‐1 in the Dex‐treated group (Figure 8A). GmA treatment also upregulated the expression of IGF‐1, which is a key inducer of skeletal muscle hypertrophy signalling (Figure 8A). GmA treatment inhibited the upregulation of LC3 in Dex‐treated skeletal muscle and produced a small, significant recovery of p62 expression at the 5 mg/kg dose (Figure 8B). Western blot analysis of the LC3B II/LC3B I ratio indicated that GmA treatment normalized autophagy (Figure S9B,C). PI3K phosphorylation, which is an upstream activator of AKT and mTOR signalling, was also increased by GmA treatment (Figure S9B,C).

### 3.10 Long‐Term GmA Treatment Does Not Significantly Affect Glucose Homeostasis or Liver Histology

To assess the effect of long‐term GmA treatment on metabolic parameters in normal mice, 9 weeks old male C57BL/6J mice received daily IP treatment with 5 mg/kg GmA for 4 weeks (Figure S10). Insulin tolerance test (ITT) analysis showed some significant effect of GmA treatment on glucose levels during the time course of the test, but no significant difference at the endpoint (Figure S10A). Moreover, the area under the curve (AUC) for the ITT showed no significant difference (Figure S10B). The glucose tolerance test (GTT) analysis showed significantly lower glucose levels at two time points in the GmA treated group but, similar to the ITT, there was no significant difference in glucose levels at the endpoint (Figure 10C). There was no significant difference in body weight between GmA treated and untreated mice (Figure 10D). Gastrocnemius muscle mass also showed no significant difference (Figure 10D). Exercise endurance, as measured by latency to fall in the rotorod test, showed a mean value almost two‐fold greater in the GmA treated mice, but this did not reach statistical significance (Figure 10E). Liver histology was assessed for potential hepatotoxicity after long‐term GmA treatment. There were no apparent features of liver damage, such as lipid infiltration, inflammation or fibrosis, in the GmA treated mice (Figure 10F).

### 3.11 ADME Analysis and Detection in Blood, Liver and Muscle Tissues

To gain insight into the potential pharmacokinetics of GmA, in silico ADME was carried out (Table S7). In addition, serum, liver and tibialis anterior muscle tissues were harvested from the 9‐week‐old C57BL/6J male mice after IP treatment with GmA for 4 weeks. A GmA standard compound was eluted at 5.8 min with the ion at *m/z* 784.4643. GmA and its metabolized derivative (resulting from the loss of glucuronic acid) were detected in both serum and liver (Figure S11). These two compounds exhibited a strong correlation in the molecular networking analysis. In liver, the ion at *m/z* 784.4620 was detected at the same retention time as the standard compound (5.8 min), along with the glucuronic acid‐detached form at *m/z* 608.4322. Similarly, in serum, *m/z* 784.4622 was observed at 5.8 min, matching the retention time of the standard, and its glucuronic acid‐detached form was detected at *m/z* 608.4346. In contrast to serum and liver, tissue samples showed the presence of a methylanthranilate‐detached form at *m/z* 651.1408, which exactly matched the calculated mass (Figure S11).

### 3.12 GmA Treatment Inhibits Autophagy in Immobilized Muscle and Reduces the Loss of Larger Myofibres

The effect of GmA on myofibre size and autophagy (as measured by the LC3B II/I ratio) was assessed in the hind limb immobilization (IMM) model. Immunohistochemical analysis of the gastrocnemius muscle showed no significant effect of GmA treatment on overall myofibre CSA (Figure S12A,B). Fibre area distribution showed that, compared with IMM alone, GmA treatment increased the % count of larger myofibres (greater than 3.0 × 10^3^ μm^2^), although this size population constituted a small proportion of the total myofibres (Figure S12C). IMM is known to increase autophagic flux in skeletal muscle. Western blot analysis of the LC3B II/I ratio showed that IMM increased this ratio in the gastrocnemius, indicating higher autophagy, and this was significantly reduced by GmA treatment (Figure S12D).

## 4 Discussion

The plant *G. inodorum* has been previously studied for its anti‐diabetic effects, but the specific, biologically active compound and its mechanism of action had not yet been described. It is noteworthy that during bio‐guided isolation from natural products, the effects of subfractions can sometimes decrease or even disappear, potentially due to the loss of synergistic effects among the components upon further isolation. However, in the case of *G. inodorum*, the isolated subfractions consistently maintained significant biological activity.

To experimentally test the docking prediction that GmA engages the insulin receptor kinase (IR‐TK) pocket, we carried out SPR binding assays with purified IR‐TK and GR ligand binding domain (GR‐LBD). SPR did confirm measurable direct binding of GmA to IR‐TK with micromolar affinity that exceeds the reference ligand ANP and aligns with the cellular restoration of InsR phosphorylation under Dex stress. When we examined GR‐LBD, apparent affinities reversed and GmA seemed to show stronger binding than Dex. We interpret the apparent reversal of GmA and Dex binding at the GR‐LBD as two technical considerations rather than nonspecific surface effects. First, the 25 Å docking grid captured the deep glucocorticoid cavity, but did not fully include the entrance corridor that opens between helices 6 and 7, when the receptor adopts ligand‐accessible states. Large ligands such as the triterpene glycoside GmA can extend through this gate in solution, so truncation of the gate penalizes the docking score. Secondly, GR‐LBD was analysed in SPR as a His tagged protein captured on a Ni‐NTA surface. In this orientation, the fraction of molecules presenting an unobstructed pocket to solution may be limited, which disproportionately reduces the apparent response to the compact ligand Dex while still allowing GmA to engage residues at the pocket rim and entrance. Consistent with limited functional relevance, GmA did not produce GR‐like responses in our cellular assays, so we view the GR SPR interaction as qualitative and place mechanistic weight on the insulin receptor engagement. These effects on the insulin/Akt/mTOR signalling suggest that it may be possible to test GmA as a potential treatment for other degenerative diseases linked to downregulation of this signalling system, such as heart failure and osteoarthritis [15, 16, 17].

RNA‐Seq was used to further investigate the effect of GmA on muscle cells treated with Dex. It was observed that GmA treatment suppressed autophagic cell death, which is consistent with the results obtained with the mRFP‐GFP‐LC3 (ptf‐LC3) probe of autophagy levels in myotubes. Insulin signalling is known to inhibit autophagy via the PI3K/AKT/mTOR pathway, which in turn inhibits the dysregulated accumulation of autophagosomes and autophagy‐associated cell death [18, 19]. Thus, the interaction of GmA with the insulin receptor and downstream activation of mTOR may be the mechanism responsible for the suppression of autophagy‐associated cell death. Gene analysis indicated that GmA reduced the expression of Bnip3 in the myotubes. Bnip3 has been shown to be a potent inducer of autophagy in multiple cell types [20]. In addition, Bnip3 levels can be downregulated by mTOR activity [21].

RNA‐Seq also revealed that GmA treatment regulated chromosome segregation and promoted cell division. This included upregulation of Ncapd2, which increases chromosome condensation. Ncapd2 has also been shown to increase mTOR activity and suppress key autophagy regulators, such as beclin‐1 and autophagy protein 5 (ATG5) [14]. The effect of GmA on gene expression related to cell division may explain the increase in MTT assay signal observed in GmA‐treated myotube cultures, due to enhanced proliferation in the residual undifferentiated myoblasts.

Cellular component analysis also showed that GmA treatment activated the muscle myosin complex, which is composed of two heavy chains, two myosin essential light chains, and two myosin regulatory light chains [22]. This may contribute to the observed increase in muscle mass following GmA treatment.

The results presented in this study have shown that GmA is effective at preventing skeletal muscle atrophy in the Dex treatment model, which is a widely used model of muscle wasting. When tested in the IMM model, GmA did not prevent the overall reduction in myofibre CSA, although autophagy was reduced and there was an increase in the proportion of larger myofibres. This may be due to the IMM model being a more ‘severe’ form of muscle atrophy that is induced by a complete removal of the nerve stimulus to the entire muscle. It can be noted that glucocorticoid‐induced muscle atrophy is the most common form of drug‐induced atrophy [23], meaning there would be sufficient patient need to justify the further pre‐clinical development of GmA, based on the results of the Dex model. In addition, further studies should focus on testing GmA in other models of muscle atrophy, such as cancer cachexia and chronic kidney disease, especially when autophagy is the main process driving muscle loss.

In summary, GmA is a bioactive compound isolated from *G*. *inodorum* possessing a previously undescribed structure. Mechanistically, GmA treatment was found to directly activate insulin receptor signalling and the downstream Akt/mTOR pathway to counter muscle atrophy (Figure 8C). This anti‐atrophy effect can be attributed to an increase in the proportion of type IIb fast glycolytic myofibres that are known to be lost in sarcopenia, along with a general improvement in myofibre CSA. This research highlights a novel compound with promising properties for the development of a natural products‐based therapeutic for muscle wasting in patients.

Additional Discussion is provided in the Supplementary Information.

## Conflicts of Interest

E.‐J.P., H.‐J.K., D.‐W.J., D.R.W. and W.K.O. are named co‐inventors of a pending provisional patent application based in part on the research reported in this paper. The rest of the authors declare no conflicts of interest.

## Supporting information


**Supporting Information S1:** Additional supporting information may be found online in the Supporting Information section at the end of the article.
**Figure S1:** (A) 1H NMR data of GmA (compound 1) (400 MHz, Pyridine–d5), (B) 13C NMR data of compound 1 (100 MHz, Pyridine–d5) (C) HSQC NMR data of compound 1. Compound 1 was obtained as a white, amorphous powder, exhibiting a specific optical rotation of ${\left[\alpha \right]}_{D}^{25}$ +12.7 (c 0.1, MeOH). Its molecular formula, C_44_H_65_NO_11_, was deduced from the HRESIMS ion peak at m/z 784.4612 [M + H]^+^, (calcd for C_44_H_65_NO_11_, 784.4636), indicating 12° of unsaturation. The IR spectrum showed bands at 3365, 1608 and 1514 cm^−1^, characteristic of NH stretching, olefinic and NH bending absorbance. The ^1^H NMR spectrum revealed signals for four aromatic protons (δ_H_ 8.47, dd, J = 8.1, 1.5 Hz, H‐7′; 7.40, ddd, J = 8.7, 7.0, 1.6 Hz, H‐5′; 6.66, dd, J = 8.0, 5.7 Hz, H‐4′, H‐6′), one olefinic proton (δ_H_ 5.36, d, J = 4.0 Hz, H‐12), an anomeric proton (δ_H_ 5.03 (d, J = 7.7 Hz, H‐1″), one nitrogenated methyl group (δ_H_ 2.80, s, H_3_–8′) and seven methyl groups (δ_C_ 1.47, 1.31, 1.28, 1.01 (6H), 0.95 and 0.81). The ^13^C NMR spectrum displayed resonances for two carbonyl carbons (δ_C_ 170.7 and 168.9), six aromatic carbons (δ_C_ 152.3, 135.1, 133.4, 115.1, 112.6 and 111.9), two olefinic carbons (δ_C_ 143.0 and 124.5), and one anomeric carbon (δ_C_ 107.2), suggesting the structure of compound 1 as a pentacyclic triterpene aglycone with one glucuronic acid and an N‐methyl anthranilate (Mant) moiety. (D) HMBC spectrum data of compound 1. The HMBC correlations from the nitrogenated methyl signal at δ_H_ 2.80 to an aromatic carbon (δ_C_ 152.3, C‐3′) and from the aromatic proton signal at δ_H_ 8.47 to a carbonyl carbon (δ_C_ 168.9, C‐1′) suggested the presence of an anthranilate group. The position of this group was confirmed by the HMBC correlation from H‐22 (δ_H_ 6.36, dd, J = 10.5, 5.4 Hz) to C‐1′. COSY correlations for H_2_–15 (δ_H_ 2.18, 1.74)/H‐16 (δ_H_ 5.11) and H‐21 (δ_H_ 2.00)/H‐22 (δ_H_ 6.36) indicated that C‐16 and C‐22 were oxygenated. The oxygenated methylene group at C‐17 was confirmed based on an HMBC correlation from H_2_–28 (δ_H_ 4.51, 4.07) to C‐17 (δ_C_ 40.0). The presence of a glucuronic acid moiety was deduced from the HMBC correlation between H‐5″ of glucuronic acid (GlcA) (δ_H_ 4.69) and C‐6″ (δ_C_ 170.7). In the HMBC spectrum, the anomeric proton of the glucuronic acid unit showed a correlation with C‐3 (δ_C_ 89.7) of the aglycone. The α‐orientation of the N‐methyl anthranilate moiety at C‐22 was inferred from the NOESY correlation between H‐18 (δ_H_ 3.03, dd, J = 13.9, 5.8 Hz) and H‐22 (δ_H_ 6.36). The NMR data observed for compound 1 were similar to those of (3β,16β,22α)‐22‐(N‐methylanthraniloxy)‐16,28‐dihydroxyolean‐12‐en‐3‐yl‐3‐O‐β‐D‐glucopyranosyl‐β‐D‐glucopyranosiduronic acid [4], except for the absence of a glucose unit. Therefore, compound 1 was identified as (3β,16β,22α)‐22‐(N‐methylanthraniloxy)‐16,28‐dihydroxyolean‐12‐en‐3‐yl‐β‐D‐glucopyranosiduronic acid and named as gymnamantoside A (GmA). (E) COSY spectrum data of compound 1. (F) NOESY spectrum data of compound 1. Gymnamantoside A (GmA) (1): White amorphous powder; ${\left[\alpha \right]}_{D}^{25}$ + 12.7 (*c* 0.1, MeOH); UV (MeOH) *λ*_max_ (log *ε*) 223 (1.72), 257 (1.24), 357 (1.10); IR (KBr) *ν*_max_ 3365, 2941, 2873, 356, 2312, 1751, 1672, 1608, 1514, 1427, 1362, 1240, 1160, 1077, 1027, 954, 752 cm^−1^; ^13^C and ^1^H NMR data, see Supplementary Table S3; HRESIMS *m/z* 784.4612 [M + H]^+^, (calcd for C_44_H_65_NO_11_, 784.4636).
**Figure S2:** Calibration curve and representative UV‐HPLC chromatograms for the quantification of gymnemantoside A (GmA). (A) Calibration curve constructed from six standard solutions of GmA (0.03125–1.000 mg/mL); linear regression yielded the equation *y* = 1790.8 *x* + 9.6687 (*r* ^2^ = 0.9998). (B) UV chromatograms at 254 nm for (1) GmA standard, (2) Bu1, (3) Bu2, (4) Bu3 and (5) Bu4 subfractions. The shaded band indicates the retention window of GmA (23.1 min). Among the subfractions, Bu3 exhibits the most prominent GmA peak.
**Figure S3:** Chemical structures of gymnemantoside A (GI12) and GI8. Chemical differences are shown using coloured ovals.
**Figure S4:** Gymnemantoside A attenuates dexamethasone‐induced autophagy in HEK293 reporter cells. (A) GFP‐LC3 reporter. Representative fluorescence images showing GFP‐LC3 puncta after 24 h of treatment: Untreated (basal); Dexamethasone (Dex, 10 μM); Dex + Gymnemantoside A (GmA, 5 μM), and Chloroquine (CQ, 25 μM; a late‐stage autophagy inhibitor that induces marked GFP‐LC3 vesicle accumulation). Nuclei were counterstained with DAPI (blue). Images were acquired using an Axio Observer inverted fluorescence microscope. (B) Tandem mRFP‐GFP‐LC3 flux reporter. HEK293 cells infected with an adenoviral mRFP‐GFP‐LC3 construct were treated as indicated (Untreated; Dex; Dex + GmA; CQ; rapamycin). GFP (green) is acid‐labile and quenched in autolysosomes, while mRFP (red) is retained. Therefore, yellow puncta (GFP + RFP) indicate autophagosomes, and red‐only puncta indicate acidified autolysosomes. Dex increased autophagic structures; co‐treatment with GmA attenuated this effect; CQ led to the accumulation of undegraded autophagosomes, whereas rapamycin induced autophagy.
**Figure S5:** RNA‐Seq gene ontology (GO) analysis of (A) Biological processes and (B) Cellular component in C2C12 murine myotubes treated with GmA. (C) Expression of the key inducer of autophagy, Bnip3. (D) Most enriched GO terms. (E) Gene expression changes related to chromosome segregation during mitosis. The expression of Ncapd2, an activator of mTOR and inhibitor of autophagy, is indicated using black arrows.
**Figure S6:** Inhibitor analysis of PI3K by Western blotting. (A) Representative immunoblots showing phospho‐PI3K p85 (Tyr458), total PI3K p85 and β‐actin in C2C12 myotubes following 24 h of treatment and a 15‐min terminal inhibitor pulse, as described in the Method section. (B) Representative immunoblots for phospho‐PI3K p85 (Tyr458) and total PI3K p85 in cells subjected to the same terminal pulse protocol, but without dexamethasone treatment.
**Figure S7:** SPR confirmation of ligand binding to the insulin‐receptor TK domain. (A) Gymnemantoside A (GmA). Left, steady‐state binding plot of equilibrium responses versus analyte concentration (0.312–20 μM) fitted with a 1:1 isotherm (solid line), yielding a dissociation constant (*K*_*D*_) of 14.75 ± 1.20 μM (*n* = 2 technical duplicates). Right, overlaid sensorgrams corresponding to the same concentration series, with the global fit shown black. (B) Reference ligand ANP. Left, steady‐state binding plot for ANP (62.5–1000 μM), fitted with a 1:1 binding model (*K*_*D*_ **=** 376.00 ± 2.83 μM). Right, multi‐cycle sensorgrams and global fit. (C) GmA binding to GR ligand‐binding domain (GR‐LBD). Left, steady‐state binding plot GmA (0.312–10 μM), showing a *K*_*D*_ = 3.97 ± 0.26 μM. Right, corresponding multi‐cycle sensorgrams and global fit. (D) Dexamethasone. Left, steady‐state plot for dexamethasone (25–400 μM), fitted to a 1:1 model with a *K*_*D*_ = 199.50 ± 12.00 μM. Right, sensorgrams and global fit. In all panels, arrows indicate the analyte association (injection) and dissociation phases; colours present increasing analyte concentrations.
**Figure S8:** (A) Body weight in mice treated with vehicle alone, Dex, Dex plus 1 mg/kg of GI8 or 3 mg/kg of GI8 for 14 d (*n* = 5). (B) Latency to fall off the rotarod (*n* = 5). (C) Quadriceps, gastrocnemius and tibialis anterior muscle mass (*n* = 5). * = *p* < 0.05 compared with vehicle alone.
**Figure S9:** (A) qPCR analysis of type 1 slow myosin (Myh7) expression in the gastrocnemius and soleus muscles of mice treated with vehicle alone, Dex or Dex plus 2.5 mg/kg of GmA for 14 d. (B) Western blot analysis of PI3K, phosphorylated PI3K (P‐PI3K) and LC3B II/I in the gastrocnemius muscle. (C) Quantification of the LC3B II/I ratio and PI3K phosphorylation. * = *p* < 0.05 and ** *= p* < 0.01 compared with vehicle.
**Figure S10:** (A‐B) Insulin tolerance test (ITT) and area under the curve (AUC) for 13‐weeks‐old male C57BL/6J mice that received daily IP treatment with 5 mg/kg GmA for the previous 4 weeks. (C) Glucose tolerance test (GTT). * = *p* < 0.05 and ** *= p* < 0.01 compared with untreated. (D) Body weight and gastrocnemius muscle weight. (E) Latency to fall in the rotarod test. (F) Representative liver H&E histology.
**Figure S11:** ADME analysis of GmA in 14‐weeks‐old mice after 4 weeks of daily IP treatment with 5 mg/kg GmA or vehicle alone. GmA and its metabolized derivative (a glucuronic acid‐detached form) were detected in both the serum and liver tissue. In contrast, skeletal muscle samples showed the presence of a methylanthranilate‐detached form of GmA.
**Figure S12:** Effect of GmA on myofibre size and autophagy in a hind limb immobilization (IMM) model. (A) Representative laminin and DAPI staining of the gastrocnemius muscle. (B) Cross sectional area (CSA) measurement. (C) Myofibre size distribution of larger fibres (> 2.5x10^3^ (μm^2^) (at least 80 myofibres were measured in each photo (*n* = 4). (D) Western blot analysis of LC3B and GAPDH. (E) Quantification of the LC3B II/I ratio (* = *p* < 0.05).

**Table S1:** Primer sequences used in this study.
**Table S2:** jcsm70118‐sup‐0002‐Supplementary_Tables.pptx. ^1^H and ^13^C NMR data of gymnemantoside A isolated from *G. inodorum*.
**Table S3:** Calibration and validation parameters for the HPLC‐UV quantification of gymnemantoside A
**Table S4:** Concentration of gymnemantoside A detected in subfractions Bu1–Bu4 by HPLC‐UV analysis
**Table S5:** Molecular docking analysis result between compound and ligands.
**Table S6:** Surface plasmon resonance (SPR) kinetic (*k*_*a*_, *k*_*d*_) and equilibrium (*K*_*A*_, *K*_*D*_) binding parameters for gymnemantoside A (GmA) and reference ligands interacting with the insulin receptor (IR) tyrosine kinase domain and the glucocorticoid receptor (GR) ligand‐binding domain (LBD). R_max_, maximum response (RU); χ^2^, fit quality. Values are mean ± SD
**Table S7:** Predicted ADME parameters of compound GMA obtained through computational analysis.

**Data S1:** Supplementary Information.
