# Supplementary material for: Gymnemantoside A Ameliorates Steroid‐Induced Skeletal Muscle Atrophy via Bridging Glucocorticoid and Insulin Receptor Signalling
Source: J Cachexia Sarcopenia Muscle. 2025 Nov 25;16(6):e70118. doi: 10.1002/jcsm.70118 (PMC12646868; doi:10.1002/jcsm.70118)
Supplement: Supplementary file 1 — Supporting Information S1: Additional supporting information may be found online in the Supporting Information section at the end of the article. Figure S1: (A) 1H NMR data of GmA (compound 1) (400 MHz, Pyridine–d5), (B) 13C NMR data of compound 1 (100 MHz, Pyridine–d5) (C) HSQC NMR data of compound 1. Compound 1 was obtained as a white, amorphous powder, exhibiting a specific optical rotation of αD25 +12.7 (c 0.1, MeOH). Its molecular formula, C44H65NO11, was deduced from the HRESIMS ion peak at m/z 784.4612 [M + H]+, (calcd for C44H65NO11, 784.4636), indicating 12° of unsaturation. The IR spectrum showed bands at 3365, 1608 and 1514 cm−1, characteristic of NH stretching, olefinic and NH bending absorbance. The 1H NMR spectrum revealed signals for four aromatic protons (δH 8.47, dd, J = 8.1, 1.5 Hz, H‐7′; 7.40, ddd, J = 8.7, 7.0, 1.6 Hz, H‐5′; 6.66, dd, J = 8.0, 5.7 Hz, H‐4′, H‐6′), one olefinic proton (δH 5.36, d, J = 4.0 Hz, H‐12), an anomeric proton (δH 5.03 (d, J = 7.7 Hz, H‐1″), one nitrogenated methyl group (δH 2.80, s, H3–8′) and seven methyl groups (δC 1.47, 1.31, 1.28, 1.01 (6H), 0.95 and 0.81). The 13C NMR spectrum displayed resonances for two carbonyl carbons (δC 170.7 and 168.9), six aromatic carbons (δC 152.3, 135.1, 133.4, 115.1, 112.6 and 111.9), two olefinic carbons (δC 143.0 and 124.5), and one anomeric carbon (δC 107.2), suggesting the structure of compound 1 as a pentacyclic triterpene aglycone with one glucuronic acid and an N‐methyl anthranilate (Mant) moiety. (D) HMBC spectrum data of compound 1. The HMBC correlations from the nitrogenated methyl signal at δH 2.80 to an aromatic carbon (δC 152.3, C‐3′) and from the aromatic proton signal at δH 8.47 to a carbonyl carbon (δC 168.9, C‐1′) suggested the presence of an anthranilate group. The position of this group was confirmed by the HMBC correlation from H‐22 (δH 6.36, dd, J = 10.5, 5.4 Hz) to C‐1′. COSY correlations for H2–15 (δH 2.18, 1.74)/H‐16 (δH 5.11) and H‐21 (δH 2.00)/H‐22 (δH 6.36) indi [file JCSM-16-e70118-s001.pptx]

## Slide 1
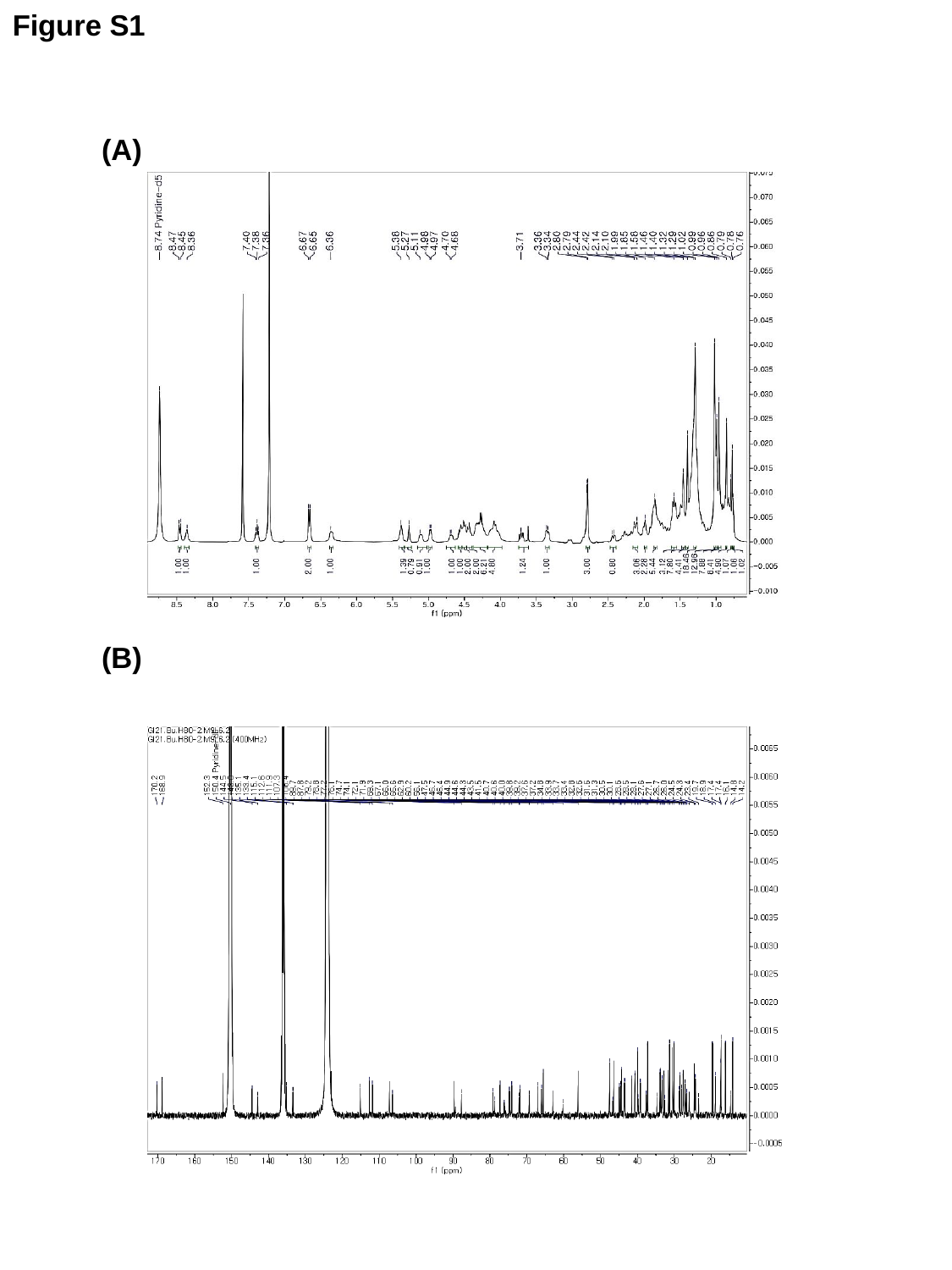

Figure S1
(A)
(B)

## Slide 2
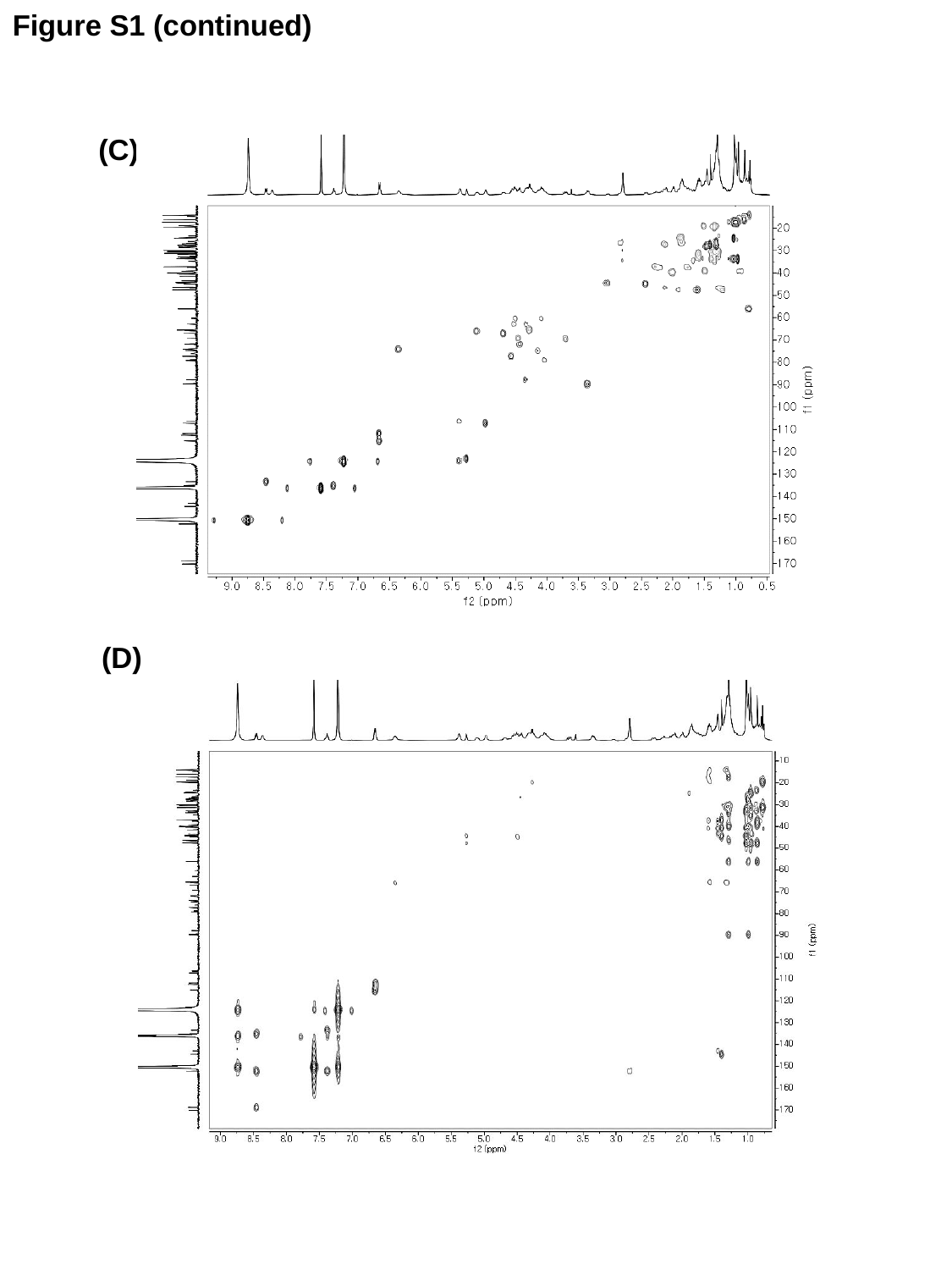

Figure S1 (continued)
(C)
(D)

## Slide 3
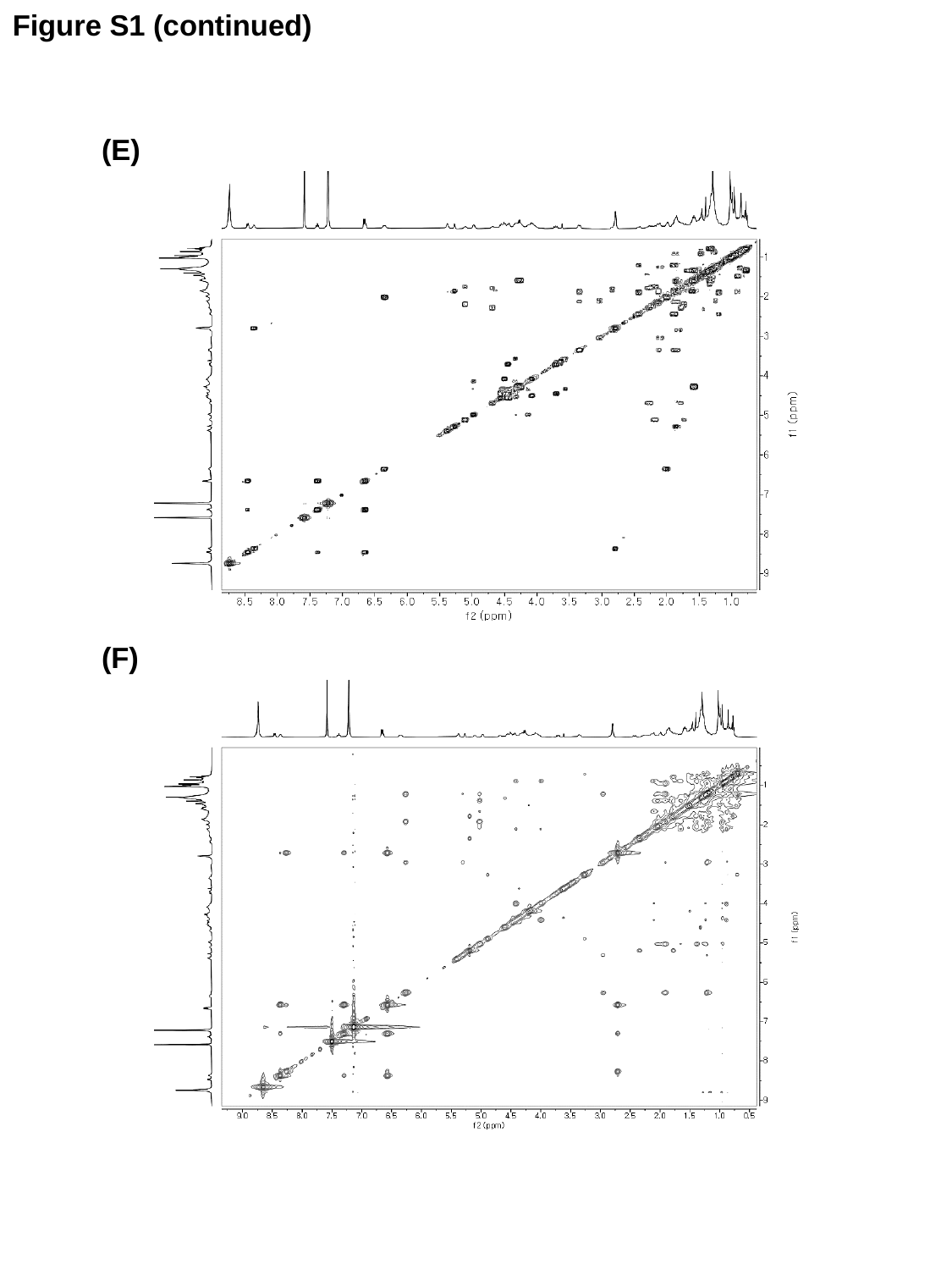

Figure S1 (continued)
(E)
(F)

## Slide 4
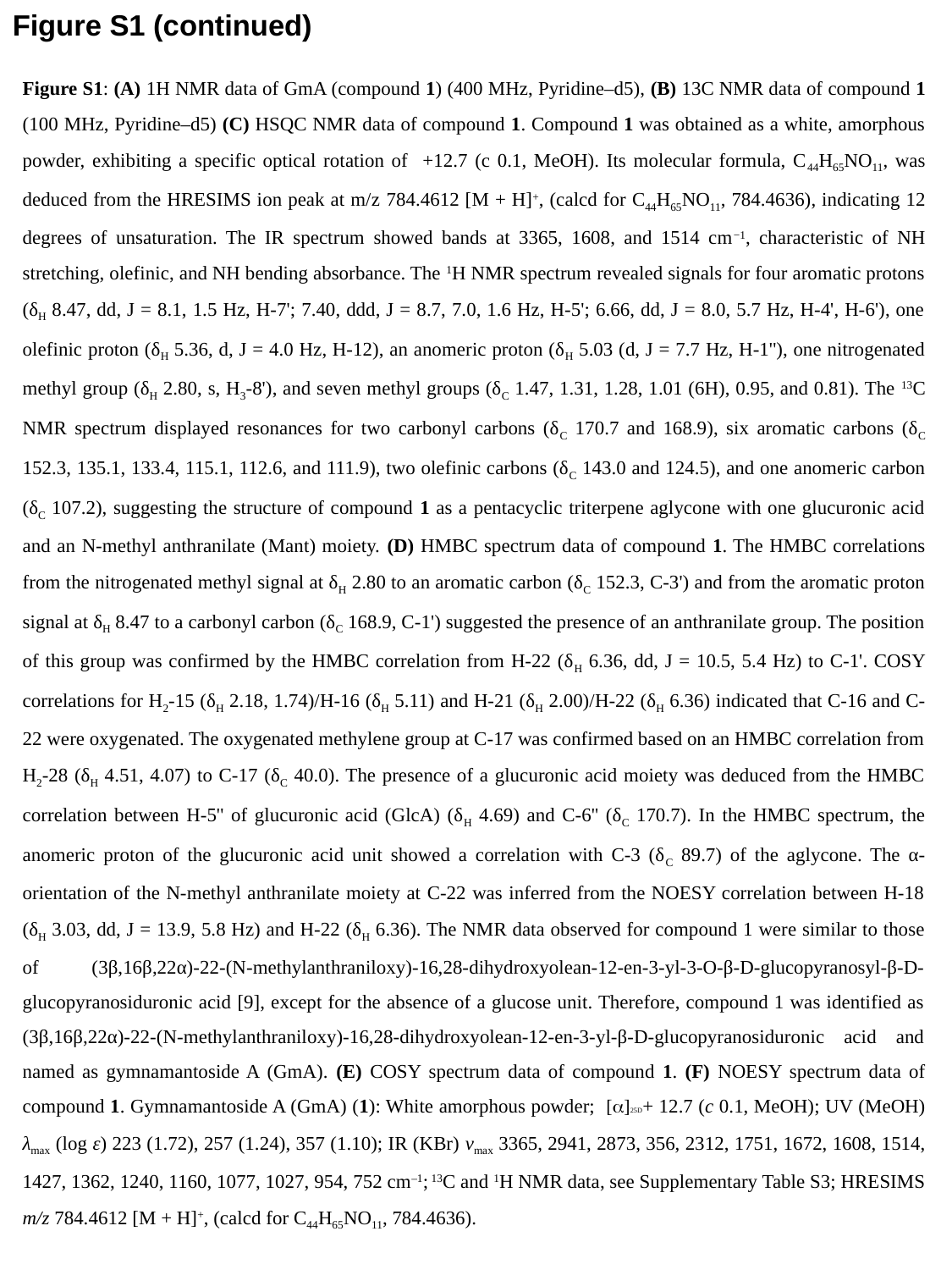

Figure S1 (continued)

## Slide 5
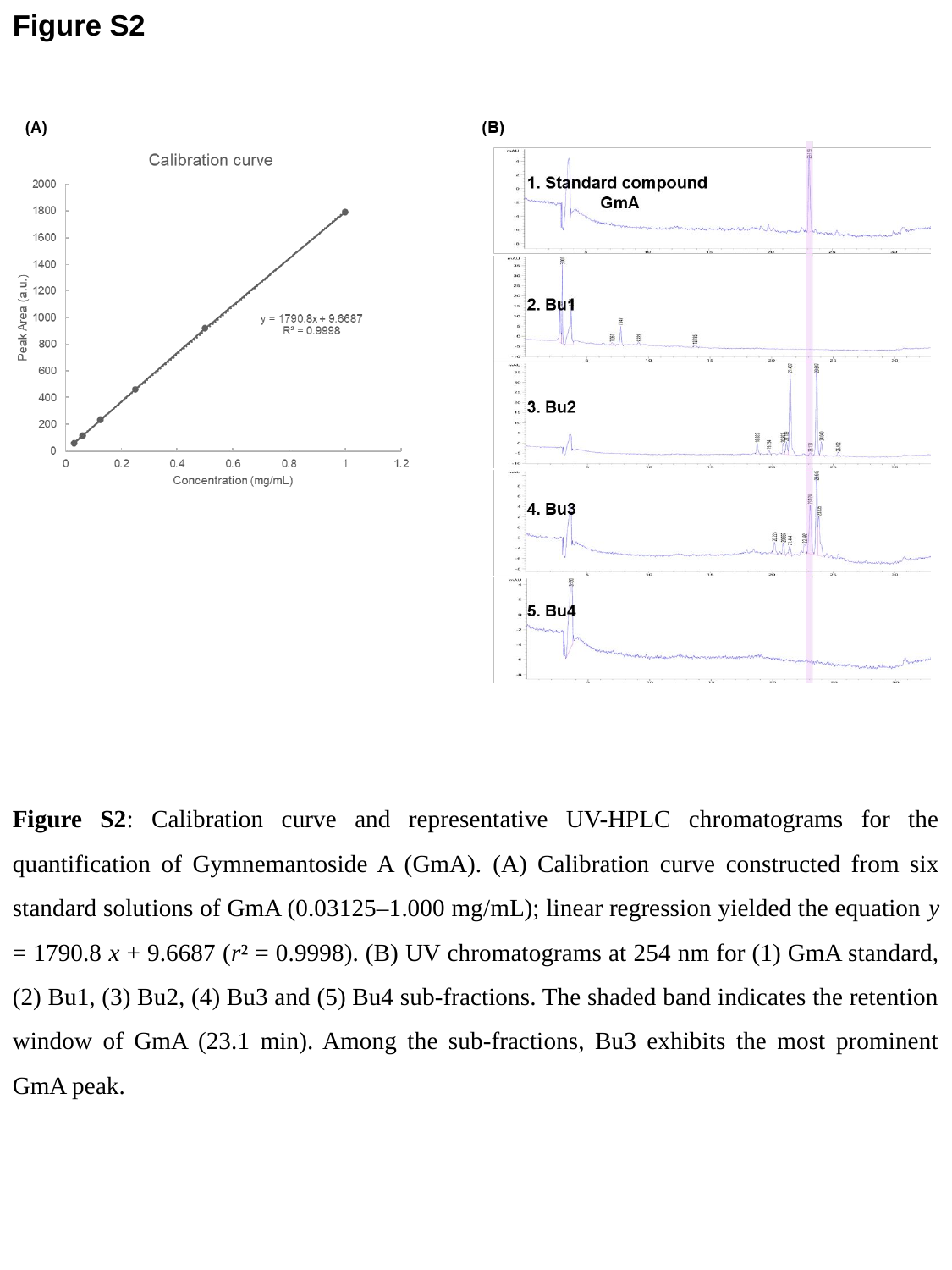

Figure S2
Figure S2: Calibration curve and representative UV-HPLC chromatograms for the quantification of Gymnemantoside A (GmA). (A) Calibration curve constructed from six standard solutions of GmA (0.03125–1.000 mg/mL); linear regression yielded the equation y = 1790.8 x + 9.6687 (r² = 0.9998). (B) UV chromatograms at 254 nm for (1) GmA standard, (2) Bu1, (3) Bu2, (4) Bu3 and (5) Bu4 sub-fractions. The shaded band indicates the retention window of GmA (23.1 min). Among the sub-fractions, Bu3 exhibits the most prominent GmA peak.

## Slide 6
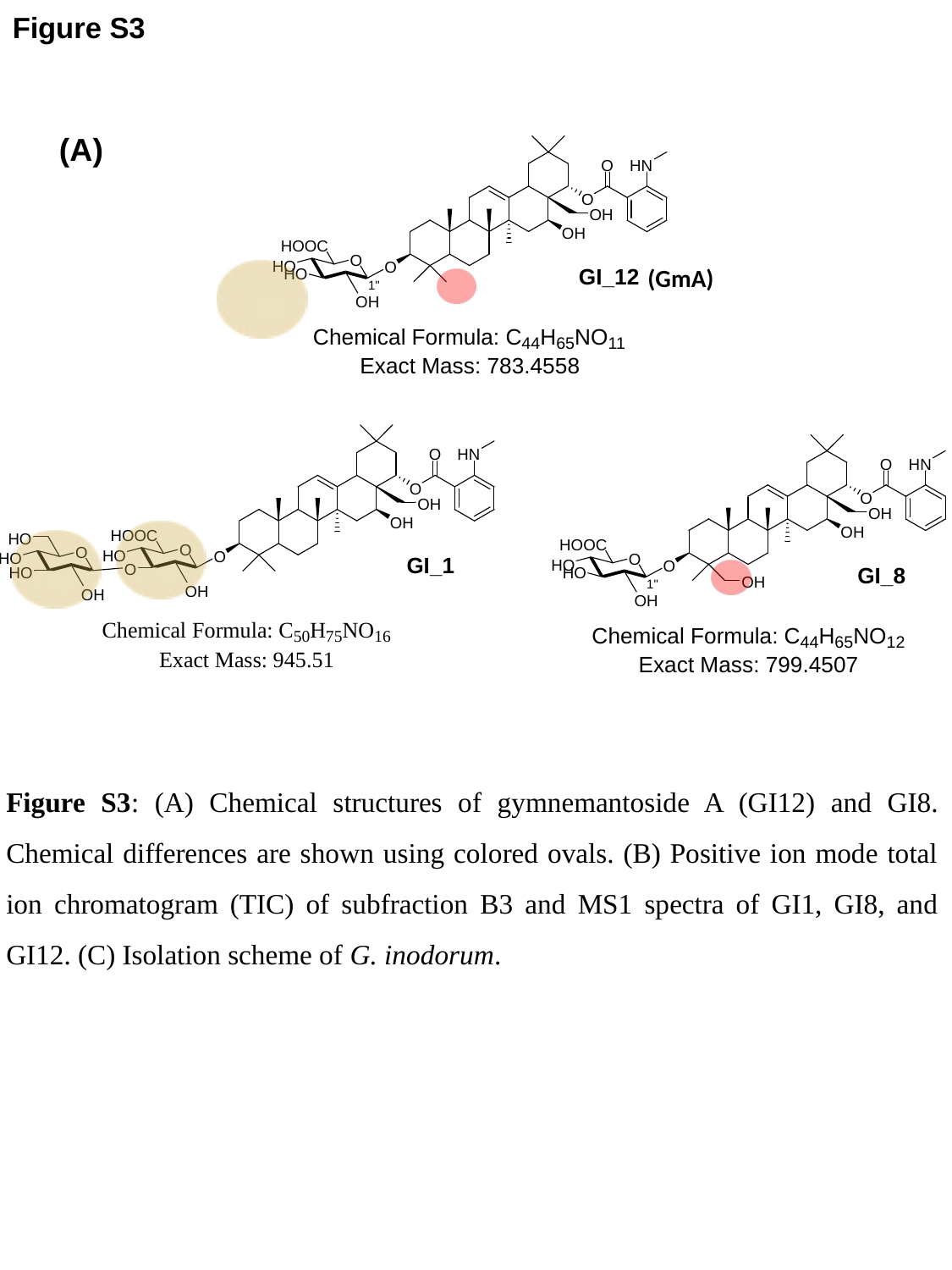

Figure S3
(A)
(GmA)
Figure S3: (A) Chemical structures of gymnemantoside A (GI12) and GI8. Chemical differences are shown using colored ovals. (B) Positive ion mode total ion chromatogram (TIC) of subfraction B3 and MS1 spectra of GI1, GI8, and GI12. (C) Isolation scheme of G. inodorum.

## Slide 7
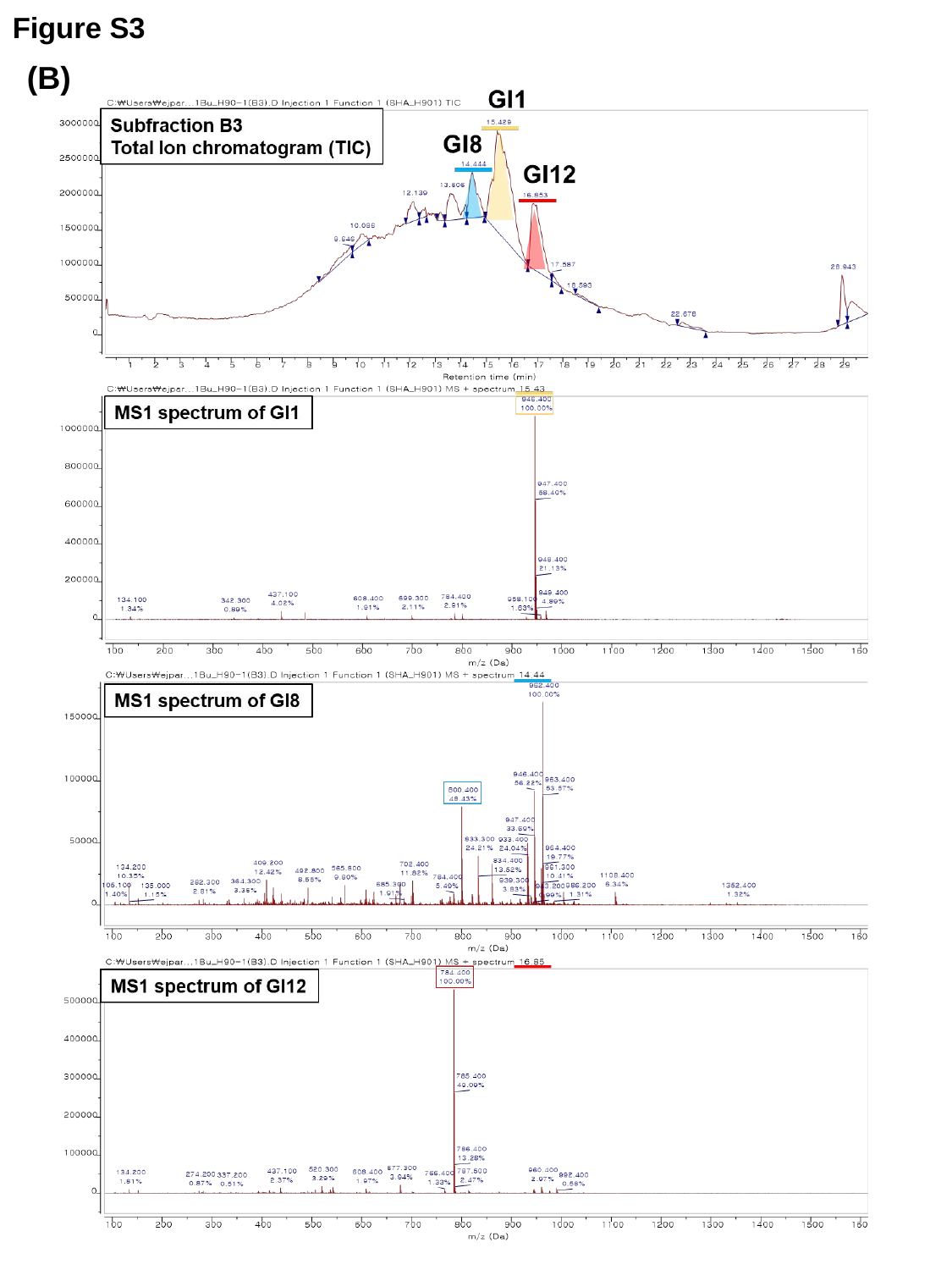

Figure S3
(B)

## Slide 8
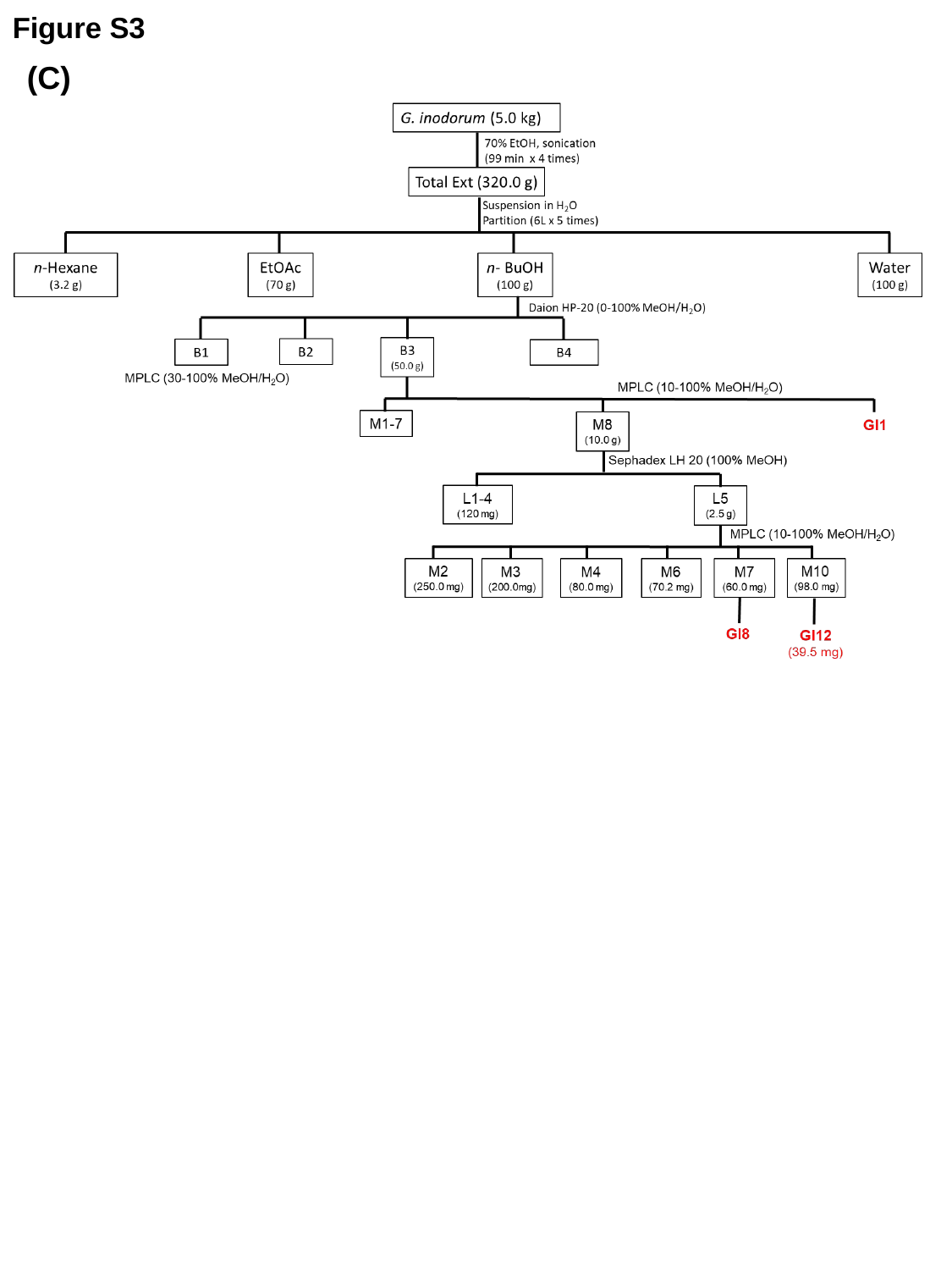

Figure S3
(C)

## Slide 9
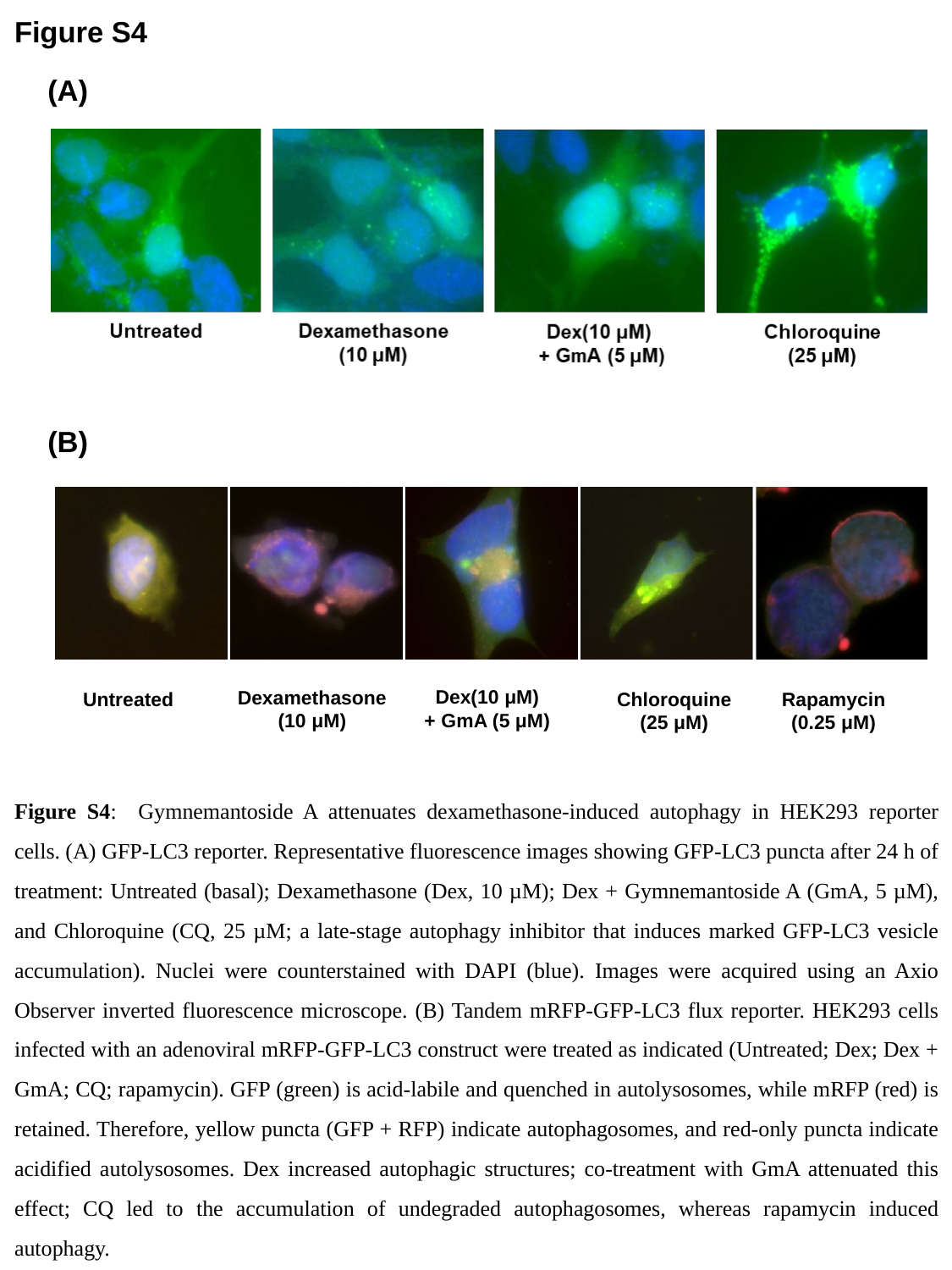

Figure S4
(A)
(B)
Dex(10 μM)
 + GmA (5 μM)
Dexamethasone
(10 μM)
Chloroquine
(25 μM)
Rapamycin
(0.25 μM)
Untreated
Figure S4:  Gymnemantoside A attenuates dexamethasone-induced autophagy in HEK293 reporter cells. (A) GFP-LC3 reporter. Representative fluorescence images showing GFP-LC3 puncta after 24 h of treatment: Untreated (basal); Dexamethasone (Dex, 10 µM); Dex + Gymnemantoside A (GmA, 5 µM), and Chloroquine (CQ, 25 µM; a late-stage autophagy inhibitor that induces marked GFP-LC3 vesicle accumulation). Nuclei were counterstained with DAPI (blue). Images were acquired using an Axio Observer inverted fluorescence microscope. (B) Tandem mRFP-GFP-LC3 flux reporter. HEK293 cells infected with an adenoviral mRFP-GFP-LC3 construct were treated as indicated (Untreated; Dex; Dex + GmA; CQ; rapamycin). GFP (green) is acid-labile and quenched in autolysosomes, while mRFP (red) is retained. Therefore, yellow puncta (GFP + RFP) indicate autophagosomes, and red-only puncta indicate acidified autolysosomes. Dex increased autophagic structures; co-treatment with GmA attenuated this effect; CQ led to the accumulation of undegraded autophagosomes, whereas rapamycin induced autophagy.

## Slide 10
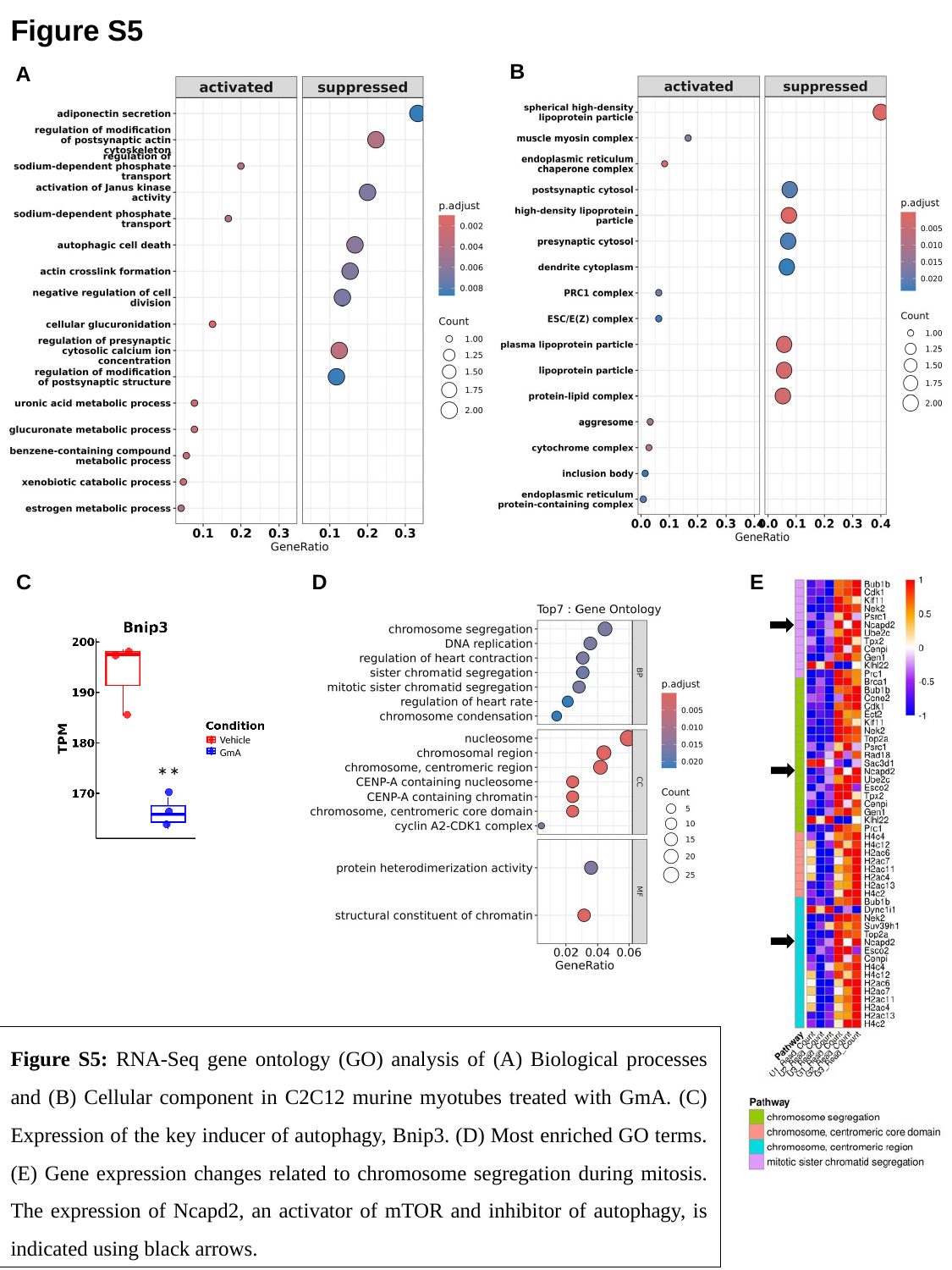

Figure S5
B
A
C
D
E
Vehicle
GmA
**
Figure S5: RNA-Seq gene ontology (GO) analysis of (A) Biological processes and (B) Cellular component in C2C12 murine myotubes treated with GmA. (C) Expression of the key inducer of autophagy, Bnip3. (D) Most enriched GO terms. (E) Gene expression changes related to chromosome segregation during mitosis. The expression of Ncapd2, an activator of mTOR and inhibitor of autophagy, is indicated using black arrows.

## Slide 11
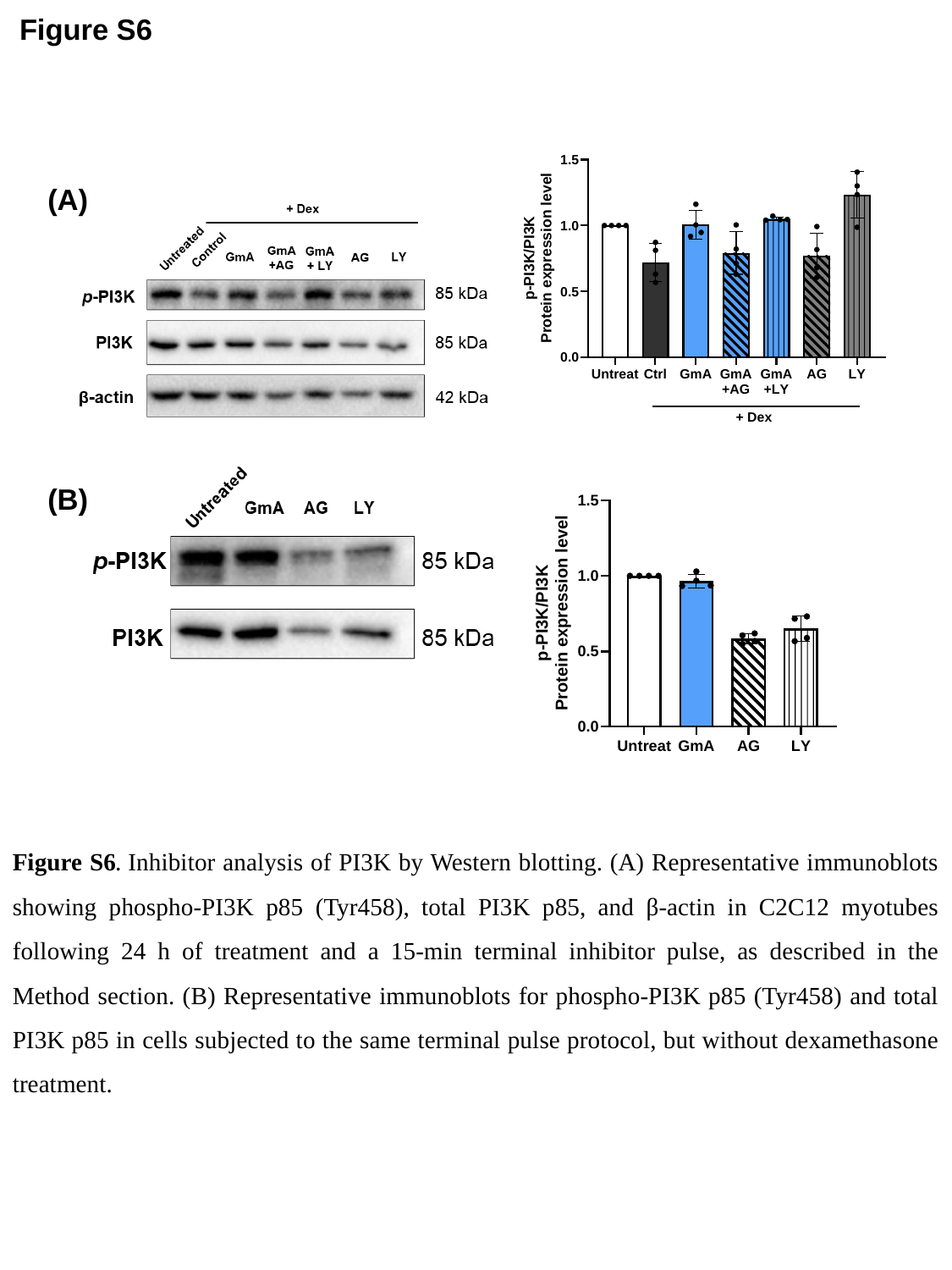

Figure S6
(A)
(B)
Figure S6. Inhibitor analysis of PI3K by Western blotting. (A) Representative immunoblots showing phospho-PI3K p85 (Tyr458), total PI3K p85, and β-actin in C2C12 myotubes following 24 h of treatment and a 15-min terminal inhibitor pulse, as described in the Method section. (B) Representative immunoblots for phospho-PI3K p85 (Tyr458) and total PI3K p85 in cells subjected to the same terminal pulse protocol, but without dexamethasone treatment.

## Slide 12
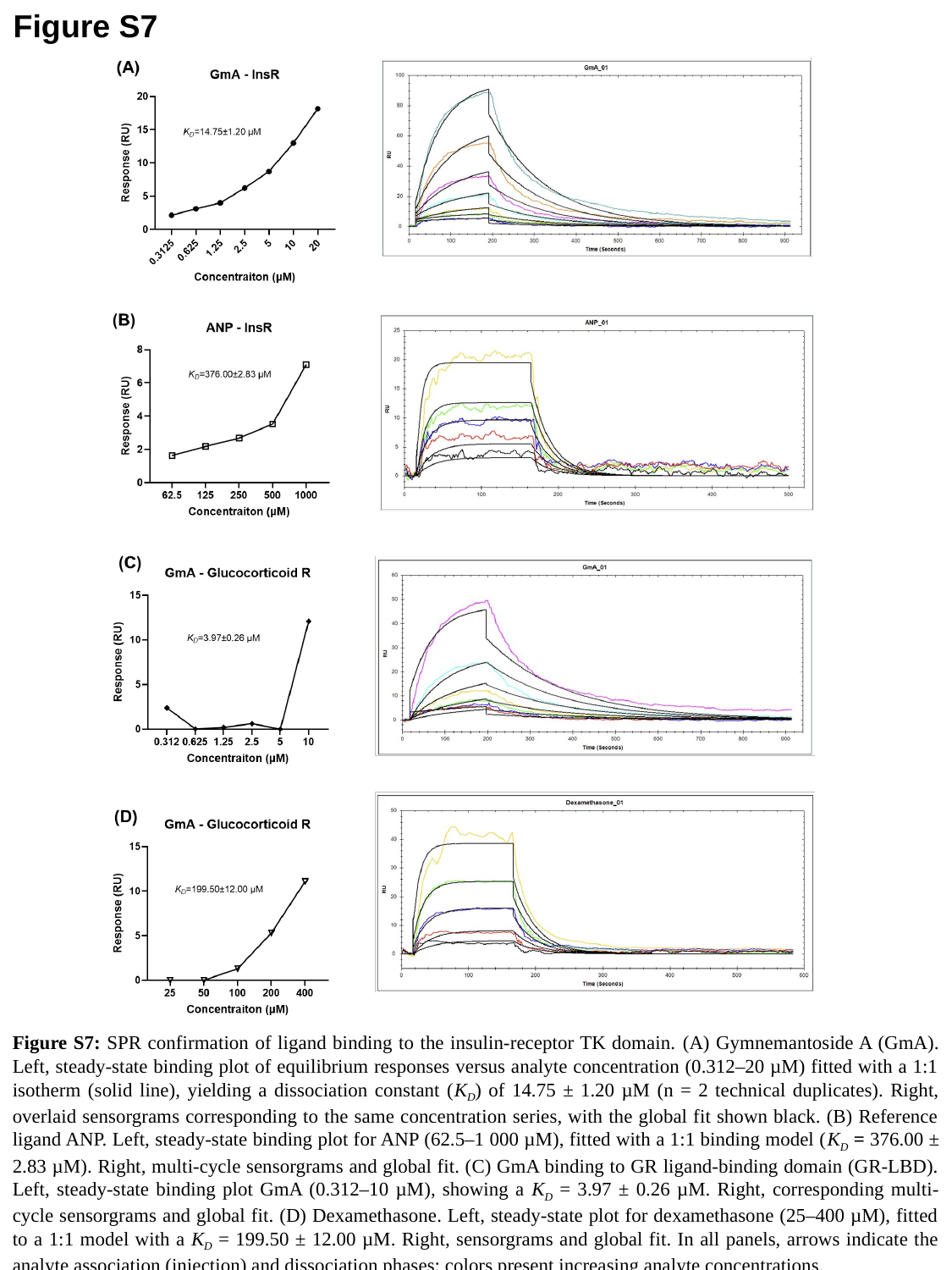

Figure S7
Figure S7: SPR confirmation of ligand binding to the insulin-receptor TK domain. (A) Gymnemantoside A (GmA). Left, steady-state binding plot of equilibrium responses versus analyte concentration (0.312–20 µM) fitted with a 1:1 isotherm (solid line), yielding a dissociation constant (KD) of 14.75 ± 1.20 µM (n = 2 technical duplicates). Right, overlaid sensorgrams corresponding to the same concentration series, with the global fit shown black. (B) Reference ligand ANP. Left, steady-state binding plot for ANP (62.5–1 000 µM), fitted with a 1:1 binding model (KD = 376.00 ± 2.83 µM). Right, multi-cycle sensorgrams and global fit. (C) GmA binding to GR ligand-binding domain (GR-LBD). Left, steady-state binding plot GmA (0.312–10 µM), showing a KD = 3.97 ± 0.26 µM. Right, corresponding multi-cycle sensorgrams and global fit. (D) Dexamethasone. Left, steady-state plot for dexamethasone (25–400 µM), fitted to a 1:1 model with a KD = 199.50 ± 12.00 µM. Right, sensorgrams and global fit. In all panels, arrows indicate the analyte association (injection) and dissociation phases; colors present increasing analyte concentrations.

## Slide 13
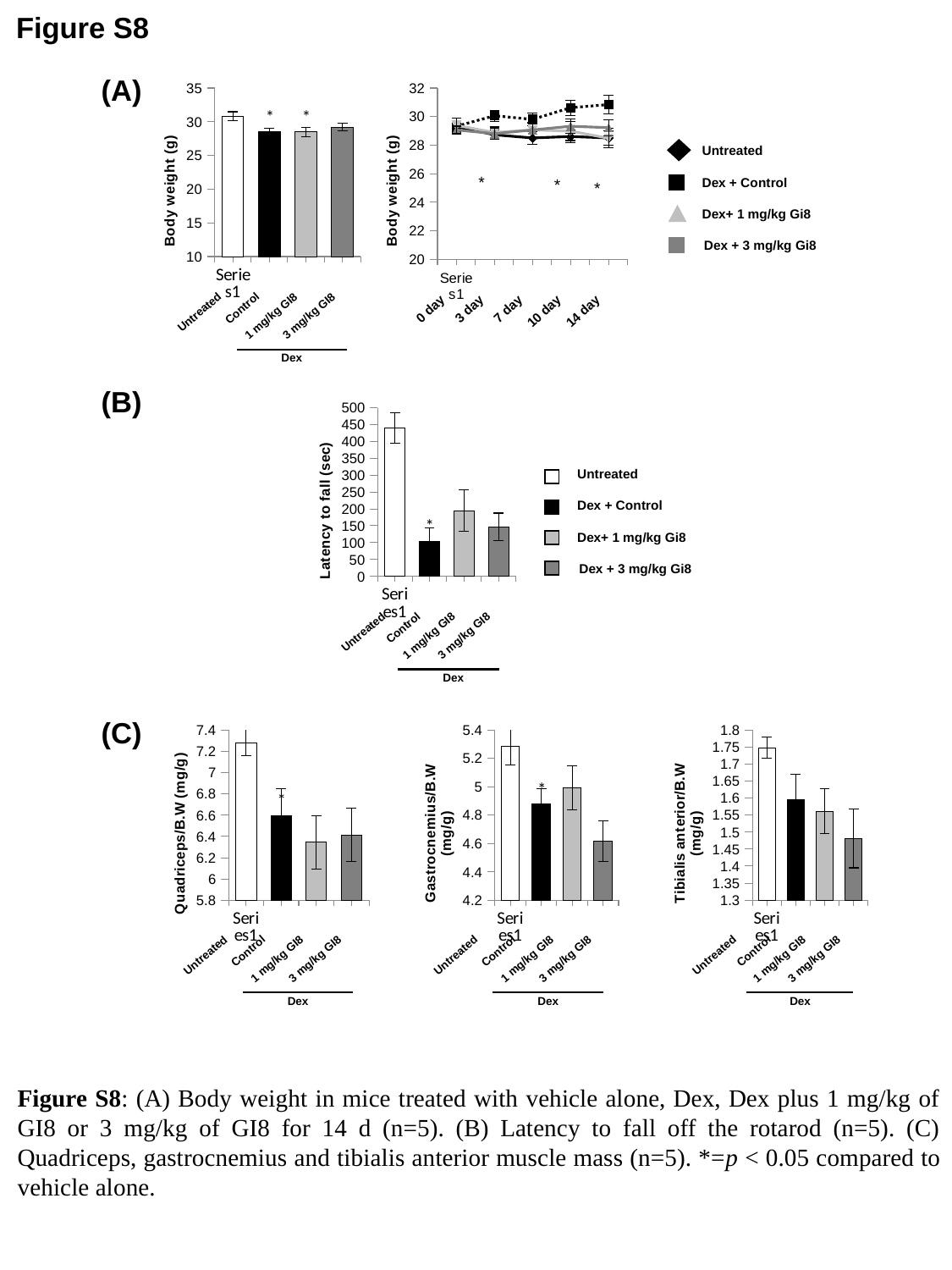

Figure S8
(A)
### Chart
| Category | |
|---|---|
| | 30.840000000000003 |
| | 28.5 |
| | 28.48 |
| | 29.220000000000006 |
### Chart
| Category | | | | 29.06 28.8 29.06 29.32 |
|---|---|---|---|---|
| | 29.320000000000004 | 29.3 | 29.380000000000003 | 29.059999999999995 |
| | 30.060000000000002 | 28.72 | 28.880000000000003 | 28.8 |
| | 29.8 | 28.5 | 29.059999999999995 | 29.060000000000002 |
| | 30.619999999999997 | 28.6 | 28.979999999999997 | 29.32 |
| | 30.840000000000003 | 28.5 | 28.48 | 29.220000000000006 |Untreated
*
Dex + Control
*
*
Dex+ 1 mg/kg Gi8
Dex + 3 mg/kg Gi8
Untreated
Control
1 mg/kg GI8
3 mg/kg GI8
0 day
3 day
7 day
10 day
14 day
Dex
(B)
### Chart
| Category | |
|---|---|
| | 440.4 |
| | 103.6 |
| | 195.0 |
| | 146.8 |Untreated
Dex + Control
Dex+ 1 mg/kg Gi8
Dex + 3 mg/kg Gi8
Untreated
Control
1 mg/kg GI8
3 mg/kg GI8
Dex
(C)
### Chart
| Category | |
|---|---|
| | 7.279830426705738 |
| | 6.597115717392738 |
| | 6.345436120372904 |
| | 6.413473652965598 |
### Chart
| Category | |
|---|---|
| | 5.283789203054255 |
| | 4.878169346473498 |
| | 4.990988096532448 |
| | 4.613938698304866 |
### Chart
| Category | |
|---|---|
| | 1.7479808137674535 |
| | 1.5948002765382074 |
| | 1.5614076847061358 |
| | 1.4815581236444084 |Untreated
Control
1 mg/kg GI8
3 mg/kg GI8
Dex
Untreated
Control
1 mg/kg GI8
3 mg/kg GI8
Dex
Untreated
Control
1 mg/kg GI8
3 mg/kg GI8
Dex
Figure S8: (A) Body weight in mice treated with vehicle alone, Dex, Dex plus 1 mg/kg of GI8 or 3 mg/kg of GI8 for 14 d (n=5). (B) Latency to fall off the rotarod (n=5). (C) Quadriceps, gastrocnemius and tibialis anterior muscle mass (n=5). *=p < 0.05 compared to vehicle alone.

## Slide 14
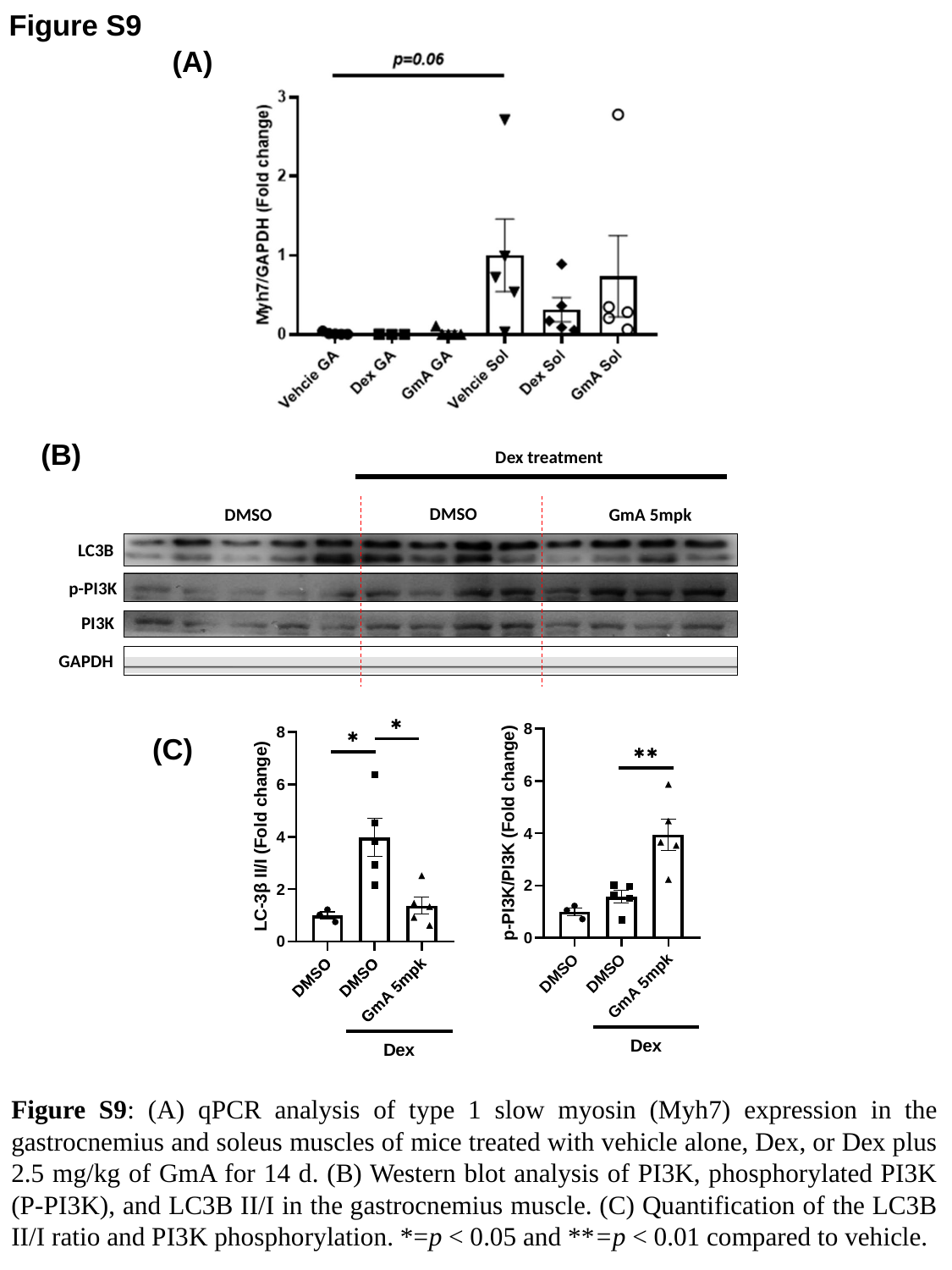

Figure S9
(A)
(B)
Dex treatment
DMSO
GmA 5mpk
DMSO
LC3B
p-PI3K
PI3K
GAPDH
(C)
Figure S9: (A) qPCR analysis of type 1 slow myosin (Myh7) expression in the gastrocnemius and soleus muscles of mice treated with vehicle alone, Dex, or Dex plus 2.5 mg/kg of GmA for 14 d. (B) Western blot analysis of PI3K, phosphorylated PI3K (P-PI3K), and LC3B II/I in the gastrocnemius muscle. (C) Quantification of the LC3B II/I ratio and PI3K phosphorylation. *=p < 0.05 and **=p < 0.01 compared to vehicle.

## Slide 15
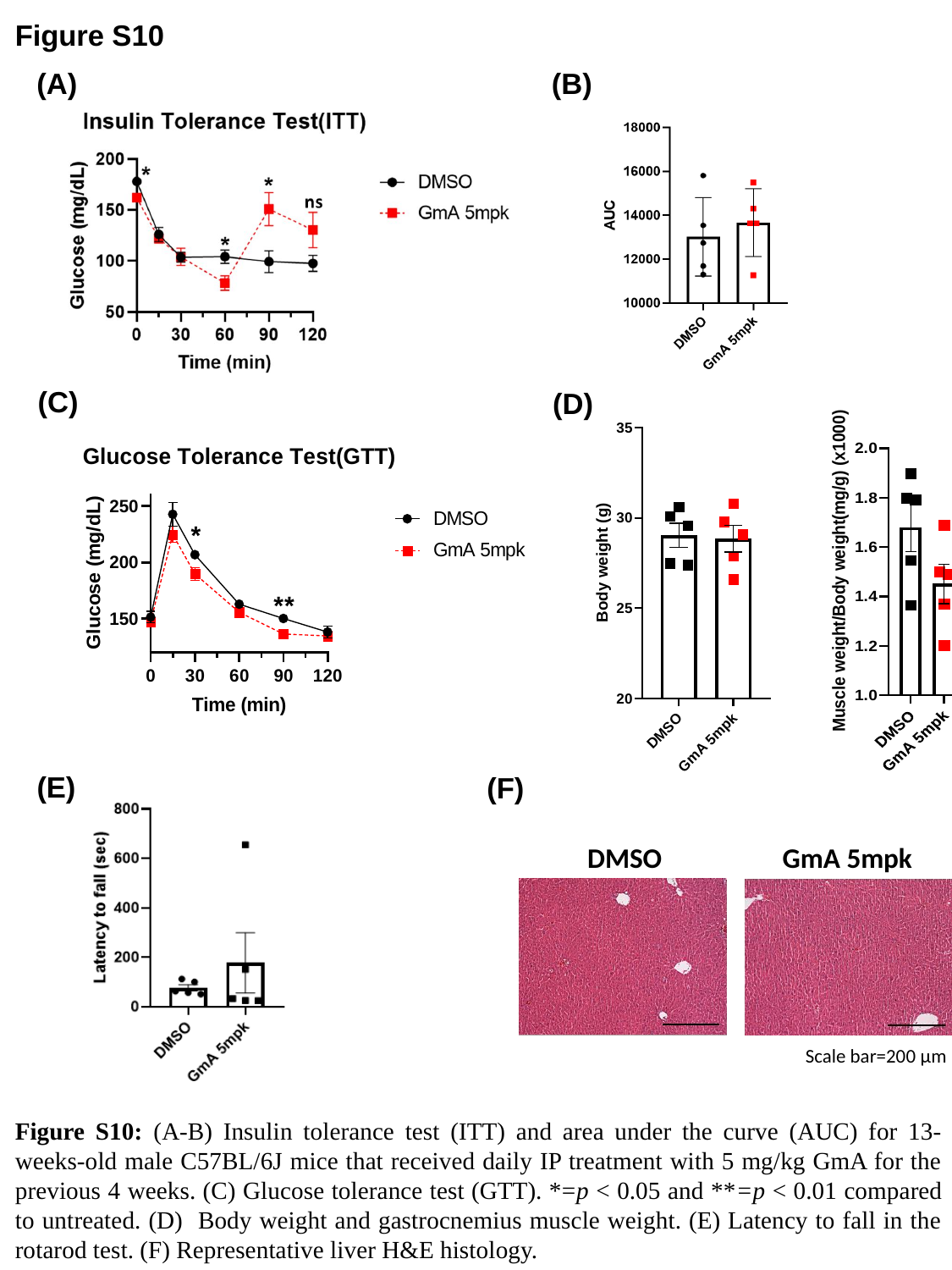

Figure S10
(B)
(A)
(C)
(D)
(E)
(F)
DMSO GmA 5mpk
Scale bar=200 µm
Figure S10: (A-B) Insulin tolerance test (ITT) and area under the curve (AUC) for 13-weeks-old male C57BL/6J mice that received daily IP treatment with 5 mg/kg GmA for the previous 4 weeks. (C) Glucose tolerance test (GTT). *=p < 0.05 and **=p < 0.01 compared to untreated. (D) Body weight and gastrocnemius muscle weight. (E) Latency to fall in the rotarod test. (F) Representative liver H&E histology.

## Slide 16
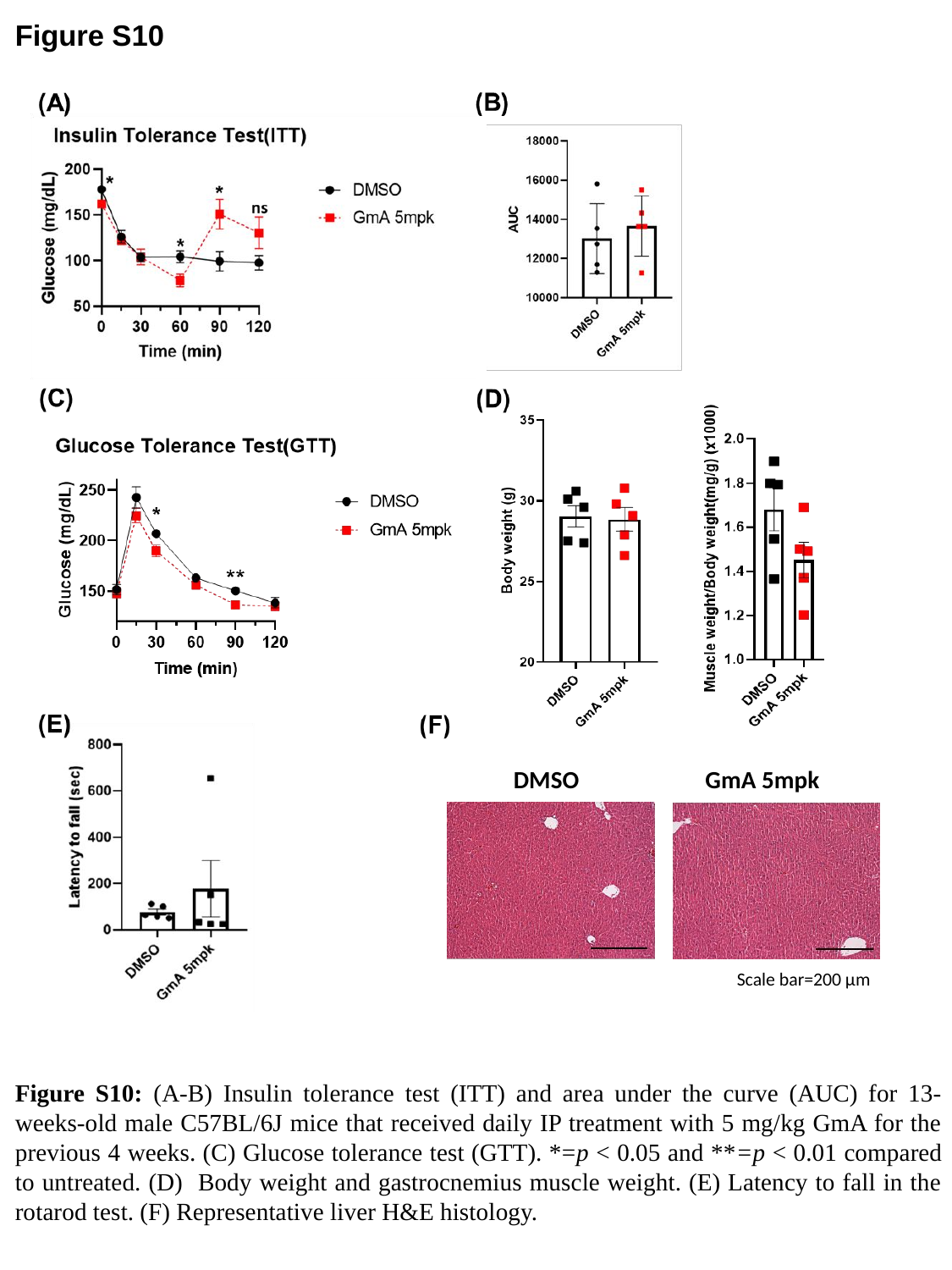

Figure S10
DMSO GmA 5mpk
Scale bar=200 µm
Figure S10: (A-B) Insulin tolerance test (ITT) and area under the curve (AUC) for 13-weeks-old male C57BL/6J mice that received daily IP treatment with 5 mg/kg GmA for the previous 4 weeks. (C) Glucose tolerance test (GTT). *=p < 0.05 and **=p < 0.01 compared to untreated. (D) Body weight and gastrocnemius muscle weight. (E) Latency to fall in the rotarod test. (F) Representative liver H&E histology.

## Slide 17
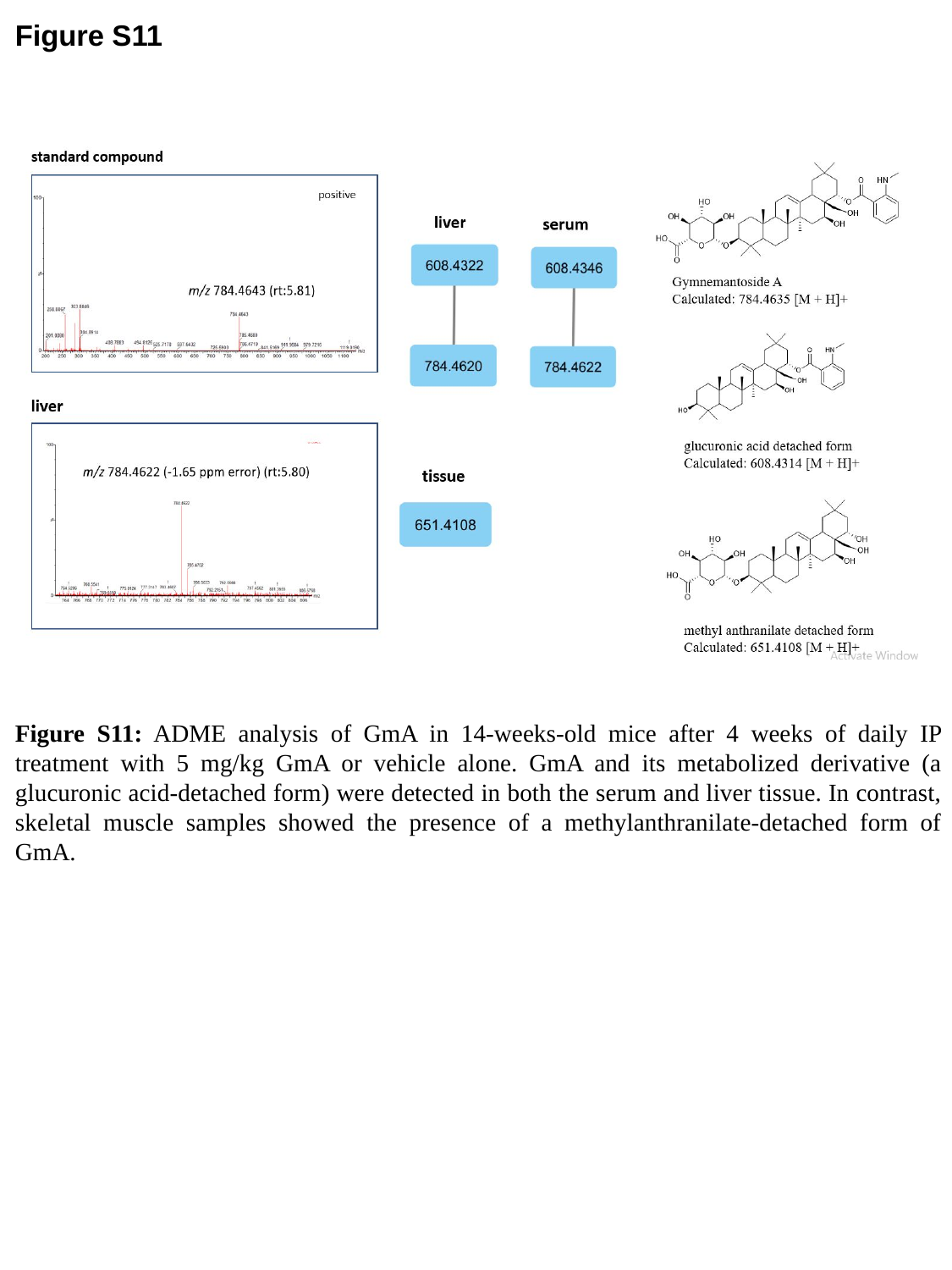

Figure S11
Figure S11: ADME analysis of GmA in 14-weeks-old mice after 4 weeks of daily IP treatment with 5 mg/kg GmA or vehicle alone. GmA and its metabolized derivative (a glucuronic acid-detached form) were detected in both the serum and liver tissue. In contrast, skeletal muscle samples showed the presence of a methylanthranilate-detached form of GmA.

## Slide 18
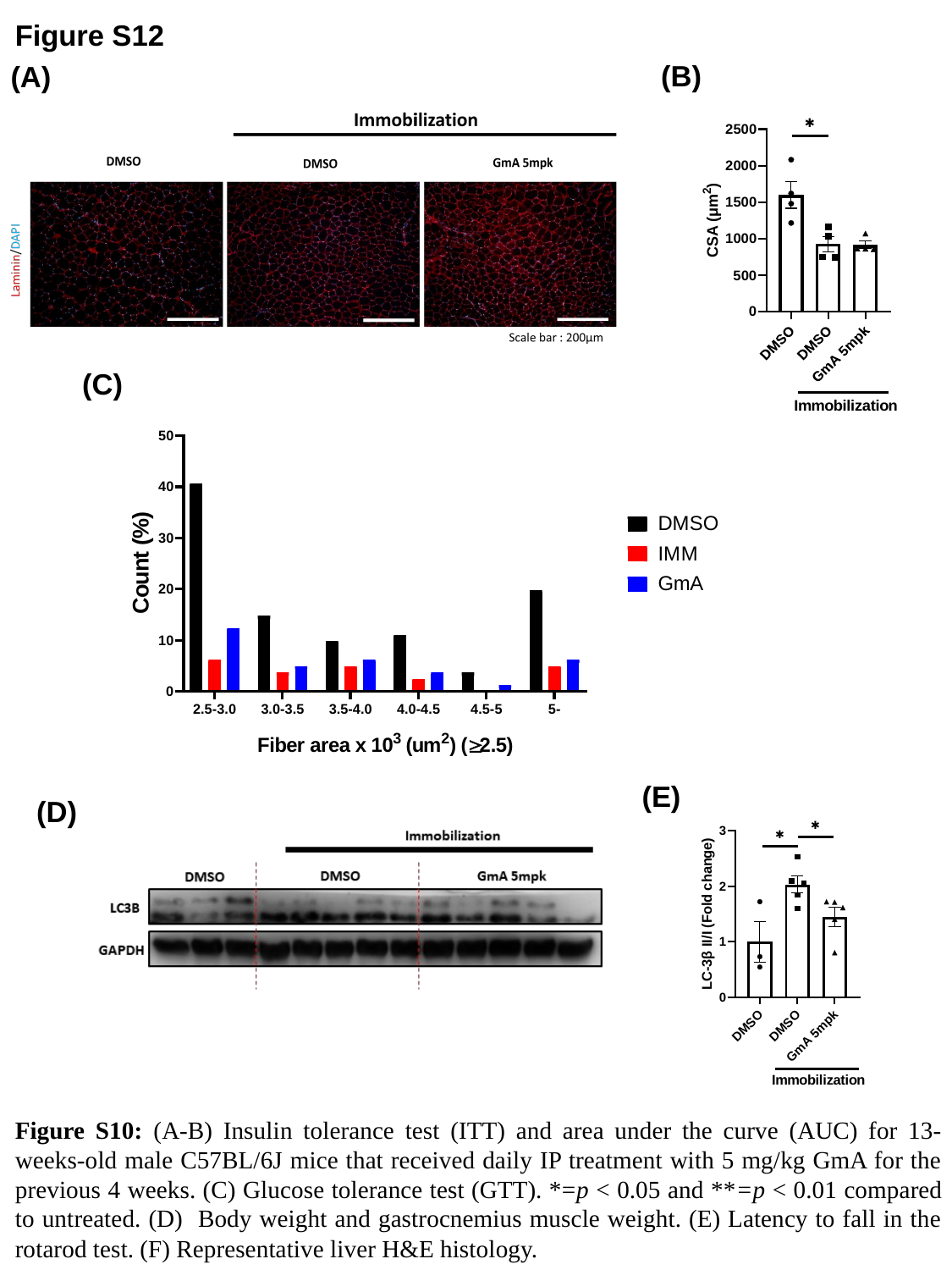

Figure S12
(B)
(A)
(C)
(E)
(D)
Figure S10: (A-B) Insulin tolerance test (ITT) and area under the curve (AUC) for 13-weeks-old male C57BL/6J mice that received daily IP treatment with 5 mg/kg GmA for the previous 4 weeks. (C) Glucose tolerance test (GTT). *=p < 0.05 and **=p < 0.01 compared to untreated. (D) Body weight and gastrocnemius muscle weight. (E) Latency to fall in the rotarod test. (F) Representative liver H&E histology.
