# Supplementary material for: Gymnemantoside A Ameliorates Steroid‐Induced Skeletal Muscle Atrophy via Bridging Glucocorticoid and Insulin Receptor Signalling
Source: J Cachexia Sarcopenia Muscle. 2025 Nov 25;16(6):e70118. doi: 10.1002/jcsm.70118 (PMC12646868; doi:10.1002/jcsm.70118)
Supplement: Supplementary file 2 — Table S1: Primer sequences used in this study. Table S2: jcsm70118‐sup‐0002‐Supplementary_Tables.pptx. 1H and 13C NMR data of gymnemantoside A isolated from G. inodorum. Table S3: Calibration and validation parameters for the HPLC‐UV quantification of gymnemantoside A Table S4: Concentration of gymnemantoside A detected in subfractions Bu1–Bu4 by HPLC‐UV analysis Table S5: Molecular docking analysis result between compound and ligands. Table S6: Surface plasmon resonance (SPR) kinetic (k a , k d ) and equilibrium (K A , K D ) binding parameters for gymnemantoside A (GmA) and reference ligands interacting with the insulin receptor (IR) tyrosine kinase domain and the glucocorticoid receptor (GR) ligand‐binding domain (LBD). Rmax, maximum response (RU); χ2, fit quality. Values are mean ± SD Table S7: Predicted ADME parameters of compound GMA obtained through computational analysis. [file JCSM-16-e70118-s003.pptx]

## Slide 1
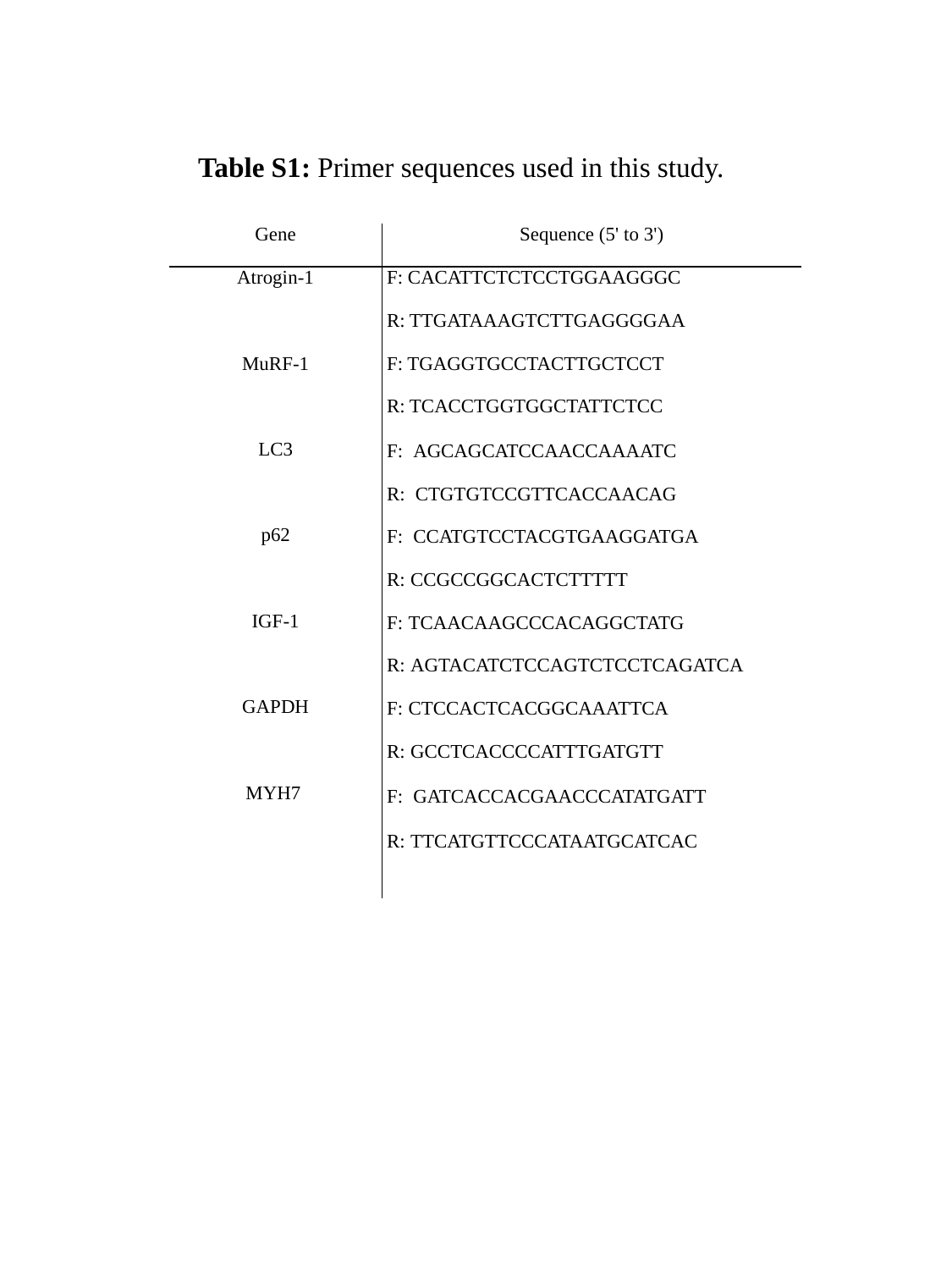

Table S1: Primer sequences used in this study.
| Gene | Sequence (5' to 3') |
| --- | --- |
| Atrogin-1 | F: CACATTCTCTCCTGGAAGGGC |
| | R: TTGATAAAGTCTTGAGGGGAA |
| MuRF-1 | F: TGAGGTGCCTACTTGCTCCT |
| | R: TCACCTGGTGGCTATTCTCC |
| LC3 | F: AGCAGCATCCAACCAAAATC |
| | R: CTGTGTCCGTTCACCAACAG |
| p62 | F: CCATGTCCTACGTGAAGGATGA |
| | R: CCGCCGGCACTCTTTTT |
| IGF-1 | F: TCAACAAGCCCACAGGCTATG |
| | R: AGTACATCTCCAGTCTCCTCAGATCA |
| GAPDH | F: CTCCACTCACGGCAAATTCA |
| MYH7 | R: GCCTCACCCCATTTGATGTT F: GATCACCACGAACCCATATGATT R: TTCATGTTCCCATAATGCATCAC |
| | |
| |
| --- |

## Slide 2
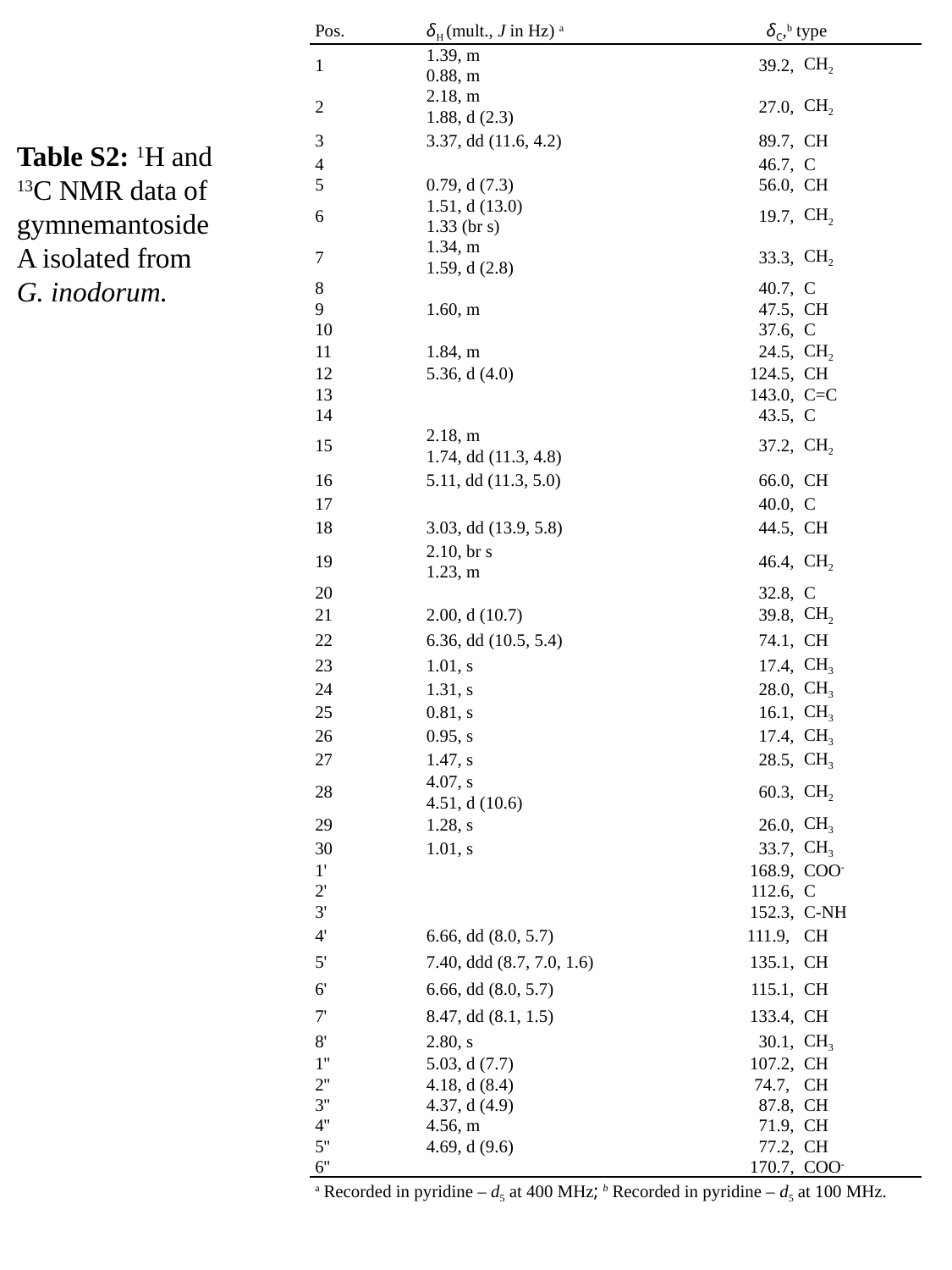

| Pos. | δH (mult., J in Hz) a | δC,b type | |
| --- | --- | --- | --- |
| 1 | 1.39, m 0.88, m | 39.2, | CH2 |
| 2 | 2.18, m 1.88, d (2.3) | 27.0, | CH2 |
| 3 | 3.37, dd (11.6, 4.2) | 89.7, | CH |
| 4 | | 46.7, | C |
| 5 | 0.79, d (7.3) | 56.0, | CH |
| 6 | 1.51, d (13.0) 1.33 (br s) | 19.7, | CH2 |
| 7 | 1.34, m 1.59, d (2.8) | 33.3, | CH2 |
| 8 | | 40.7, | C |
| 9 | 1.60, m | 47.5, | CH |
| 10 | | 37.6, | C |
| 11 | 1.84, m | 24.5, | CH2 |
| 12 | 5.36, d (4.0) | 124.5, | CH |
| 13 | | 143.0, | C=C |
| 14 | | 43.5, | C |
| 15 | 2.18, m 1.74, dd (11.3, 4.8) | 37.2, | CH2 |
| 16 | 5.11, dd (11.3, 5.0) | 66.0, | CH |
| 17 | | 40.0, | C |
| 18 | 3.03, dd (13.9, 5.8) | 44.5, | CH |
| 19 | 2.10, br s 1.23, m | 46.4, | CH2 |
| 20 | | 32.8, | C |
| 21 | 2.00, d (10.7) | 39.8, | CH2 |
| 22 | 6.36, dd (10.5, 5.4) | 74.1, | CH |
| 23 | 1.01, s | 17.4, | CH3 |
| 24 | 1.31, s | 28.0, | CH3 |
| 25 | 0.81, s | 16.1, | CH3 |
| 26 | 0.95, s | 17.4, | CH3 |
| 27 | 1.47, s | 28.5, | CH3 |
| 28 | 4.07, s 4.51, d (10.6) | 60.3, | CH2 |
| 29 | 1.28, s | 26.0, | CH3 |
| 30 | 1.01, s | 33.7, | CH3 |
| 1' | | 168.9, | COO- |
| 2' | | 112.6, | C |
| 3' | | 152.3, | C-NH |
| 4' | 6.66, dd (8.0, 5.7) | 111.9, | CH |
| 5' | 7.40, ddd (8.7, 7.0, 1.6) | 135.1, | CH |
| 6' | 6.66, dd (8.0, 5.7) | 115.1, | CH |
| 7' | 8.47, dd (8.1, 1.5) | 133.4, | CH |
| 8' | 2.80, s | 30.1, | CH3 |
| 1'' | 5.03, d (7.7) | 107.2, | CH |
| 2'' | 4.18, d (8.4) | 74.7, | CH |
| 3'' | 4.37, d (4.9) | 87.8, | CH |
| 4'' | 4.56, m | 71.9, | CH |
| 5'' | 4.69, d (9.6) | 77.2, | CH |
| 6'' | | 170.7, | COO- |
| a Recorded in pyridine – d5 at 400 MHz; b Recorded in pyridine – d5 at 100 MHz. | | | |
Table S2: 1H and 13C NMR data of gymnemantoside A isolated from G. inodorum.

## Slide 3
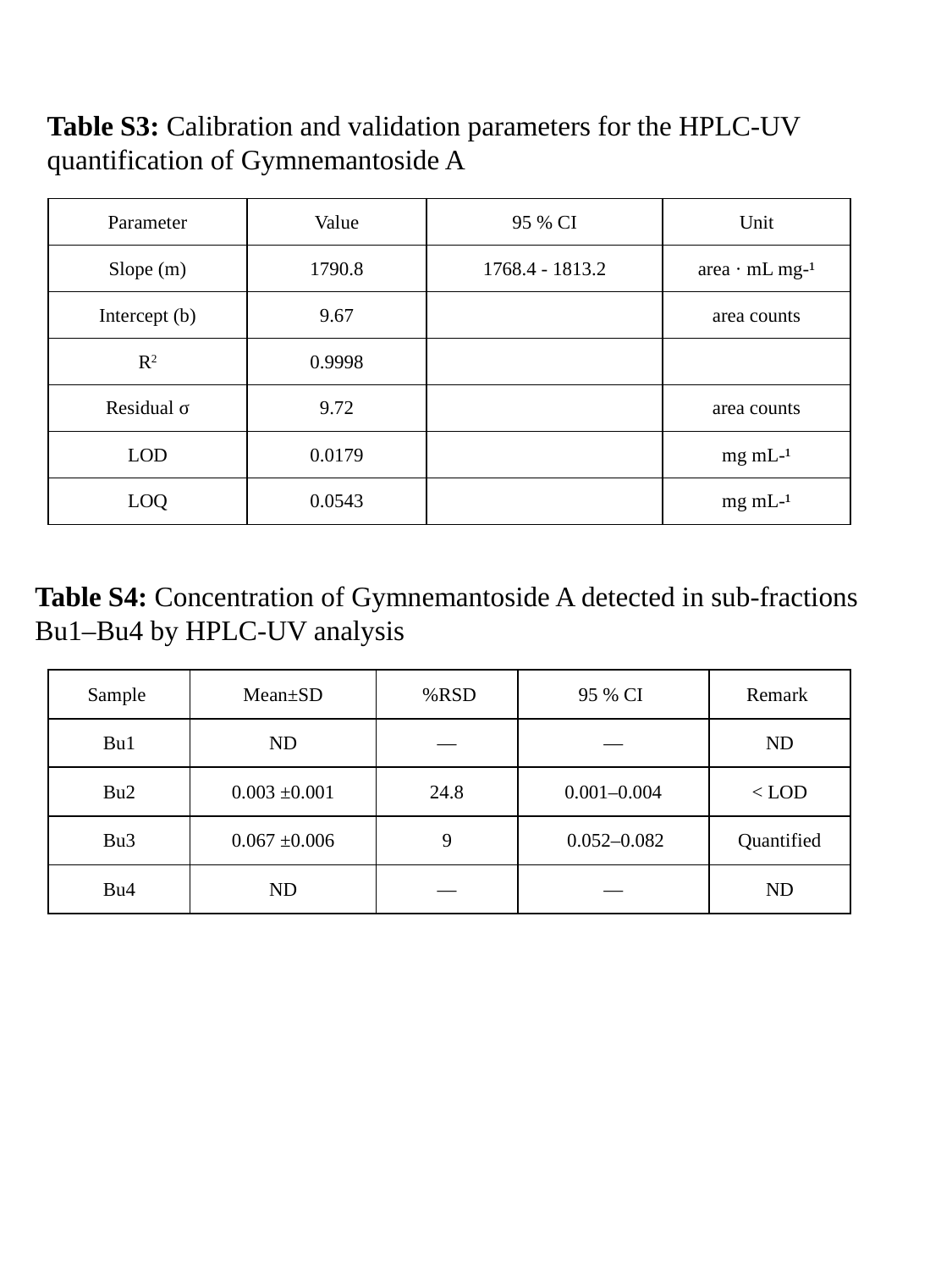

Table S3: Calibration and validation parameters for the HPLC-UV quantification of Gymnemantoside A
| Parameter | Value | 95 % CI | Unit |
| --- | --- | --- | --- |
| Slope (m) | 1790.8 | 1768.4 - 1813.2 | area · mL mg-¹ |
| Intercept (b) | 9.67 | ­ | area counts |
| R2 | 0.9998 | ­ | ­ |
| Residual σ | 9.72 | ­ | area counts |
| LOD | 0.0179 | ­ | mg mL-¹ |
| LOQ | 0.0543 | ­ | mg mL-¹ |
Table S4: Concentration of Gymnemantoside A detected in sub-fractions Bu1–Bu4 by HPLC-UV analysis
| Sample | Mean±SD | %RSD | 95 % CI | Remark |
| --- | --- | --- | --- | --- |
| Bu1 | ND | — | — | ND |
| Bu2 | 0.003 ±0.001 | 24.8 | 0.001–0.004 | < LOD |
| Bu3 | 0.067 ±0.006 | 9 | 0.052–0.082 | Quantified |
| Bu4 | ND | — | — | ND |

## Slide 4
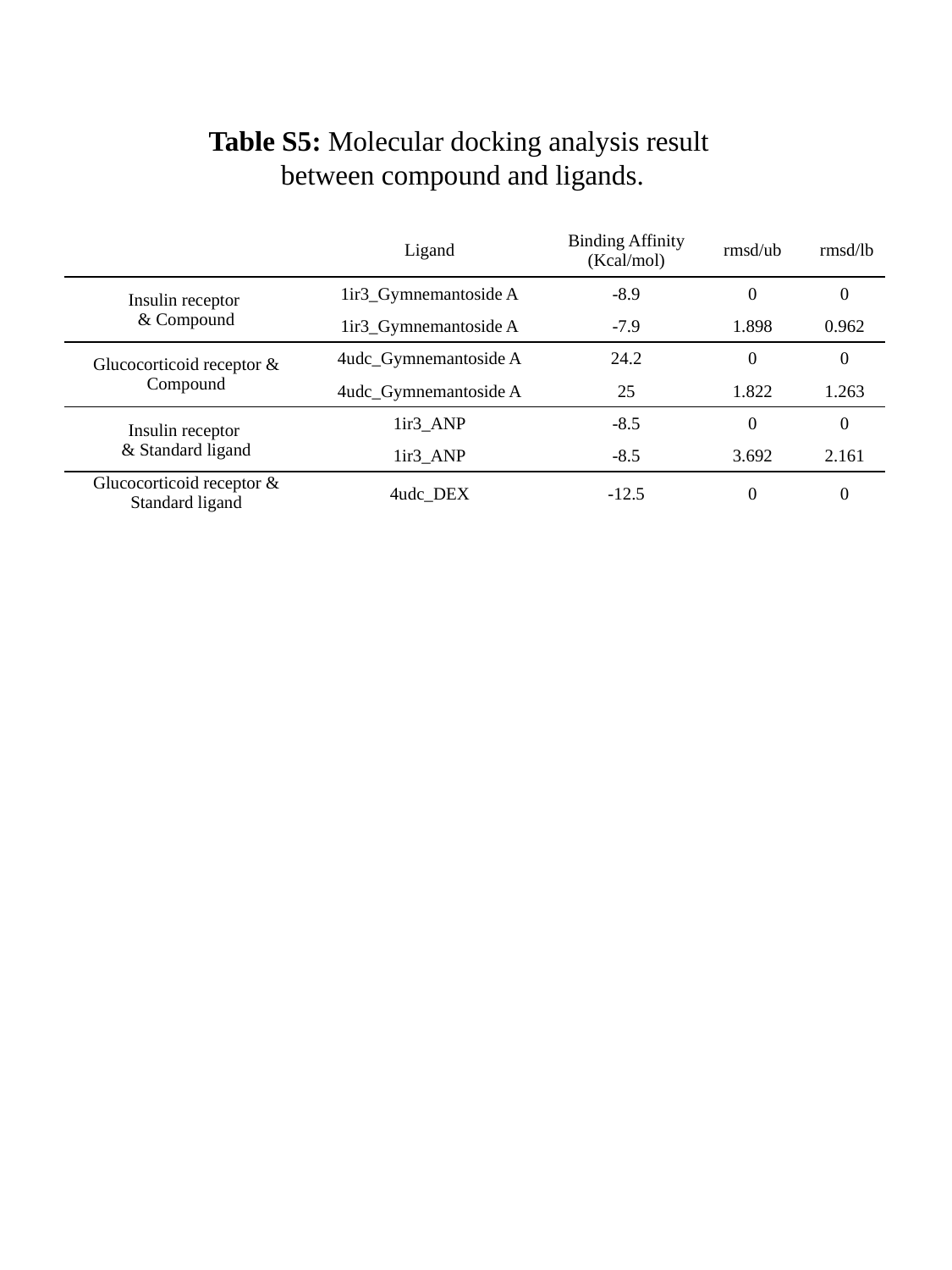

Table S5: Molecular docking analysis result
 between compound and ligands.
| | Ligand | Binding Affinity (Kcal/mol) | rmsd/ub | rmsd/lb |
| --- | --- | --- | --- | --- |
| Insulin receptor & Compound | 1ir3\_Gymnemantoside A | -8.9 | 0 | 0 |
| | 1ir3\_Gymnemantoside A | -7.9 | 1.898 | 0.962 |
| Glucocorticoid receptor & Compound | 4udc\_Gymnemantoside A | 24.2 | 0 | 0 |
| | 4udc\_Gymnemantoside A | 25 | 1.822 | 1.263 |
| Insulin receptor & Standard ligand | 1ir3\_ANP | -8.5 | 0 | 0 |
| | 1ir3\_ANP | -8.5 | 3.692 | 2.161 |
| Glucocorticoid receptor & Standard ligand | 4udc\_DEX | -12.5 | 0 | 0 |

## Slide 5
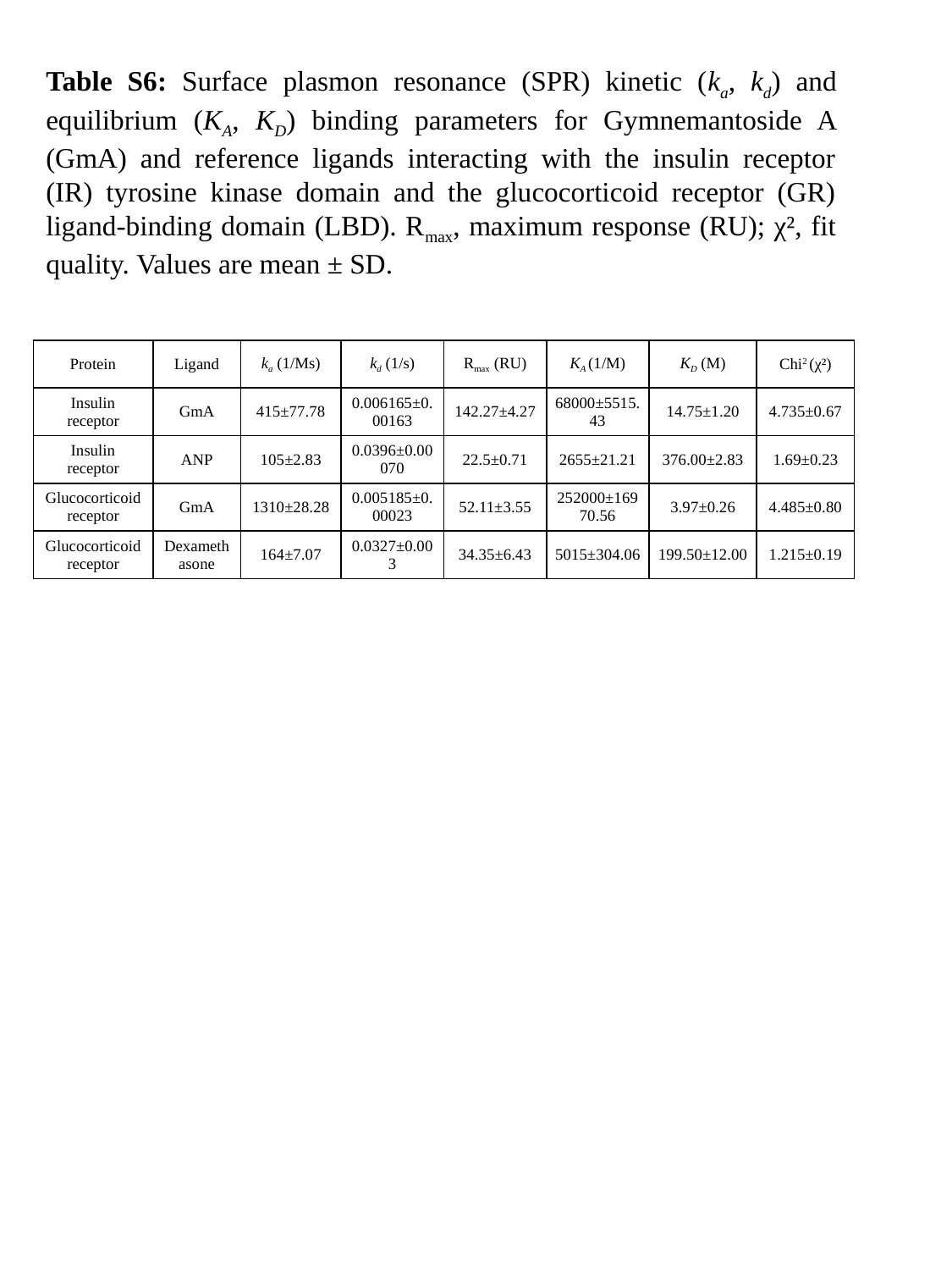

Table S6: Surface plasmon resonance (SPR) kinetic (ka, kd) and equilibrium (KA, KD) binding parameters for Gymnemantoside A (GmA) and reference ligands interacting with the insulin receptor (IR) tyrosine kinase domain and the glucocorticoid receptor (GR) ligand-binding domain (LBD). Rmax, maximum response (RU); χ², fit quality. Values are mean ± SD.
| Protein | Ligand | ka (1/Ms) | kd (1/s) | Rmax (RU) | KA (1/M) | KD (M) | Chi2 (χ²) |
| --- | --- | --- | --- | --- | --- | --- | --- |
| Insulin receptor | GmA | 415±77.78 | 0.006165±0.00163 | 142.27±4.27 | 68000±5515.43 | 14.75±1.20 | 4.735±0.67 |
| Insulin receptor | ANP | 105±2.83 | 0.0396±0.00070 | 22.5±0.71 | 2655±21.21 | 376.00±2.83 | 1.69±0.23 |
| Glucocorticoid receptor | GmA | 1310±28.28 | 0.005185±0.00023 | 52.11±3.55 | 252000±16970.56 | 3.97±0.26 | 4.485±0.80 |
| Glucocorticoid receptor | Dexamethasone | 164±7.07 | 0.0327±0.003 | 34.35±6.43 | 5015±304.06 | 199.50±12.00 | 1.215±0.19 |

## Slide 6
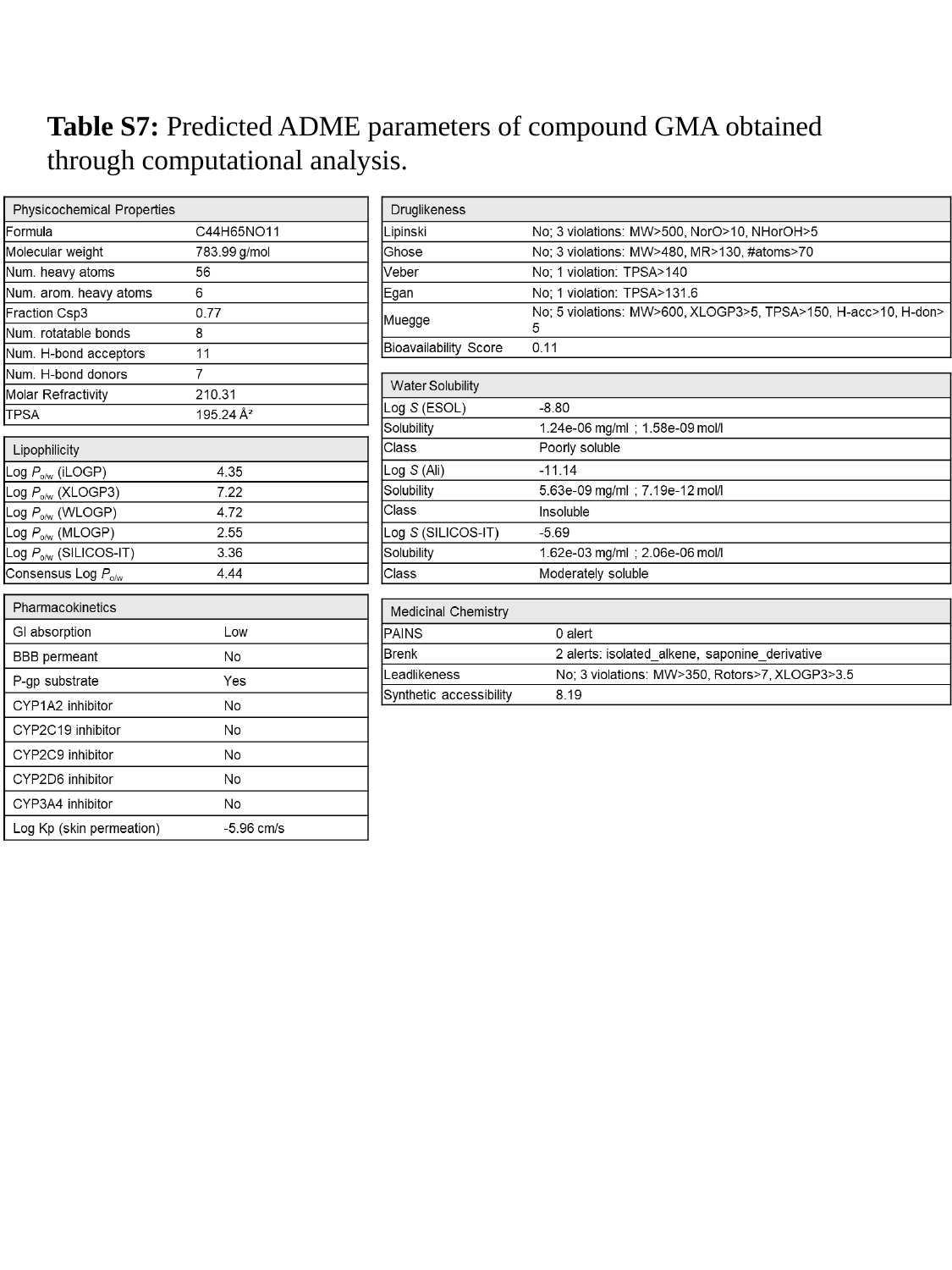

Table S7: Predicted ADME parameters of compound GMA obtained through computational analysis.
