# Supplementary material for: Gymnemantoside A Ameliorates Steroid‐Induced Skeletal Muscle Atrophy via Bridging Glucocorticoid and Insulin Receptor Signalling
Source: J Cachexia Sarcopenia Muscle. 2025 Nov 25;16(6):e70118. doi: 10.1002/jcsm.70118 (PMC12646868; doi:10.1002/jcsm.70118)
Supplement: Supplementary file 3 — Data S1: Supplementary Information. [file JCSM-16-e70118-s002.docx]

**Supplementary Methods and Discussion:**

**Gymnemantoside A ameliorates steroid-induced skeletal muscle atrophy via bridging glucocorticoid and insulin receptor signaling**

Eun-Jin Park^1^, Hyun-Jun Kim^2^, Sang-Hoon Lee^2^, Seri Choi^1^, Thi-Phuong Doan^1^, Kyoung-Hwan Joo^2^, Jin-Pyo An^1^, Da-Woon Jung^2^, Darren Reece Williams^2^ and Won Keun Oh^1^

^1^Research Institute of Pharmaceutical Sciences, College of Pharmacy, Seoul National University, Seoul 08826, Republic of Korea

^2^New Drug Targets Laboratory, School of Life Sciences, Gwangju Institute of Science and Technology, 1 Oryong-Dong, Buk-Gu, Gwangju 61005, Republic of Korea

**Supplementary Methods**

**General Experimental Procedures.**

Analytical grade solvents were obtained from Sigma-Aldrich (St. Louis, MO, USA), while solvents for extraction were sourced from Daejung Chemicals & Metals Co. (Siheung, Korea). Optical rotations were recorded using a JASCO P2000 polarimeter from JASCO International Co. Ltd. (Tokyo, Japan). IR data were collected with a Nicolet 6700 FT-IR from Thermo Fisher Scientific (Waltham, MA, USA). The ^1^H and ^13^C NMR spectra were measured on a JEOL 400 or 600 MHz NMR Spectrometers (JEOL, Tokyo, Japan). High-Resolution Electrospray Ionization Mass Spectrometry (HRESIMS) data were obtained using an Agilent 6530 Q-TOF mass spectrometer, equipped with an Agilent 1260 Infinity HPLC (Agilent Technologies, Santa Clara, CA, USA). For column chromatography, silica gel (63−200 *μ*m) and RP-C18 (40−63 *μ*m) resins were purchased from Merck (Darmstadt, Germany), and Sephadex LH-20 was acquired from Sigma-Aldrich. RP-18 TLC and silica gel 60 F254 plates were utilized for TLC analysis. HPLC analysis was performed on a Gilson HPLC system using an Optima Pak C18 column (10 mm × 250 mm, 10 *μ*m; RS Tech, Seoul, Korea). Quantitative HPLC analysis was performed using an Agilent 1100 Series system (Agilent Technologies, Santa Clara, CA, USA) equipped with a HECTOR-M C18 column (4.6 × 250 mm, 5 µm; RStech, Daejeon, Republic of Korea). Medium-pressure liquid chromatography (MPLC) was performed using a Biotage-Isolera One system (Biotage, Charlotte, NC, USA), equipped with a Revelevis ® Buchi RP-C18 column (40 *μ*m*,* 4 × 20 cm i.d).

**Plant Material.**

The leaves of *Gymnema inodorum* were collected in September 2018 in Hoai Duc district of Hanoi City, Vietnam (20°59′30.6″ N 105°43′49.8″ E). The sample was botanically identified by Dr. Ha Thanh Tung Pham of PHENIKAA University in Vietnam. A voucher specimen (SNU2018-11) has been deposited in the herbarium of the College of Pharmacy at Seoul National University in Korea.

**Isolation of the New Compound 1 and Its Physico-Chemical Properties.**

The leaves of *G. inodorum* (5.0 kg) were extracted three times with 70% EtOH (15 L) for one day each at room temperature. The resulting crude extract (320 g) was partitioned with *n*-hexane (3 × 10 L), EtOAc (3 × 10 L), *n*-BuOH (3 × 10 L), and H_2_O. The *n*-BuOH-soluble partition (100 g) was then subjected to chromatography on a Diaion HP-20 column, yielding four fractions. **Fraction B3 (H90-1, eluted by 90%MeOH/H_2_O) (50 g) underwent further chromatography using MPLC with an RP-C_18_ column (Revelevis ® Buchi, C-18 40 *μ*m*,* 4 × 20 cm i.d.), eluting with a MeOH/H_2_O (10–100%, over 120 min), resulting in nine subfractions. Subsequently, Fraction M8 (10.0 g) was loaded onto an MPLC, eluting with 10 – 100 % MeOH/H_2_O gradient and using a Watcher ® Flash Cartridge C18 (40-63 *μ*m*,* 3 × 14 cm i.d.) and Sephadex LH-20 with 100 % MeOH, to yield subfraction L5 (2.5 g). This subfraction was then further separated to isolate new compound 1 (39.5 mg).**

**Chemical Structure Determination of New Compound 1 (GmA)**

The leaves of *G. inodorum* (5.0 kg) were extracted to obtain 70% EtOH extract (320 g). The extract was partitioned with *n*-hexane, EtOAc, *n*-BuOH, and H_2_O. The bioactive *n*-BuOH-soluble fraction (100 g) was subjected to column chromatography and MPLC equipped with an RP-C_18_ and a Sephadex LH-20, resulting in the isolation of compound **1** (50.0 mg). Compound **1** was obtained as a white, amorphous powder, exhibiting a specific optical rotation of ${[\alpha]}_{D}^{25}$ +12.7 (*c* 0.1, MeOH). Its molecular formula, C_44_H_65_NO_11_, was deduced from the HRESIMS ion peak at *m/z* 784.4612 [M + H]^+^, (calcd for C_44_H_65_NO_11_, 784.4636), indicating 12 degrees of unsaturation. The IR spectrum showed bands at 3365, 1608, and 1514 cm^−1^, characteristic of NH stretching, olefinic, and NH bending absorbance. The ^1^H NMR spectrum revealed signals for four aromatic protons (*δ*_H_ 8.47, dd, *J* = 8.1, 1.5 Hz, H-7'; 7.40, ddd, *J* = 8.7, 7.0, 1.6 Hz, H-5'; 6.66, dd, *J* = 8.0, 5.7 Hz, H-4', H-6'), one olefinic proton (*δ*_H_ 5.36, d, *J* = 4.0 Hz, H-12), an anomeric proton (*δ*_H_ 5.03 (d, *J* = 7.7 Hz, H-1''), one nitrogenated methyl group (*δ*_H_ 2.80, s, H_3_-8'), and seven methyl groups (*δ*_C_ 1.47, 1.31, 1.28, 1.01 (6H), 0.95, and 0.81). The ^13^C NMR spectrum displayed resonances for two carbonyl carbons (*δ*_C_ 170.7 and 168.9), six aromatic carbons (*δ*_C_ 152.3, 135.1, 133.4, 115.1, 112.6, and 111.9), two olefinic carbons (*δ*_C_ 143.0 and 124.5), and one anomeric carbon (*δ*_C_ 107.2), suggesting the structure of compound **1** as a pentacyclic triterpene aglycone with one glucuronic acid and an *N*-methyl anthranilate (Mant) moiety. The HMBC correlations from the nitrogenated methyl signal at *δ*_H_ 2.80 to an aromatic carbon (*δ*_C_ 152.3, C-3') and from the aromatic proton signal at *δ*_H_ 8.47 to a carbonyl carbon (*δ*_C_ 168.9, C-1') suggested the presence of an anthranilate group. The position of this group was confirmed by the HMBC correlation from H-22 (*δ*_H_ 6.36, dd, *J* = 10.5, 5.4 Hz) to C-1'. COSY correlations for H_2_-15 (*δ*_H_ 2.18, 1.74)/H-16 (*δ*_H_ 5.11) and H-21 (*δ*_H_ 2.00)/H-22 (*δ*_H_ 6.36) indicated that C-16 and C-22 were oxygenated. The oxygenated methylene group at C-17 was confirmed based on an HMBC correlation from H­_2_-28 (*δ*_H_ 4.51, 4.07) to C-17 (*δ*_C_ 40.0). The presence of a glucuronic acid moiety was deduced from the HMBC correlation between H-5'' of glucuronic acid (GlcA) (*δ*_H_ 4.69) and C-6'' (*δ*_C_ ­170.7). In the HMBC spectrum, the anomeric proton of the glucuronic acid unit showed a correlation with C-3 (*δ*_C_ 89.7) of the aglycone. The *α*-orientation of the *N*-methyl anthranilate moiety at C-22 was inferred from the NOESY correlation between H-18 (*δ*_H_ 3.03, dd, *J* = 13.9, 5.8 Hz) and H-22 (*δ*_H_ 6.36). The NMR data observed for compound **1** were similar to those of (3*β*,16*β*,22*α*)-22-(*N*-methylanthraniloxy)-16,28-dihydroxyolean-12-en-3-yl-3-O-*β*-D-glucopyranosyl-*β*-D-glucopyranosiduronic acid [1], except for the absence of a glucose unit. Therefore, compound **1** was identified as (3*β*,16*β*,22*α*)-22-(*N*-methylanthraniloxy)-16,28-dihydroxyolean-12-en-3-yl-*β*-D-glucopyranosiduronic acid and named as gymnamantoside A (GmA) (Figure 2A-C and Figure S1 and S2).

**Cell Viability Assay**

C2C12 myoblasts were seeded onto 96-well plates (1 × 10^4^ cells/well) and cultured in DMEM medium with 10% FBS for 24 h. The cells were then treated with the tested compounds dissolved in DMEM medium for an additional 24 h. Cell viability was assessed using the 3-(4,5-dimethyl-2-thiazolyl)-2,5-diphenyl-2H-tetrazolium bromide (MTT) assay (Sigma-Aldrich). Twenty microliters of a 2 mg/mL MTT solution was added to each well, and the plates were incubated for 3 h at 37 °C in the dark. Afterwards, the supernatant was discarded, and the formazan crystals that had formed were dissolved in 100 μL of DMSO. Absorbance was measured at 570 nm using a microplate reader (VersaMaxTM, Randor, PA, USA).

**Detection of autophagy activity in vitro**

Autophagy activity in C2C12 myoblast cells and HEK293 kidney cells were detected using GFP-mRFP-LC3 (ptf-LC3) constructs (Addgene, MA, USA). Cells were seeded onto sterilized cover glass in a 6 well plate, incubated overnight, and transfected with the constructs using Lipofectamine 2000 (Thermo Fisher Scientific, Waltham, MA, USA) based on the manufacturer's instructions. After transfection, the cells were treated with Dex, or Dex plus Gymnemantoside A (GmA) (1 or 5 μM) for 24 h. The cells were then fixed for 10 min in 3.7 % paraformaldehyde and mounted on glass slides using ProLong Gold Antifade Mountant (Thermo Fisher). Confocal images of the LC3 puncta were acquired using confocal microscopy (TCS8, LEICA, Wetzlar, Germany).

**Real-time Reverse Transcription-quantitative Polymerase Chain Reaction (RT-qPCR).**

The process of extracting mRNA from myotubes and skeletal muscle and performing RT-qPCR was carried out as previously described [2] with the following modifications. Total RNA was isolated using TRIzol® reagent (Life Technologies, Carlsbad, CA, USA). A quantity of 1.0 µg of total RNA extracted from the cells was reverse-transcribed using an oligo(dT) primer and M-MLV reverse transcriptase (Bioneer, Eumsung, Korea) to synthesize cDNA. Amplicons were measured directly by monitoring the increase in the reporter dye (ROX). The sequences of the primers in this study are presented in Table S1. The quantity of each transcript was calculated as described in the manual for QuantStudioTM 5 (Applied Biosystems, Waltham, MA, USA) and normalized to the amount of GAPDH.

**Western Blot Analysis**

The protein for analysis was extracted from myotubes using a lysis buffer [50 mM Tris-HCl (pH 7.6), 50 mM NaF, 1 mM EDTA, 120 mM NaCl, 0.5% NP-40] containing protease and phosphatase inhibitors (Roche, Basel, Switzerland). After treatment, the cells were washed twice with PBS and then resuspended in the lysis buffer cocktail. The lysed cells were scraped off and centrifuged for 15 min at 4°C at 12,000 rpm. The supernatant, containing the protein extract, was collected. Protein concentration was quantified using a protein assay kit (Bio-Rad Laboratories, Inc., CA, USA). To standardize the protein concentrations, the extract volumes were adjusted with the same lysis buffer. An equal amount of protein (30µg) from each sample was loaded for SDS-PAGE and subsequently transferred to polyvinylidene fluoride (PVDF) membranes (PVDF 0.45 µm, Immobilon-P, USA). Blocking was performed with 5% (w/v) nonfat milk for 1 h with shaking at room temperature. After blocking, the membranes were incubated with primary antibodies overnight at 4°C. This was followed by incubation with secondary antibodies for 1 h at room temperature. Band signals were detected using the ECL solution method. The results were visualized with an ImageQuant™ LAS 4000 or LAS 500 (GE Healthcare, Chicago, IL, USA) image processing system.

**Immunofluorescence Imaging**

Myotubes were visualized using immunofluorescence staining for myosin heavy chain 2, as previously described [3]. Briefly, after treating C2C12 myotubes with the selected compound, the cells were fixed using 4% paraformaldehyde for 30 min at 4 °C. Subsequently, they were washed twice with phosphate-buffered saline (PBS) for 5 min each. The next step involved permeabilizing the cells with 1% Triton X-100 in PBS. Following permeabilization, the cells were blocked using PBS containing 3% bovine serum albumin (BSA) and 0.01% Tween 20 (PBST) for 30 min at room temperature (RT). The cells were washed and incubated overnight at 4 °C with an anti-myosin heavy chain 2 antibody (MF20, 1:100 dilution; R&D Systems, MN, USA) in 3% BSA. After this incubation, the cells were washed with PBS and then incubated with Alexa Fluor 488-conjugated secondary antibodies (ab150113, 1:400 dilution; Abcam, Cambridge, UK) at 25 °C. Following a final wash, the cell-seeded cover glass was prepared for the imaging process. All signals were observed using a THUNDER Imager 3D Assay microscope at 400× magnification (Leica, Wetzlar, Germany).

**Immunohistochemistry**

Immunohistochemistry of skeletal muscle sections was carried out using anti-myosin heavy chain type 2A, and 2B antibodies (DSHB, IA, USA), and an anti-laminin antibody (Abcam, Cambirdge, UK). Counterstaining was conducted with a 1 μg/mL of DAPI solution. Tibialis anterior (TA) muscles were sectioned at 8 μm thickness to measure the cross sectional area (CSA). Gastrocnemius muscles were sectioned at 10 μm thickness to analyze muscle fiber typing and distribution. Muscle fiber CSA and muscle fiber distribution were measured with the ImageJ 1.48 software (National Institutes of Health, USA) after the images were visualized with fluorescence microscopy (LEICA DM 2500). Liver tissue sectioning plus hematoxylin and eosin (H&E) staining was carried out by the Laboratory Animal Research Center (LARC), Gwangju Institute of Science and Technology.

**Molecular Docking Analysis**

The protein structures were obtained from the Protein Data Bank (https://www.rcsb.org/). The insulin receptor and glucocorticoid receptor (GR) structures were verified with PDB ID 1IR3 representing insulin receptor protein’s three-dimensional structure and 4UDC for the GR [4, 5]. PyMol (Schrodinger, New York, NY, USA) was utilized to remove the water molecules and extract the standard ligand from these structures. The compound structures were drawn with the ChemDraw 22.2.0 software (PerkinElmer, Waltham, MA, USA), and ligand energy minimization was performed with Chem 3D 21.1.0 software (PerkinElmer). The binding affinity between the receptors and ligands was analyzed with the molecular docking software Autodock Vina [6, 7]. Binding sites were selected based on their optimal orientation and alignment with the control ligand sites. The binding space center coordinates for the insulin receptor were X: -23.2434 Y: 28.2215, Z: 6.5822, and for the GR, X: -0.5704 Y: 40.0877, Z: -10.1242. The dimensions for both protein receptors were set at 25.00 Angstrom in the X, Y, and Z axes. The results were visualized with Discovery Studio Viewer software (BIOVIA, CA, USA).

**Surface plasmon resonance analysis**

Surface plasmon resonance (SPR) measurements were performed using an iMSPR-ProX instrument (iCLUEBiO, Republic of Korea). HC1000 chips coated with carboxymethyl-dextran were activated by injecting a solution of 200 mM EDC hydrochloride and 100 mM N-hydroxy succinimide across the surface at 20 µL/min for 7 min. To immobilize the ligand, recombinant insulin receptor (20 µg/mL in 5 mM sodium acetate, pH 4.0) was injected across flow cell 2 for 25 min at 10 µL/min, resulting in an immobilization level of approximately 9500 RU. Flow cell 1 served as the reference channel. Following immobilization, unreacted ester groups were blocked by injecting 1 M ethanolamine (HCl salt) at the previously set flow rate. SPR binding assays were conducted at 25 °C with a constant flow of 50 µL/min using running buffer composed of 10 mM phosphate, 140 mM NaCl, 2.7 mM KCl, 0.005 % Tween 20, and 1% DMSO, adjusted to pH 7.4. GmA (0.312–20 µM) was injected for a 180-second association phase, followed by a 720-second dissociation phase. Baseline-corrected sensorgrams were analyzed using iMSPR and TraceDrawer software and globally fitted to a 1:1 steady-state binding model to determine the dissociation constant (*K_d_*). Preliminary injections of phosphoaminophosphonic acid-adenylate ester (ANP) on the HC1000 surface produced responses below 5 RU. To improve ligand accessibility and allow reliable kinetic analysis, NiHC1000 NTA sensor chips were used. These chips were charged with 500 µM NiCl₂ for 90 s at 20 µL/min, and the insulin receptor was immobilized in two steps: first with 20 µg/mL for 40 min, then with 50 µg/mL for 16 min, both delivered at 10 µL/min. This resulted in approximately 10,000 RU of immobilized ligand. Binding studies were carried out in phosphate-buffered saline supplemented with 50 µM EDTA and 0.005% Tween 20 (pH 7.4) at a flow rate of 50 µL/min. ANP (62.5–1 000 µM) was injected for 150 s, followed by a 480 s dissociation phase. Recombinant His-tagged GR ligand-binding domain (GR-LBD) was captured on NiHC1000 sensor chips pre-charged with 500 µM NiCl₂ (20 µL/min, 90 s), resulting in ~5,300 RU on flow cell 2; flow cell 1 served as the reference. Assays were run at 25 °C in phosphate-buffered saline containing 140 mM NaCl, 2.7 mM KCl, 50 µM EDTA, 0.005% Tween-20, and 1% DMSO (pH 7.4), with a flow rate of 50 µL/min. GmA (0.312–10 µM; 180 s association, 720 s dissociation) and dexamethasone (25–400 µM; 150 s association, 420 s dissociation) were injected using a multi-cycle kinetic format. Kinetic constants were derived by fitting the data to a 1:1 Langmuir binding model as described above. Binding data represent duplicate injections performed on the same immobilized chip; values presented are averages of the two technical replicates.

**RNA-Seq**

RNA samples were obtained from C2C12 murine myoblasts cultured as follows: (1) differentiation media (DM) for 120 h; (2) DM for 96 h and DM plus 5 μM GmA for 24 h. RNA-Seq was carried out by Macrogen, Republic of Korea. Before the commencement of sequencing, quality control was undertaken with FastQC v0.11.7 (http:// www.bioinformatics.babraham.ac.uk/projects/fastqc/). Trimmomatic 0.38 with various parameters was used to trim the Illumina paired ends or single ends in the sequenced samples (http://www.usadellab.org/cms/?page=trimmomatic). The sequences of each sample were mapped and analyzed using HISAT2 version 2.1,0, Bowtie2 2.3.4.1 (https://ccb.jhu.edu/software/hisat2/index.shtml). StringTie version 2.1.3b (https://ccb.jhu.edu/software/stringtie/) was used to assemble potential transcripts and multiple splice variants. DESeq2 was used to generate the normalized sample count, and was also used for normalization, visualization, and differential analysis. The “apeglm” type was used to calculate shrink log2 fold changes.

**Glucose and Insulin Tolerance tests**

For the glucose tolerance test (GTT) and insulin tolerance test (ITT), 14-weeks-old male C57BL/6J mice were fasted for 6 h and 4 h, respectively (after 4 weeks treatment with daily IP 5 mg/kg GmA or vehicle alone; n=5 per group)*.* GTT was measured using an IP dose of 1.5 g/kg dextrose (Sigma-Aldrich). ITT was measured using an IP dose of 0.75 U human insulin (Sigma-Aldrich). Blood glucose levels were measured at the indicated time points via tail vein blood sampling with a glucose meter (Accu-Chek ® Instant S, IN, USA).

**Muscle Fatigue Test**

Muscle fatigue was measured in mice with two different models on a rotarod (Ugo Basile, Italy), as previously described [8]. There are two models present in the rotarod machine: the constant model and the accelerating model. In brief, the mice were accommodated in a training phase before starting the fatigue task using the rotarod. Mice were trained with speeds ramping from 10 rpm to 15 rpm. 24 h later, the muscle fatigue test was carried out with rotarod running at 5 rpm increments every 5 min up to 15 rpm*.* Latency to fall off the rotarod for each mouse was then measured. A fatigued mouse was classified falling off 4 times within 1 min, which then terminated the test.

**Muscle dissection and histological analysis**

Mice were anesthetized with 2.5% isoflurane (Hana, Republic of Korea) for 3 min before sacrifice (maintenance: 2%, induction: 2.5%). The quadriceps, gastrocnemius, tibialis anterior (TA), and soleus muscles were dissected and weighed. For immunohistochemistry, dissected muscles were sequentially embedded in 10%, 20% and 30% sucrose solution every 24 h at 4 °C, and then embedded into Cryo-OCT block for 24 h at 4 °C, followed by storage at -80 °C. Muscle sections were obtained using a Leica CM 1860 cryostat and mounted with DAPI Mount (Invitrogen).

**Supplementary Discussion**

Recently, there has been increasing appreciation that either the maintenance of muscle mass or enhancement of muscle function (strength) are preferable outcomes of therapeutic interventions, depending on the type of skeletal muscle atrophy being studied. Treatments that maintain muscle mass, rather than function, are thought to be more beneficial approaches for cancer cachexia, because increased mass alone can improve tolerance to chemotherapy [9]. Conversely, when sarcopenia treatments the effect on function is of more importance, due to the finding that the percentage loss of muscle strength is greater than mass loss in this disorder [10]. Our results indicate that GmA recovers muscle function in the rotarod test, but only significantly increased the mass of the TA muscle. Therefore, GmA may be more ideally suited for further development as a therapeutic for sarcopenia, rather than the types of muscle atrophy that benefit solely from muscle mass maintenance, such as cancer cachexia.

To confirm the biological mechanism of GmA, activation of the Akt/mTOR pathway downstream of the insulin receptor was assessed. At the protein level, insulin receptor β chain expression was decreased by Dex treatment and significantly recovered by GmA treatment in a dose-dependent manner. Furthermore, GmA treatment in the presence of Dex enhanced IGF-1 gene expression as shown by qPCR, and the insulin receptor kinase inhibitor AG1024 abolished the GmA-mediated restoration of receptor phosphorylation, indicating that GmA requires functional insulin receptor activity.

Previously, drugs used to treat type 2 diabetes have been investigated for repurposing as treatments for aging-related skeletal muscle atrophy, but the outcome have not always been favorable. For example, laboratory-based studies have shown that metformin, the most commonly prescribed drug for type 2 diabetes, blocks peptide absorption in the intestines and reduces myofiber CSA [11]. In addition, the sulfonylurea class of commonly prescribed anti-diabetes drugs have been linked to increased skeletal muscle atrophy in patients [12]. *G. inodorum* is a commonly used tea in Southeast Asia and previous human trials involving this plant extract have reported anti-diabetic effects [9, 11]. The results presented herein show that GmA is a bioactive compound *G. inodorum* that that prevents skeletal muscle atrophy. Moreover, the observation that GmA binds to the insulin receptor and activates the IGF-1/Akt pathway in skeletal muscle that is suppressed in insulin resistance and type 2 diabetes, suggests that GmA also contributes to the anti-diabetic effects of *G. inodorum* tea extract, although further studies in type 2 diabetes models, such as the db/db mouse, are required to confirm this dual bioactivity. Overall, GmA emerges as an interesting compound for further drug development for these two aging-related disorders, and may be useful in patients that have developed sarcopenic obesity (a combination of muscle loss and adipose tissue-derived inflammatory signals that can drive insulin resistance).

GmA treatment improved muscle performance in the Dex-treatment model of muscle atrophy, as shown by increased latency to fall in the rotarod test. This test is used assess walking speed, which is also a major diagnostic indicator for the progression of sarcopenia due to clinical assessments such as the 6 minutes walking distance [13]. The rotor rod test also measures balance and co-ordination, which is dependent on the integrity of the neuromuscular junctions between motor neurons and muscle fibers. Balance tests have also been used in assessments of sarcopenia [14]. Of note, while the 2.5 and 5 mg/kg doses of GmA did not consistently increase the mass of the major leg muscles in the Dex model (quadriceps and gastrocnemius), the mass of the TA muscle was increased. The TA muscle is composed primarily of glycolytic type II fast myofibers that are preferentially lost in sarcopenia. In contrast, the quadriceps and gastrocnemius muscles are mixed myofibers types, containing slow oxidative type I myofibers in addition to the subpopulations of type II fast myofibers. This difference may explain why GmA produced a significant recovery of TA muscle mass. Although GmA did not significantly increase gastrocnemius muscle mass, treatment did reduce the expression of the atrogenes atrogin-1 and Murf-1 expression, an upregulate IGF-1 expression, and normalize expression of the autophagy-related genes LC3 and p62. These results suggest that although GmA may not significantly increase mass in all muscles tested, it effectively inhibits the signaling pathways that produce atrophy, thereby contributing to functional recovery.

**References**

1. An, J.-P., et al., *Oleanane Triterpenoids from the Leaves of Gymnema inodorum and Their Insulin Mimetic Activities.* Journal of Natural Products, 2020. **83**(4): p. 1265-1274.

2. Lee, S.H., et al., *Modulating phosphatase DUSP22 with BML-260 ameliorates skeletal muscle wasting via Akt independent JNK-FOXO3a repression.* EMBO Mol Med, 2025. **17**(6): p. 1259-1288.

3. Kim, H.-J., et al., *Investigation of niclosamide as a repurposing agent for skeletal muscle atrophy.* PLOS ONE, 2021. **16**(5): p. e0252135.

4. Singh, N., V. Dalal, and P. Kumar, *Molecular docking and simulation analysis for elucidation of toxic effects of dicyclohexyl phthalate (DCHP) in glucocorticoid receptor-mediated adipogenesis.* Molecular Simulation, 2020. **46**(1): p. 9-21.

5. Ganugapati, J., A. Baldwa, and S. Lalani, *Molecular docking studies of banana flower flavonoids as insulin receptor tyrosine kinase activators as a cure for diabetes mellitus.* Bioinformation, 2012. **8**(5): p. 216-20.

6. Trott, O. and A.J. Olson, *AutoDock Vina: Improving the speed and accuracy of docking with a new scoring function, efficient optimization, and multithreading.* Journal of Computational Chemistry, 2010. **31**(2): p. 455-461.

7. Eberhardt, J., et al., *AutoDock Vina 1.2.0: New Docking Methods, Expanded Force Field, and Python Bindings.* Journal of Chemical Information and Modeling, 2021. **61**(8): p. 3891-3898.

8. Lee, J.-H., et al., *Inhibited inositol monophosphatase and decreased myo-inositol concentration improve wasting in skeletal muscles.* Clinical and Translational Medicine, 2020. **10**(8): p. e251.

9. Lambert, C.P., *Should the FDA's criteria for the clinical efficacy of cachexia drugs be changed? Is Ostarine safe and effective?* J Cachexia Sarcopenia Muscle, 2021. **12**(3): p. 531-532.

10. Cruz-Jentoft, A.J. and A.A. Sayer, *Sarcopenia.* Lancet, 2019. **393**(10191): p. 2636-2646.

11. Kang, M.J., et al., *Metformin induces muscle atrophy by transcriptional regulation of myostatin via HDAC6 and FoxO3a.* J Cachexia Sarcopenia Muscle, 2022. **13**(1): p. 605-620.

12. Mele, A., et al., *Database search of spontaneous reports and pharmacological investigations on the sulfonylureas and glinides-induced atrophy in skeletal muscle.* Pharmacol Res Perspect, 2014. **2**(1): p. e00028.

13. Xie, W.Q., et al., *Mouse models of sarcopenia: classification and evaluation.* J Cachexia Sarcopenia Muscle, 2021. **12**(3): p. 538-554.

14. Khanal, P., et al., *Static one-leg standing balance test as a screening tool for low muscle mass in healthy elderly women.* Aging Clin Exp Res, 2021. **33**(7): p. 1831-1839.
